# Synthesis of Trifluoromethylated Spiroisoxazolones via a [3+2] Cycloaddition of Nitrile Imines and Unsaturated Isoxazolones

**DOI:** 10.3390/molecules31010073

**Published:** 2025-12-24

**Authors:** Wei Zhang, Da-Ming Du

**Affiliations:** 1 School of Chemistry and Chemical Engineering, Beijing Institute of Technology, Beijing 100081, China; 3220231751@bit.edu.cn; 2 Key Laboratory of Medicinal Molecule Science and Pharmaceutical Technology, Ministry of Industry and Information Technology, Beijing 100081, China

**Keywords:** nitrile imine, unsaturated isoxazolidinone, spirocyclic compounds, fluorine-containing heterocycles, 1,3-dipole, [3+2] cycloaddition

## Abstract

A strategy for constructing trifluoromethylated spiroisoxazolones has been developed. This approach relies on the 1,3-dipolar cycloaddition of CF_3_-substituted nitrile imines, generated in situ from trifluoroacetyl hydrazonoyl bromides and K_2_CO_3_, with the exocyclic double bond of 4-benzylidene-3-methylisoxazol-5(4*H*)-ones. The reaction provides a series of trifluoromethylated spiro(isoxazolone-pyrazoline) derivatives in moderate to high yields (up to 93%). The protocol exhibits broad substrate compatibility with respect to aromatic substituents on both reaction partners. To the best of our knowledge, the introduction of a trifluoromethyl group at the 3-position of the pyrazoline ring via nitrile imine cycloaddition chemistry has not been previously reported. The resulting products incorporate a valuable CF_3_-substituted pyrazoline pharmacophore spiro-fused to an isoxazolone core and may be of interest for medicinal chemistry programs.

## 1. Introduction

Spirocyclic compounds are ring systems characterized by two cyclic structures connected through a common spiro carbon atom. This unique architectural feature imparts high conformational rigidity and a stable three-dimensional configuration, endowing spiro scaffolds with physicochemical properties, metabolic behaviors, and biological activities distinct from those of planar aromatic systems or flexible chain structures [1,2,3]. In medicinal chemistry, the incorporation of a spirocyclic framework serves as a valuable strategy for optimizing key drug-like properties [4,5,6,7,8]. This includes the modulation of lipophilicity, enhancement of aqueous solubility, stabilization of bioactive conformations, improvement of target selectivity, and fine-tuning of pharmacokinetic profiles [9,10,11].

**Figure 1:**
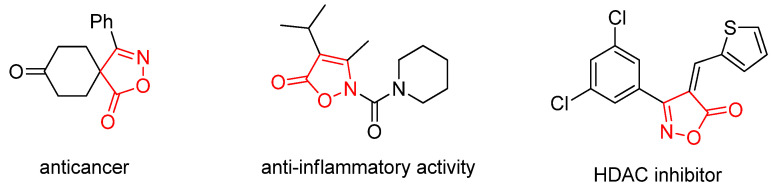
Examples of biologically isoxazolone derivatives.

Isoxazolone and its derivatives represent an important class of five-membered heterocyclic compounds containing both nitrogen and oxygen atoms. They serve as crucial pharmacophores and structural motifs found in numerous natural products and pharmaceuticals [12,13,14]. The distinctive architecture of the isoxazolone ring enables it to engage in diverse non-covalent interactions, notably as a hydrogen-bond acceptor (via its N and O atoms), π-π stacking (through its unsaturated ring system), and hydrophilic interactions. This capacity to interact with various protein targets underpins the broad spectrum of biological activities exhibited by isoxazolone-based compounds, leading to their widespread application in medicinal and agrochemical fields. A multitude of both natural and synthetic isoxazolone derivatives have been investigated as active agents for various therapeutic purposes, including anticancer, anti-inflammatory, antidiabetic, and antiviral applications (Figure 1) [15,16,17]. In addition, incorporating a trifluoromethyl group serves as a strategic medicinal chemistry tactic to simultaneously enhance key pharmacological properties. Its strong electron-withdrawing effect and high lipophilicity improve metabolic stability, membrane permeability, and target binding affinity, while its role as a versatile bioisostere allows for the fine-tuning of molecular interactions, a principle validated by numerous successful therapeutics [18,19,20,21].

**Scheme 1:**
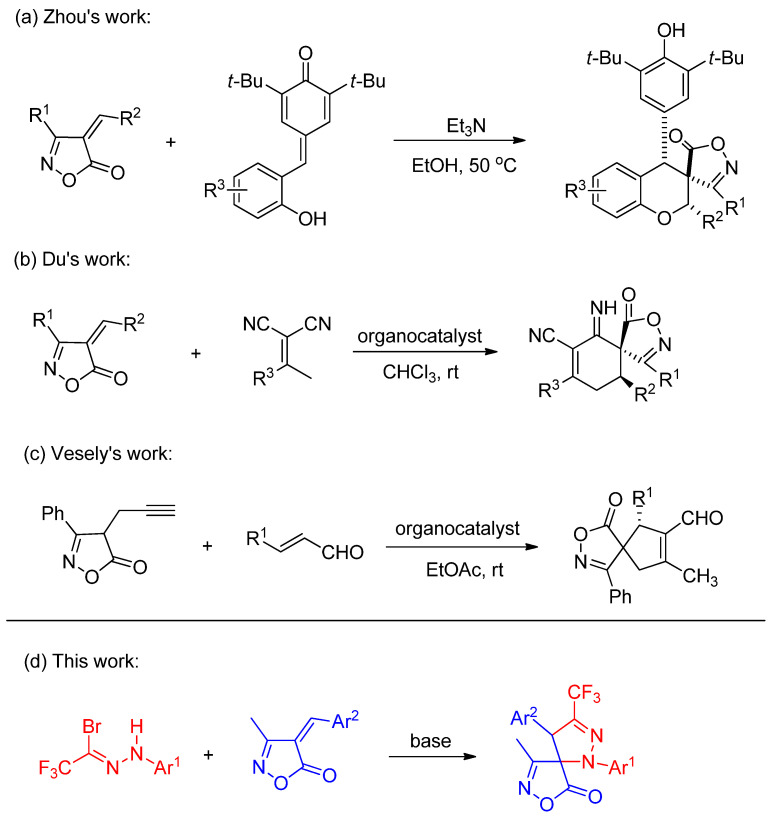
Examples of spiroisoxazolidinone synthesis and our work. (**a**) addition-annulation reaction [25]; (**b**) asymmetric Michael addition-annulation cascade [26]; (**c**) asymmetric Conia-ene-type cascade reaction [27]; (**d**) our research plan.

Given the considerable potential demonstrated by spiroisoxazolone compounds in medicinal chemistry, recent years have witnessed growing research interest in the multi-site modification of isoxazolones [22,23,24]. In 2019, Zhou and co-workers reported a series of spiroisoxazolone-chromane derivatives via an addition-annulation reaction between ortho-hydroxyphenyl-substituted p-quinone analogs and 4-alkylideneisoxazol-5-ones (Scheme 1a) [25]. Subsequently, in 2021, Du’s team described an asymmetric Michael addition-annulation cascade between 4-alkylideneisoxazol-5-ones and dicyanoalkenes, leading to a series of isoxazolone-based spirocyclic derivatives (Scheme 1b) [26]. More recently, in 2025, Vesely’s group achieved the synthesis of chiral spiroisoxazolones through an asymmetric Conia-ene-type cascade reaction of α,β-unsaturated aldehydes with propargyl-substituted isoxazolones (Scheme 1c) [27]. Motivated by these advancements and the fact that the introduction of a trifluoromethyl group at the pyrazoline 3-position via nitrile imine cycloaddition remains unexplored, we report herein the 1,3-dipolar cycloaddition of in situ-generated CF_3_-substituted nitrile imines (from trifluoroacetyl hydrazonoyl bromides) with 4-benzylidene-3-methylisoxazol-5(4*H*)-ones.

## 2. Results and Discussion

### 2.1. Optimization of Reaction Conditions

The 1,3-dipolar cycloaddition of trifluoromethyl-substituted nitrile imines with 4-benzylidene-3-methylisoxazol-5(4*H*)-ones was investigated. The required nitrile imines were generated in situ from trifluoroacetyl hydrazonoyl bromides by treatment with K_2_CO_3_. Using 4-benzylidene-3-methylisoxazol-5(4*H*)-one (**1a**) and 2,2,2-trifluoro-*N*′-phenylacetohydrazonoyl bromide (**2a**) as model substrates, the reaction in THF at room temperature for 24 h afforded a mixture of *syn*-**3a** and *anti*-**3a** in 52% combined yield with a diastereomeric ratio of 1.7:1 (Table 1, entry 1). To improve the reaction efficiency, we screened various solvents. Gratifyingly, the use of 1,2-dichloroethane (DCE) significantly enhanced the yield to 78% (Table 1, entry 3). Solvent polarity may affect dipole stability, with DCE proving optimal due to its low polarity in this system. Subsequent evaluation of different bases confirmed K_2_CO_3_ as the most effective. The use of soluble bases such as triethylamine leads to rapid deprotonation of the hydrazonoyl bromide, resulting in a high transient concentration of the nitrile imine dipole, which promotes side processes like dimerization and decomposition, thereby reducing the yield and diastereoselectivity. In contrast, insoluble bases like K_2_CO_3_ facilitate slow and controlled generation of the dipole, maintaining a low stationary concentration that minimizes these undesired reactions and enhances the efficiency of the desired cycloaddition. The reaction temperature was also optimized, revealing that both elevating to 50 °C and lowering to 0 °C resulted in diminished yields. Finally, screening of substrate stoichiometry demonstrated that a molar ratio of **1a**/**2a**/base = 1.2:1:1.2 further increased the yield to 92% (Table 1, entry 10). The use of 1.2 equivalents of the nitrile imine precursor provided optimal yields by compensating for its dimerization during the reaction, ensuring sufficient reactive dipole remains available for cycloaddition; however, increasing to 1.5 equivalents led to reduced yields, likely due to elevated dipole concentration exacerbating side reactions such as further dimerization. Consequently, the optimized conditions were established as follows: **1a**/**2a**/base molar ratio of 1.2:1:1.2, K_2_CO_3_ as base, in 1,2-dichloroethane at room temperature for 24 h.

### 2.2. Substrate Scope

**Scheme 2:**
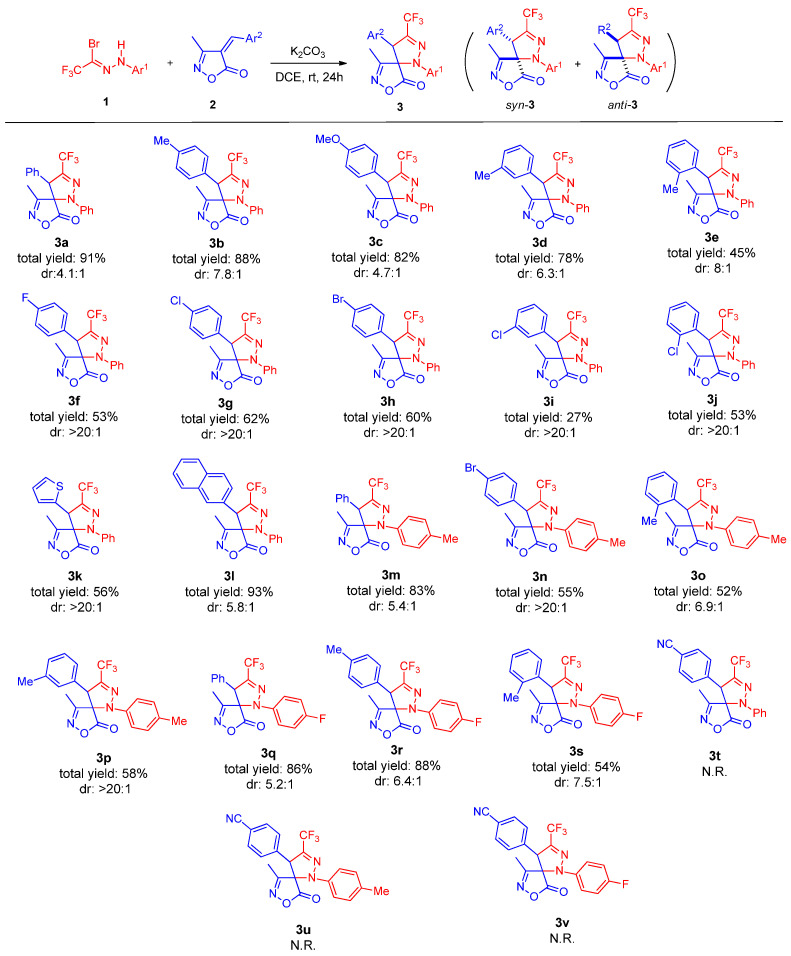
Substrate scope of arylidene isoxazol-5(4*H*)-one **1** and trifluoroacetyl bromohydrazone **2**. Unless noted otherwise, the reactions were carried out with **1** (0.48 mmol), **2** (0.40 mmol), and K_2_CO_3_ (0.48 mmol) in DCE (2.0 mL) at rt for 24 h. The yields were isolated after column chromatography. Diastereomeric ratio was determined by ^1^H NMR analysis of the crude reaction mixture.

Under the optimized conditions, the substrate scope of this reaction was investigated, as shown in Scheme 2. We first explored the variation in the unsaturated isoxazolone component. The reaction proceeded efficiently when the aromatic ring (Ar^2^) bore electron-donating groups (e.g., methyl or methoxy) at the *para*- or *meta*-positions, affording products **3a**–**3c** in 78–88% yields. In contrast, the presence of these substituents at the *ortho*-position led to a significant decrease in yield (**3d**, 45%), presumably due to steric hindrance. Substrates with electron-withdrawing groups (–F, –Br, –Cl) on the Ar^2^ aromatic ring were also well tolerated, affording products **3f**–**3g** in moderately reduced yields. Notably, the reaction produced solely the *syn*-diastereomer. Replacing the phenyl ring with a 2-thienyl group was feasible, providing the product **3k** in 56% yield. Remarkably, the introduction of a naphthyl group at Ar^2^ resulted in an excellent yield of 93% for product **3l**. When a strongly electron-withdrawing 4-cyanophenyl group was introduced at the Ar^2^ (for compounds **3t**–**3v**), no cycloaddition took place under the optimized conditions, even after heating at 50 °C for 48 h. TLC monitoring and ^1^H NMR analysis of the crude mixture showed no formation of any cycloadduct (neither the desired regioisomer nor its alternative). This complete lack of reactivity is attributed to the significantly reduced dipolarophilicity of the alkene caused by the 4-cyanophenyl substituent, a phenomenon analogous to that observed for 4-nitrophenyl-substituted 4-arylideneoxazol-5(4*H*)-ones in related nitrile imine cycloadditions [28].

Subsequently, the scope concerning the trifluoromethyl bromohydrazone component (Ar^1^ aromatic ring) was examined. A variety of substituents were compatible with the reaction conditions. Both electron-donating (4-CH_3_) and electron-withdrawing groups (4-F) on the Ar^1^ aryl ring proceeded smoothly, furnishing the desired spirocyclic products **3m**–**3p** or **3q**–**3s** in yields comparable to those obtained with the parent phenyl-derived substrate.

### 2.3. Scaled-Up Synthesis

**Scheme 3:**
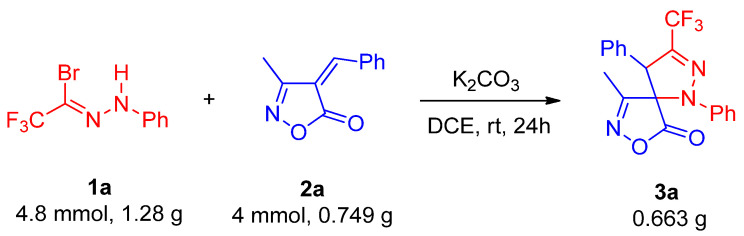
Gram-scale synthesis of **3a**.

Subsequently, to evaluate the industrial applicability of this reaction, a gram-scale synthesis was performed by scaling up the reaction by tenfold, which afforded product **3a** in 83% overall yield with 3.8:1 dr (Scheme 3).

### 2.4. X-Ray Crystal Structure

**Figure 2:**
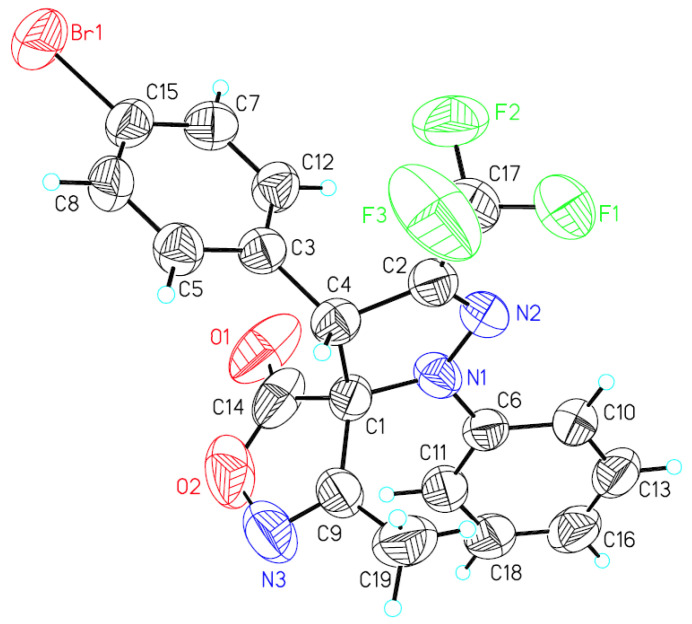
X-ray crystal structure of *syn*-**3h**.

A single crystal of compound *syn*-**3h** was obtained by crystallization from petroleum ether and dichloromethane (8:1 *v*/*v*) at room temperature. The relative configuration of *syn*-**3h** was determined by X-ray crystallography analysis [29] as (rel-4*S*, 5*R*) (Figure 2), the relative configuration of other *syn*-products were assigned analogously.

### 2.5. Reaction Mechanism

To investigate the origin of the observed diastereoselectivity, the starting isoxazolone **1a** was monitored by ^1^H NMR in CDCl_3_ under two sets of conditions. In the absence of base, no formation of the (*E*)-isomer was detected at room temperature over 24 h. When 1.2 equivalents of anhydrous K_2_CO_3_ were added for 24 h to simulate the reaction conditions, the exocyclic double bond geometry remained exclusively (*Z*) throughout, with no evidence of isomerization. These results clearly indicate that the (Z)-configuration of the dipolarophile is configurationally stable under the cycloaddition conditions. Consequently, the stereochemical outcome must be controlled after the initial irreversible C–C bond-forming step rather than by pre-equilibration of the alkene conformers.

**Scheme 4:**
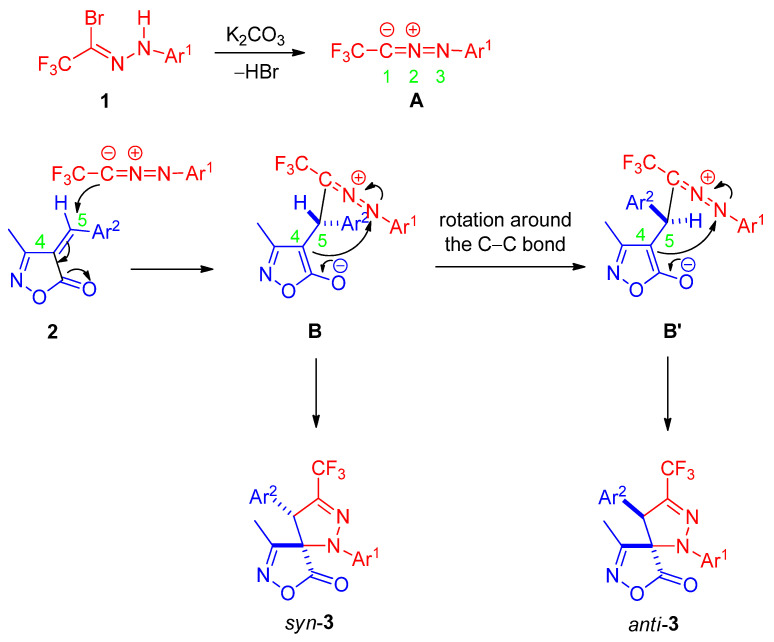
Proposed reaction mechanism.

A plausible reaction mechanism consistent with these observations is proposed in Scheme 4. The reaction commences with K_2_CO_3_-promoted dehydrobromination of bromohydrazone **1** to generate nitrile imine intermediate **A** in situ. Regioselective [3+2] cycloaddition of **A** onto the (*Z*)-configured unsaturated isoxazolone **2** affords zwitterionic intermediate **B**. Rotation around the newly formed anionic C–C single bond in **B** can produce rotamer **B′**, differing in the spatial arrangement of the substituents. Subsequent intramolecular nucleophilic attack of the C4 enolate on the iminium nitrogen (N3) then yields the *syn*-diastereomer *syn*-**3** predominantly from **B**, while the minor *anti*-diastereomer *anti*-**3** is generated from **B′**.

## 3. Materials and Methods

### 3.1. General Information

Commercially available compounds were used without further purification. Solvents were dried according to standard procedures. Column chromatography was performed with silica gel (200–300 mesh) (Yantai, China). Melting points were determined with a WRX-4 melting-point apparatus (Shanghai, China) and are uncorrected. ^1^H NMR spectra were measured with a Bruker Ascend 500 MHz or 400 MHz spectrometer (Karlsurhe, Germany); chemical shifts were reported in *δ* (ppm) units relative to tetramethylsilane (TMS) as the internal standard. ^13^C NMR spectra were measured at 126 MHz with a 500 MHz spectrometer or at 101 MHz with a 400 MHz spectrometer; chemical shifts are reported in ppm relative to tetramethylsilane and referenced to the solvent peak (CDCl_3_, δC = 77.00). The ^19^F NMR spectra were measured at 471 MHz or 376 MHz. Proton coupling patterns are intricately characterized as diverse spectroscopic signatures, ranging from singlets (s) to doublets (d), triplets (t), quartets (q), multiplets (m), and broadened signals (br s). High-resolution mass spectra were acquired using the Agilent 6520 accurate-mass-Q-TOF MS system (Beijing, China), which incorporates an electrospray ionization (ESI+) source.

### 3.2. Experimental Materials

A series of unsaturated isoxazolones were synthesized according to literature procedures [30,31,32], while the trifluoromethyl bromohydrazones were synthesized according to literature procedures [33,34].

### 3.3. General Procedure for the Synthesis of Spiroisoxazolones ***3***

To a solution of K_2_CO_3_ (0.4 mmol) in 1,2-dichloroethane (2 mL) was added trifluoromethyl bromohydrazone **1** (0.48 mmol, 1.2 equiv) and unsaturated isoxazolone derivative **2** (0.4 mmol, 1.0 equiv). The reaction mixture was stirred at room temperature for 24 h. Upon completion, the solvent was removed under reduced pressure, and the residue was purified by flash chromatography on silica gel (eluent: petroleum ether/ethyl acetate = 20:1, *v*/*v*) to afford product **3**.

(rel-4*S*,5*R*)-9-Methyl-1,4-diphenyl-3-(trifluoromethyl)-7-oxa-1,2,8-triazaspiro[4.4]nona-2,8-dien-6-one (*syn*-**3a**). The synthesis of this compound was conducted using a standardized protocol, followed by purification through column chromatography on silica gel (200–300 mesh) with petroleum ether/ethyl acetate (20:1 *v*/*v*) as the eluent to give 109.0 mg, 73% yield, yellow oil; ^1^H NMR (500 MHz, CDCl_3_): δ 7.41–7.36 (m, 3H, ArH), 7.32–7.28 (m, 2H, ArH), 7.19–7.17 (m, 2H, ArH), 7.07 (t, *J* = 7.5 Hz, 1H, ArH), 7.00 (dd, *J*_1_ = 9.0 Hz, *J*_2_ = 0.8 Hz, 2H, ArH), 4.85 (s, 1H, CH), 2.18 (s, 3H, CH_3_) ppm; ^13^C NMR (126 MHz, CDCl_3_): δ 169.8, 165.5, 141.3, 138.5 (q, ^2^*J*_C–F_ = 37.9 Hz), 130.1, 129.9, 129.4, 129.1, 128.4, 123.9, 119.9 (q, ^1^*J*_C–F_ = 272.0 Hz), 114.9, 77.3, 60.6, 11.8 ppm; ^19^F NMR (471 MHz, CDCl_3_): δ −62.3 (s, 3F) ppm. HRMS (ESI): *m/z* calcd. for C_19_H_15_F_3_N_3_O_2_ [M + H]^+^ 374.1111, found 374.1142.

(rel-4*R*,5*R*)-9-Methyl-1,4-diphenyl-3-(trifluoromethyl)-7-oxa-1,2,8-triazaspiro[4.4]nona-2,8-dien-6-one (*anti*-**3a**). The synthesis of this compound was conducted using a standardized protocol, followed by purification through column chromatography on silica gel (200–300 mesh) with petroleum ether/ethyl acetate (20:1 *v*/*v*) as the eluent to give 26.9 mg, 18% yield, yellow solid; m.p. 192–194 °C; ^1^H NMR (500 MHz, CDCl_3_): δ 7.44 (t, *J* = 2.5 Hz, 3H, ArH), 7.33–7.29 (m, 2H, ArH), 7.23–7.21 (m, 2H, ArH), 7.09 (t, *J* = 7.5 Hz, 1H, ArH), 6.99 (d, *J* = 8.0 Hz, 2H, ArH), 5.16 (s, 1H, CH), 1.38 (s, 3H, CH_3_) ppm; ^13^C NMR (126 MHz, CDCl_3_): δ 169.8, 165.5, 141.3, 138.5 (q, ^2^*J*_C–F_ = 37.8 Hz), 130.0, 129.9, 129.3, 129.1, 128.4, 123.9, 119.9 (q, ^1^*J*_C–F_ = 272.0 Hz), 114.9, 77.3, 60.5 11.8 ppm; ^19^F NMR (471 MHz, CDCl_3_): δ −62.3 (s, 3F) ppm. HRMS (ESI): *m/z* calcd. for C_19_H_15_F_3_N_3_O_2_ [M + H]^+^ 374.1111, found 374.1141.

(rel-4*S*,5*R*)-9-Methyl-1-phenyl-4-(*p*-tolyl)-3-(trifluoromethyl)-7-oxa-1,2,8-triazaspiro[4.4]nona-2,8-dien-6-one (*syn*-**3b**). The synthesis of this compound was conducted using a standardized protocol, followed by purification through column chromatography on silica gel (200–300 mesh) with petroleum ether/ethyl acetate (20:1 *v*/*v*) as the eluent to give 117.7 mg, 78% yield, yellow solid; m.p. 202–204 °C; ^1^H NMR (500 MHz, CDCl_3_): δ 7.32–7.29 (m, 2H, ArH), 7.19 (d, *J* = 8.0 Hz, 2H, ArH), 7.08–7.05 (m, 3H, ArH), 6.99 (d, *J* = 7.5 Hz, 2H, ArH), 4.83 (s, 1H, CH), 2.35 (s, 3H, CH_3_), 2.19 (s, 3H, CH_3_) ppm; ^13^C NMR (126 MHz, CDCl_3_): δ 169.9, 165.6, 141.3, 140.2, 138.6 (q, ^2^*J*_C–F_ = 37.6 Hz), 129.9, 129.8, 129.2, 125.3, 123.8, 119.9 (q, ^1^*J*_C–F_ = 272.0 Hz), 114.9, 77.4, 60.4, 21.3, 11.8 ppm; ^19^F NMR (471 MHz, CDCl_3_): δ −62.3 (s, 3F) ppm. HRMS (ESI): *m/z* calcd. for C_20_H_17_F_3_N_3_O_2_ [M + H]^+^ 388.1267, found 388.1255.

(rel-4*R*,5*R*)-9-Methyl-1-phenyl-4-(*p*-tolyl)-3-(trifluoromethyl)-7-oxa-1,2,8-triazaspiro[4.4]nona-2,8-dien-6-one (*anti*-**3b**). The synthesis of this compound was conducted using a standardized protocol, followed by purification through column chromatography on silica gel (200–300 mesh) with petroleum ether/ethyl acetate (20:1 *v*/*v*) as the eluent to give 15.5 mg, 10% yield, yellow oil; ^1^H NMR (500 MHz, CDCl_3_): δ 7.31 (t, *J* = 7.8 Hz, 2H, ArH), 7.23 (d, *J* = 8.0 Hz, 2H, ArH), 7.10–7.06 (m, 3H, ArH), 6.98 (d, *J* = 8.0 Hz, 2H, ArH), 5.13 (s, 1H, CH), 2.37 (s, 3H, CH_3_), 1.41 (s, 3H, CH_3_) ppm; ^13^C NMR (126 MHz, CDCl_3_): δ 174.5, 164.7, 141.3, 140.2, 138.9 (q, ^2^*J*_C–F_ = 38.2 Hz), 130.2, 129.9, 128.6, 126.5, 124.1, 120.0 (q, ^1^*J*_C–F_ = 272.3 Hz), 115.2, 77.3, 62.7, 21.2, 13.6 ppm; ^19^F NMR (471 MHz, CDCl_3_): δ −62.3 (s, 3F) ppm. HRMS (ESI): *m/z* calcd. for C_20_H_17_F_3_N_3_O_2_ [M + H]^+^ 388.1267, found 388.1273.

(rel-4*S*,5*R*)-4-(4-Methoxyphenyl)-9-methyl-1-phenyl-3-(trifluoromethyl)-7-oxa-1,2,8-triazaspiro[4.4]nona-2,8-dien-6-one (*syn*-**3c**). The synthesis of this compound was conducted using a standardized protocol, followed by purification through column chromatography on silica gel (200–300 mesh) with petroleum ether/ethyl acetate (20:1 *v*/*v*) as the eluent to give 108.1 mg, 67% yield, brown oil; ^1^H NMR (500 MHz, CDCl_3_): δ 7.32–7.29 (m, 2H, ArH), 7.11 (d, *J* = 9.0 Hz, 2H, ArH), 7.07 (t, *J* = 7.5 Hz, 1H, ArH), 6.99 (d, *J* = 8.0 Hz, 2H, ArH), 6.89 (d, *J* = 9.0 Hz, 2H, ArH), 4.84 (s, 1H, CH), 3.79 (s, 3H, OCH_3_), 2.18 (s, 3H, CH_3_) ppm; ^13^C NMR (126 MHz, CDCl_3_): δ 170.0, 165.6, 160.8, 141.4, 138.7 (q, ^2^*J*_C–F_ = 37.5 Hz), 130.7, 129.9, 123.2, 120.0, 119.9 (q, ^1^*J*_C–F_ = 272.0 Hz), 114.8, 114.5, 77.3, 60.2, 55.2, 11.8 ppm; ^19^F NMR (471 MHz, CDCl_3_): δ −62.3 (s, 3F) ppm. HRMS (ESI): *m/z* calcd. for C_20_H_17_F_3_N_3_O_3_ [M + H]^+^ 404.1217, found 404.1210.

(rel-4*R*,5*R*)-4-(4-Methoxyphenyl)-9-methyl-1-phenyl-3-(trifluoromethyl)-7-oxa-1,2,8-triazaspiro[4.4]nona-2,8-dien-6-one (*anti*-**3c**). The synthesis of this compound was conducted using a standardized protocol, followed by purification through column chromatography on silica gel (200–300 mesh) with petroleum ether/ethyl acetate (20:1 *v*/*v*) as the eluent to give 24.2 mg, 15% yield, colorless oil; ^1^H NMR (500 MHz, CDCl_3_): δ 7.32–7.29 (m, 2H, ArH), 7.14 (d, *J* = 8.5 Hz 2H, ArH), 7.08 (t, *J* = 7.5 Hz, 1H, ArH), 6.98 (d, *J* = 7.5 Hz, 2H, ArH), 6.95–6.92 (m, 2H, ArH), 5.13 (s, 1H, CH), 3.83 (s, 3H, OCH_3_), 1.42 (s, 3H, CH_3_) ppm; ^13^C NMR (126 MHz, CDCl_3_): δ 174.5, 164.8, 160.7, 141.3, 138.9 (q, ^2^*J*_C–F_ = 38.1 Hz), 130.0, 129.9, 124.1, 121.3, 120.0 (q, ^1^*J*_C–F_ = 272.1 Hz), 115.2, 114.9, 77.2, 62.4, 55.4, 13.6 ppm; ^19^F NMR (471 MHz, CDCl_3_): δ −62.3 (s, 3F) ppm. HRMS (ESI): *m/z* calcd. for C_20_H_17_F_3_N_3_O_3_ [M + H]^+^ 404.1217, found 404.1210.

(rel-4*S*,5*R*)-9-Methyl-1-phenyl-4-(*m*-tolyl)-3-(trifluoromethyl)-7-oxa-1,2,8-triazaspiro[4.4]nona-2,8-dien-6-one (*syn*-**3d**). The synthesis of this compound was conducted using a standardized protocol, followed by purification through column chromatography on silica gel (200–300 mesh) with petroleum ether/ethyl acetate (20:1 *v*/*v*) as the eluent to give 103.8 mg, 67% yield, yellow oil; ^1^H NMR (500 MHz, CDCl_3_): δ 7.32–7.28 (m, 2H, ArH), 7.26–7.24 (m, 1H, ArH), 7.20 (d, *J* = 7.5 Hz, 1H, ArH), 7.06 (t, *J* = 7.3 Hz, 1H, ArH), 7.00 (t, *J* = 8.5 Hz, 4H, ArH), 4.81 (s, 1H, CH), 2.34 (s, 3H, CH_3_), 2.18 (s, 3H, CH_3_) ppm; ^13^C NMR (126 MHz, CDCl_3_): δ 169.8, 165.5, 141.3, 138.9, 138.6 (q, ^2^*J*_C–F_ = 37.7 Hz), 130.8, 129.92, 129.86, 128.9, 128.3, 126.4, 123.9, 119.9 (q, ^1^*J*_C–F_ = 272.0 Hz), 114.9, 77.3, 60.6, 21.3, 11.8 ppm; ^19^F NMR (471 MHz, CDCl_3_): δ −62.3 (s, 3F) ppm. HRMS (ESI): *m/z* calcd. for C_20_H_17_F_3_N_3_O_2_ [M + H]^+^ 388.1267, found 388.1272.

(rel-4*R*,5*R*)-9-Methyl-1-phenyl-4-(*m*-tolyl)-3-(trifluoromethyl)-7-oxa-1,2,8-triazaspiro[4.4]nona-2,8-dien-6-one (*anti*-**3d**). The synthesis of this compound was conducted using a standardized protocol, followed by purification through column chromatography on silica gel (200–300 mesh) with petroleum ether/ethyl acetate (20:1 *v*/*v*) as the eluent to give 17.0 mg, 11% yield, yellow oil; ^1^H NMR (500 MHz, CDCl_3_): δ 7.33–7.29 (m, Hz, 3H, ArH), 7.24 (d, *J* = 8.0 Hz, 2H, ArH), 7.08 (d, *J* = 7.5 Hz, 1H, ArH), 7.02–6.98 (m, 4H, ArH), 5.11 (s, 1H, CH), 2.37 (s, 3H, CH_3_), 1.40 (s, 3H, CH_3_) ppm; ^13^C NMR (126 MHz, CDCl_3_): δ 174.5, 164.7, 141.3, 139.6, 138.9 (q, ^2^*J*_C–F_ = 38.2 Hz), 130.8, 129.8, 129.5, 129.4, 129.3, 125.7, 124.1, 119.9 (q, ^1^*J*_C–F_ = 271.6 Hz), 118.9, 115.3, 77.2, 62.8, 21.4, 13.5 ppm; ^19^F NMR (471 MHz, CDCl_3_): δ −61.8 (s, 3F) ppm. HRMS (ESI): *m/z* calcd. for C_20_H_17_F_3_N_3_O_2_ [M + H]^+^ 388.1267, found 388.1264.

(rel-4*S*,5*R*)-9-Methyl-1-phenyl-4-(*o*-tolyl)-3-(trifluoromethyl)-7-oxa-1,2,8-triazaspiro[4.4]nona-2,8-dien-6-one (*syn*-**3e**). The synthesis of this compound was conducted using a standardized protocol, followed by purification through column chromatography on silica gel (200–300 mesh) with petroleum ether/ethyl acetate (20:1 *v*/*v*) as the eluent to give 62.0 mg, 40% yield, yellow solid; m.p. 156–158 °C; ^1^H NMR (500 MHz, CDCl_3_): δ 7.32–7.26 (m, 4H, ArH), 7.24–7.22 (m, 1H, ArH), 7.16 (d, *J* = 7.0 Hz, 1H, ArH), 7.07 (t, *J* = 7.2 Hz 1H, ArH), 6.98 (d, *J* = 8.5 Hz, 2H, ArH), 5.16 (s, 1H, CH), 2.30 (s, 3H, CH_3_), 2.16 (s, 3H, CH_3_) ppm; ^13^C NMR (126 MHz, CDCl_3_): δ 169.8, 165.6, 141.1, 139.2 (q, ^2^*J*_C–F_ = 37.8 Hz), 135.6, 131.1, 130.5, 129.9, 129.8, 126.9, 126.5, 123.8, 119.9 (q, ^1^*J*_C–F_ = 271.8 Hz), 114.7, 75.8, 56.1, 19.5, 11.8 ppm; ^19^F NMR (471 MHz, CDCl_3_): δ −62.3 (s, 3F) ppm. HRMS (ESI): *m/z* calcd. for C_20_H_17_F_3_N_3_O_2_ [M + H]^+^ 388.1267, found 388.1264.

(rel-4*R*,5*R*)-9-Methyl-1-phenyl-4-(*o*-tolyl)-3-(trifluoromethyl)-7-oxa-1,2,8-triazaspiro[4.4]nona-2,8-dien-6-one (*anti*-**3e**). The synthesis of this compound was conducted using a standardized protocol, followed by purification through column chromatography on silica gel (200–300 mesh) with petroleum ether/ethyl acetate (20:1 *v*/*v*) as the eluent to give 7.7 mg, 5% yield, yellow solid; m.p. 131–133 °C; ^1^H NMR (500 MHz, CDCl_3_): δ 7.34–7.26 (m, 5H, ArH), 7.17 (d, *J* = 8.0 Hz, 1H, ArH), 7.08 (t, *J* = 7.2 Hz, 1H, ArH), 6.98 (d, *J* = 8.0 Hz, 2H, ArH), 5.37 (s, 1H, CH), 2.31 (s, 3H, CH_3_), 1.31 (s, 3H, CH_3_) ppm; ^13^C NMR (126 MHz, CDCl_3_): δ 174.3, 165.6, 141.4, 139.6 (q, ^2^*J*_C–F_ = 38.0 Hz), 136.9, 131.9, 130.0, 129.8, 129.1, 128.3, 126.6, 124.1, 119.9 (q, ^1^*J*_C–F_ = 272.2 Hz), 115.4, 75.7, 58.8, 19.6, 13.5 ppm; ^19^F NMR (471 MHz, CDCl_3_): δ −61.9 (s, 3F) ppm. HRMS (ESI): *m/z* calcd. for C_20_H_17_F_3_N_3_O_2_ [M + H]^+^ 388.1267, found 388.1268.

(rel-4*S*,5*R*)-4-(4-Fluorophenyl)-9-methyl-1-phenyl-3-(trifluoromethyl)-7-oxa-1,2,8-triazaspiro[4.4]nona-2,8-dien-6-one (*syn*-**3f**). The synthesis of this compound was conducted using a standardized protocol, followed by purification through column chromatography on silica gel (200–300 mesh) with petroleum ether/ethyl acetate (20:1 *v*/*v*) as the eluent to give 82.9 mg, 53% yield, yellow oil; ^1^H NMR (500 MHz, CDCl_3_): δ 7.33–7.29 (m, 2H, ArH), 7.19–7.16 (m, 2H, ArH), 7.09–7.06 (m, 3H, ArH), 6.99 (d, *J* = 8.0 Hz, 2H, ArH), 4.84 (s, 1H, CH), 2.17 (s, 3H, CH_3_) ppm; ^13^C NMR (126 MHz, CDCl_3_): δ 169.9, 165.5, 163.6 (d, ^1^*J*_C–F_ = 250.9 Hz), 141.1, 138.2 (q, ^2^*J*_C–F_ = 37.9 Hz), 131.3 (d, ^3^*J*_C–F_ = 8.7 Hz), 129.9, 124.2 (d, ^4^*J*_C–F_ = 3.4 Hz), 124.1, 119.9 (q, ^1^*J*_C–F_ = 272.0 Hz), 116.3 (d, ^2^*J*_C–F_ = 22.2 Hz), 114.9, 77.2, 59.7, 11.8 ppm; ^19^F NMR (471 MHz, CDCl_3_): δ −62.3 (s, 3F), −110.2 (m, 1F) ppm. HRMS (ESI): *m/z* calcd. for C_19_H_14_F_4_N_3_O_2_ [M + H]^+^ 392.1017, found 392.0996.

(rel-4*S*,5*R*)-4-(4-Chlorophenyl)-9-methyl-1-phenyl-3-(trifluoromethyl)-7-oxa-1,2,8-triazaspiro[4.4]nona-2,8-dien-6-one (*syn*-**3g**). The synthesis of this compound was conducted using a standardized protocol, followed by purification through column chromatography on silica gel (200–300 mesh) with petroleum ether/ethyl acetate (20:1 *v*/*v*) as the eluent to give 101.1 mg, 62% yield, yellow oil; ^1^H NMR (500 MHz, CDCl_3_): δ 7.37 (d, *J* = 7.5 Hz, 2H, ArH), 7.33–7.30 (m, 2H, ArH), 7.12 (d, *J* = 8.5 Hz, 2H, ArH), 7.09 (t, *J* = 7.5 Hz, 1H, ArH), 6.99 (d, *J* = 8.0 Hz, 2H, ArH), 4.81 (s, 1H, CH), 2.17 (s, 3H, CH_3_) ppm; ^13^C NMR (126 MHz, CDCl_3_): δ 169.8, 165.4, 141.1, 138.0 (q, ^2^*J*_C–F_ = 37.9 Hz), 136.4, 130.7, 129.9, 129.4, 126.9, 124.1, 119.8 (q, ^1^*J*_C–F_ = 272.7 Hz), 115.0, 77.1, 59.7, 11.8 ppm; ^19^F NMR (471 MHz, CDCl_3_): δ −62.1 (s, 3F), ppm. HRMS (ESI): *m/z* calcd. for C_19_H_14_ClF_3_N_3_O_2_ [M + H]^+^408.0721, found 408.0712.

(rel-4*S*,5*R*)-4-(4-Bromophenyl)-9-methyl-1-phenyl-3-(trifluoromethyl)-7-oxa-1,2,8-triazaspiro[4.4]nona-2,8-dien-6-one (*syn*-**3h**). The synthesis of this compound was conducted using a standardized protocol, followed by purification through column chromatography on silica gel (200–300 mesh) with petroleum ether/ethyl acetate (20:1 *v*/*v*) as the eluent to give 108.5 mg, 60% yield, yellow solid; m.p. 233–235 °C; ^1^H NMR (500 MHz, CDCl_3_): δ 7.37 (d, *J* = 8.5 Hz, 2H, ArH), 7.33–7.29 (m, 2H, ArH), 7.12 (d, *J* = 8.5 Hz 2H, ArH), 7.09 (t, *J* = 7.5 Hz, 1H, ArH), 6.99 (d, *J* = 8.0 Hz 2H, ArH), 4.81 (s, 1H, CH), 2.18 (s, 3H, CH_3_) ppm; ^13^C NMR (126 MHz, CDCl_3_): δ 169.8, 165.3, 141.1, 137.9 (q, ^2^*J*_C–F_ = 38.0 Hz), 132.4, 130.9, 129.9, 127.4, 124.6, 124.1, 119.8 (q, ^1^*J*_C–F_ = 272.0 Hz), 116.6, 77.1, 59.8, 11.8 ppm; ^19^F NMR (471 MHz, CDCl_3_): δ −62.3 (s, 3F) ppm. HRMS (ESI): *m/z* calcd. for C_19_H_14_BrF_3_N_3_O_2_ [M + H]^+^ 452.0216, found 452.0209.

(rel-4*S*,5*R*)-4-(3-Chlorophenyl)-9-methyl-1-phenyl-3-(trifluoromethyl)-7-oxa-1,2,8-triazaspiro[4.4]nona-2,8-dien-6-one (*syn*-**3i**). The synthesis of this compound was conducted using a standardized protocol, followed by purification through column chromatography on silica gel (200–300 mesh) with petroleum ether/ethyl acetate (20:1 *v*/*v*) as the eluent to give 44.0 mg, 27% yield, yellow oil; ^1^H NMR (500 MHz, CDCl_3_): δ 7.14 (d, *J* = 8.0 Hz, 2H, ArH), 7.07 (t, *J* = 7.8 Hz, 2H, ArH), 6.94–6.86 (m, 3H, ArH), 6.80 (s, 1H, ArH), 6.74 (d, *J* = 7.5 Hz, 1H, ArH), 6.04 (s, 1H, CH), 2.54 (s, 3H, CH_3_) ppm; ^13^C NMR (126 MHz, CDCl_3_): δ 169.4, 142.4, 140.8 (q, ^2^*J*_C–F_ = 37.1 Hz), 135.6, 133.4, 128.7, 128.5, 128.4, 127.6, 126.9, 125.6, 125.2, 119.7 (q, ^1^*J*_C–F_ = 272.7 Hz), 119.4, 105.0, 77.2, 22.5 ppm; ^19^F NMR (471 MHz, CDCl_3_): δ −70.1 (s, 3F) ppm. HRMS (ESI): *m/z* calcd. for C_19_H_14_ClF_3_N_3_O_2_ [M + H]^+^ 408.0721, found 408.0707.

(rel-4*S*,5*R*)-4-(2-Chlorophenyl)-9-methyl-1-phenyl-3-(trifluoromethyl)-7-oxa-1,2,8-triazaspiro[4.4]nona-2,8-dien-6-one (*syn*-**3j**). The synthesis of this compound was conducted using a standardized protocol, followed by purification through column chromatography on silica gel (200–300 mesh) with petroleum ether/ethyl acetate (20:1 *v*/*v*) as the eluent to give 86.4 mg, 53% yield, yellow oil; ^1^H NMR (500 MHz, CDCl_3_): δ 7.54–7.51 (m, 1H, ArH), 7.42–7.35 (m, 2H, ArH), 7.32–7.28 (m, 3H, ArH), 7.10 (t, *J* = 7.2 Hz, 1H, ArH), 6.99 (d, *J* = 8.5 Hz, 2H, ArH), 5.67 (s, 1H, CH), 1.34 (s, 3H, CH_3_) ppm; ^13^C NMR (126 MHz, CDCl_3_): δ 174.0, 163.7, 141.0, 138.1 (q, ^2^*J*_C–F_ = 38.3 Hz), 134.7, 131.3, 130.8, 130.3, 129.8, 128.6, 127.3, 124.4, 119.8 (q, ^1^*J*_C–F_ = 272.1 Hz), 115.8, 75.6, 58.6, 13.3 ppm; ^19^F NMR (471 MHz, CDCl_3_): δ −61.6 (s, 3F), ppm. HRMS (ESI): *m/z* calcd. for C_19_H_14_ClF_3_N_3_O_2_ [M + H]^+^ 408.0721, found 408.0722.

(rel-4*S*,5*R*)-9-Methyl-1-phenyl-4-(thiophen-3-yl)-3-(trifluoromethyl)-7-oxa-1,2,8-triazaspiro[4.4]nona-2,8-dien-6-one (*syn*-**3k**). The synthesis of this compound was conducted using a standardized protocol, followed by purification through column chromatography on silica gel (200–300 mesh) with petroleum ether/ethyl acetate (20:1 *v*/*v*) as the eluent to give 85.0 mg, 56% yield, yellow oil; ^1^H NMR (500 MHz, CDCl_3_): δ 7.36 (d, *J* = 5.0 Hz, 1H, ArH), 7.30 (d, *J* = 8.0 Hz, 2H, ArH), 7.09–7.04 (m, 3H, ArH), 7.00 (d, *J* = 8.0 Hz, 2H, ArH), 5.17 (s, 1H, CH), 2.18 (s, 3H, CH_3_) ppm; ^13^C NMR (126 MHz, CDCl_3_): δ 169.6, 165.2, 141.3, 137.5 (q, ^2^*J*_C–F_ = 38.1 Hz), 130.3, 129.9, 128.5, 128.2, 127.8, 124.2, 119.7 (q, ^1^*J*_C–F_ = 272.0 Hz), 115.2, 77.8, 54.6, 11.8 ppm; ^19^F NMR (471 MHz, CDCl_3_): δ −62.2 (s, 3F) ppm. HRMS (ESI): *m/z* calcd. for C_17_H_13_F_3_N_3_O_2_S [M + H]^+^ 380.0675, found 380.0663.

(rel-4*S*,5*R*)-9-Methyl-4-(naphthalen-1-yl)-1-phenyl-3-(trifluoromethyl)-7-oxa-1,2,8-triazaspiro[4.4]nona-2,8-dien-6-one (*syn*-**3l**). The synthesis of this compound was conducted using a standardized protocol, followed by purification through column chromatography on silica gel (200–300 mesh) with petroleum ether/ethyl acetate (20:1 *v*/*v*) as the eluent to give 133.8 mg, 79% yield, yellow solid; m.p. 197–199 °C; ^1^H NMR (500 MHz, CDCl_3_): δ 7.93–7.90 (m, 2H, ArH), 7.64 (d, *J* = 8.0 Hz, 1H, ArH), 7.60–7.51 (m, 3H, ArH), 7.41 (d, *J* = 7.5 Hz, 1H, ArH), 7.30 (t, *J* = 8.0 Hz, 2H, ArH), 7.07 (t, *J* = 7.5 Hz, 1H, ArH), 7.00 (d, *J* = 8.0 Hz, 2H, ArH), 5.71 (s, 1H, CH), 2.26 (s, 3H, CH_3_) ppm; ^13^C NMR (126 MHz, CDCl_3_): δ 169.8, 165.1, 141.1, 138.9 (q, ^2^*J*_C–F_ = 38.0 Hz), 134.1, 130.8, 130.6, 129.9, 129.7, 129.1, 127.6, 126.3, 125.2, 124.1, 123.9, 121.0, 120.0 (q, ^1^*J*_C–F_ = 272.1 Hz), 114.9, 75.9, 55.0, 11.9 ppm. ^19^F NMR (471 MHz, CDCl_3_): δ −61.9 (s, 3F) ppm. HRMS (ESI): *m/z* calcd. for C_23_H_17_F_3_N_3_O_2_ [M + H]^+^424.1267, found 424.1269.

(rel-4*R*,5*R*)-9-Methyl-4-(naphthalen-1-yl)-1-phenyl-3-(trifluoromethyl)-7-oxa-1,2,8-triazaspiro[4.4]nona-2,8-dien-6-one (*anti*-**3l**). The synthesis of this compound was conducted using a standardized protocol, followed by purification through column chromatography on silica gel (200–300 mesh) with petroleum ether/ethyl acetate (20:1 *v*/*v*) as the eluent to give 23.7 mg, 14% yield, colorless oil; ^1^H NMR (500 MHz, CDCl_3_): δ 7.94 (t, *J* = 7.5 Hz, 2H, ArH), 7.77 (d, *J* = 8.5 Hz, 1H, ArH), 7.66–7.58 (m, 2H, ArH), 7.51 (t, *J* = 7.8 Hz, 1H, ArH), 7.43 (d, *J* = 7.0 Hz, 2H, ArH), 7.32–7.28 (m, 2H, ArH), 7.08 (t, *J* = 7.5 Hz, 1H, ArH), 7.00 (d, *J* = 8.0 Hz, 1H, ArH), 5.97 (s, 1H, CH), 1.12 (s, 3H, CH_3_) ppm; ^13^C NMR (126 MHz, CDCl_3_): δ 174.7, 164.8, 141.3, 139.2 (q, ^2^*J*_C–F_ = 38.0 Hz), 134.1, 131.0, 130.9, 129.8, 129.4, 128.1, 127.5, 127.0, 125.9, 124.7, 124.2, 121.8, 120.0 (q, ^1^*J*_C–F_ = 272.2 Hz), 115.6, 76.2, 57.9, 13.5 ppm. ^19^F NMR (471 MHz, CDCl_3_): δ −61.4 (s, 3F), ppm. HRMS (ESI): *m/z* calcd. for C_23_H_17_F_3_N_3_O_2_ [M + H]^+^ 424.1267, found 424.1272.

(rel-4*S*,5*R*)-9-Mmethyl-4-phenyl-1-(*p*-tolyl)-3-(trifluoromethyl)-7-oxa-1,2,8-triazaspiro[4.4]nona-2,8-dien-6-one (*syn*-**3m**). The synthesis of this compound was conducted using a standardized protocol, followed by purification through column chromatography on silica gel (200–300 mesh) with petroleum ether/ethyl acetate (20:1 *v*/*v*) as the eluent to give 108.5 mg, 70% yield, white solid; m.p. 195–197 °C; ^1^H NMR (400 MHz, CDCl_3_): δ 7.42–7.38 (m, 3H, ArH), 7.20–7.17 (m, 2H, ArH), 7.11 (d, *J* = 8.0 Hz, 2H, ArH), 6.90 (d, *J* = 8.8 Hz, 2H, ArH), 4.84 (s, 1H, CH), 2.29 (s, 3H, CH_3_), 2.20 (s, 3H, CH_3_) ppm; ^13^C NMR (101 MHz, CDCl_3_): δ 169.9, 165.5, 139.0, 138.1 (q, ^2^*J*_C–F_ = 37.8 Hz), 133.8, 130.3, 130.0, 129.4, 129.1, 128.5, 120.0 (q, ^1^*J*_C–F_ = 272.3 Hz), 115.9, 77.7, 60.4, 20.6, 11.8 ppm; ^19^F NMR (376 MHz, CDCl_3_): δ −62.2 (s, 3F) ppm. HRMS (ESI): *m/z* calcd. for C_20_H_17_F_3_N_3_O_2_ [M + H]^+^ 388.1267, found 388.1254.

(rel-4*R*,5*R*)-9-Methyl-4-phenyl-1-(*p*-tolyl)-3-(trifluoromethyl)-7-oxa-1,2,8-triazaspiro[4.4]nona-2,8-dien-6-one (*anti*-**3m**). The synthesis of this compound was conducted using a standardized protocol, followed by purification through column chromatography on silica gel (200–300 mesh) with petroleum ether/ethyl acetate (20:1 *v*/*v*) as the eluent to give 20.1 mg, 13% yield, yellow oil; ^1^H NMR (400 MHz, CDCl_3_): δ 7.44–7.42 (m, 3H, ArH), 7.23–7.21 (m, 2H, ArH), 7.10 (d, *J* = 8.0 Hz, 2H, ArH), 6.89 (d, *J* = 8.8 Hz, 2H, ArH), 5.14 (s, 1H, CH), 2.29 (s, 3H, CH_3_), 1.37 (s, 3H, CH_3_) ppm; ^13^C NMR (101MHz, CDCl_3_): δ 174.5, 164.5, 138.9,138.4 (q, ^2^*J*_C–F_ = 38.1 Hz), 134.2, 130.3, 130.0, 129.7, 129.6, 128.7, 120.0 (q, ^1^*J*_C–F_ = 272.4 Hz), 115.8, 77.6, 62.6, 20.6, 13.5 ppm; ^19^F NMR (376 MHz, CDCl_3_): δ −61.8 (s, 3F) ppm. HRMS (ESI): *m/z* calcd. for C_20_H_17_F_3_N_3_O_2_ [M + H]^+^ 388.1267, found 388.1256.

(rel-4*S*,5*R*)-4-(4-Bromophenyl)-9-methyl-1-(*p*-tolyl)-3-(trifluoromethyl)-7-oxa-1,2,8-triazaspiro[4.4]nona-2,8-dien-6-one (*syn*-**3n**). The synthesis of this compound was conducted using a standardized protocol, followed by purification through column chromatography on silica gel (200–300 mesh) with petroleum ether/ethyl acetate (20:1 *v*/*v*) as the eluent to give 102.6 mg, 55% yield, yellow oil; ^1^H NMR (500 MHz, CDCl_3_): δ 7.52 (d, *J* = 8.0 Hz, 2H, ArH), 7.11 (d, *J* = 8.0 Hz, 2H, ArH), 7.06 (d, *J* = 8.0 Hz, 2H, ArH), 6.89 (d, *J* = 8.5 Hz, 2H, ArH), 4.78 (s, 1H, CH), 2.29 (s, 3H, CH_3_), 2.18 (s, 3H, CH_3_) ppm; ^13^C NMR (126 MHz, CDCl_3_): δ 169.9, 165.3, 138.8, 137.6 (q, ^2^*J*_C–F_ = 37.8 Hz), 134.1, 132.4, 130.9, 130.4, 127.5, 124.5, 119.9 (q, ^1^*J*_C–F_ = 272.0 Hz), 115.4, 77.5, 59.7, 20.6, 11.8 ppm; ^19^F NMR (471 MHz, CDCl_3_): δ −62.2 (s, 3F) ppm. HRMS (ESI): *m/z* calcd. for C_20_H_16_BrF_3_N_3_O_2_ [M + H]^+^ 466.0373, found 466.0316.

(rel-4*S*,5*R*)-9-Methyl-4-(*o*-tolyl)-1-(*p*-tolyl)-3-(trifluoromethyl)-7-oxa-1,2,8-triazaspiro[4.4]nona-2,8-dien-6-one (*syn*-**3o**). The synthesis of this compound was conducted using a standardized protocol, followed by purification through column chromatography on silica gel (200–300 mesh) with petroleum ether/ethyl acetate (20:1 *v*/*v*) as the eluent to give 72.3 mg, 45% yield, yellow oil; ^1^H NMR (500 MHz, CDCl_3_): δ 7.29–7.23 (m, 2H, ArH), 7.21 (d, *J* = 8.0 Hz, 1H, ArH), 7.16 (d, *J* = 7.5 Hz, 1H, ArH), 7.10 (d, *J* = 8.5 Hz, 2H, ArH), 6.89 (d, *J* = 8.5 Hz, 2H, ArH), 5.14 (s, 1H, CH), 2.29 (s, 3H, CH_3_), 2.28 (s, 3H, CH_3_), 2.16 (s, 3H, CH_3_) ppm; ^13^C NMR (126 MHz, CDCl_3_): δ 170.0, 165.7, 138.9, 138.8 (q, ^2^*J*_C–F_ = 37.6 Hz), 135.6, 133.7, 131.1, 130.5, 130.3, 129.7, 127.0, 126.5, 119.9 (q, ^1^*J*_C–F_ = 271.9 Hz), 115.1, 76.2, 56.0, 20.6, 19.5, 11.8 ppm; ^19^F NMR (471 MHz, CDCl_3_): δ −62.2 (s, 3F) ppm. HRMS (ESI): *m/z* calcd. for C_21_H_19_F_3_N_3_O_2_ [M + H]^+^ 402.1424, found 402.1429.

(rel-4*R*,5*R*)-9-Mmethyl-4-(*o*-tolyl)-1-(*p*-tolyl)-3-(trifluoromethyl)-7-oxa-1,2,8-triazaspiro[4.4]nona-2,8-dien-6-one (*anti*-**3o**). The synthesis of this compound was conducted using a standardized protocol, followed by purification through column chromatography on silica gel (200–300 mesh) with petroleum ether/ethyl acetate (20:1 *v*/*v*) as the eluent to give 11.2 mg, 7% yield, yellow oil; ^1^H NMR (500 MHz, CDCl_3_): δ 7.34–7.28 (m, 2H, ArH), 7.27–7.25 (m, 1H, ArH), 7.17 (d, *J* = 7.5 Hz, 1H, ArH), 7.10 (d, *J* = 9.0 Hz, 2H, ArH), 6.88 (d, *J* = 8.5 Hz, 2H, ArH), 5.34 (s, 1H, CH), 2.30 (s, 3H, CH_3_), 2.29 (s, 3H, CH_3_), 1.31 (s, 3H, CH_3_) ppm; ^13^C NMR (126 MHz, CDCl_3_): δ 174.4, 165.6, 139.3 (q, ^2^*J*_C–F_ = 38.0 Hz), 139.1, 136.8, 134.2, 131.8, 130.3, 129.9, 129.1, 128.5, 126.6, 119.9 (q, ^1^*J*_C–F_ = 272.4 Hz), 116.1, 76.1, 58.6, 20.6, 19.6, 13.5 ppm; ^19^F NMR (471 MHz, CDCl_3_): δ −61.8 (s, 3F), ppm. HRMS (ESI): *m/z* calcd. for C_21_H_19_F_3_N_3_O_2_ [M + H]^+^ 402.1424, found 402.1429.

(rel-4*S*,5*R*)-9-Methyl-4-(*m*-tolyl)-1-(*p*-tolyl)-3-(trifluoromethyl)-7-oxa-1,2,8-triazaspiro[4.4]nona-2,8-dien-6-one (*syn*-**3p**). The synthesis of this compound was conducted using a standardized protocol, followed by purification through column chromatography on silica gel (200–300 mesh) with petroleum ether/ethyl acetate (20:1 *v*/*v*) as the eluent to give 93.1 mg, 58% yield, yellow oil; ^1^H NMR (500 MHz, CDCl_3_): δ 7.27 (t, *J* = 7.8 Hz, 1H, ArH), 7.20 (d, *J* = 7.5 Hz, 1H, ArH), 7.10 (d, *J* = 8.5 Hz, 2H, ArH), 6.98 (d, *J* = 9.5 Hz, 2H, ArH), 6.90 (d, *J* = 8.5 Hz, 2H, ArH), 4.80 (s, 1H, CH), 2.34 (s, 3H, CH_3_), 2.28 (s, 3H, CH_3_), 2.19 (s, 3H, CH_3_) ppm; ^13^C NMR (126 MHz, CDCl_3_): δ 169.9, 165.5, 139.1, 138.9, 138.2 (q, ^2^*J*_C–F_ =37.8 Hz), 133.7, 130.8, 130.3, 129.9, 128.9, 128.4, 126.5, 120.0 (q, ^1^*J*_C–F_ = 271.8 Hz), 115.3, 77.7, 60.5, 21.3, 20.6, 11.8 ppm; ^19^F NMR (471 MHz, CDCl_3_): δ −62.2 (s, 3F) ppm. HRMS (ESI): *m/z* calcd. for C_21_H_19_F_3_N_3_O_2_ [M + H]^+^ 402.1424, found 402.1420.

(rel-4*S*,5*R*)-1-(4-Fluorophenyl)-9-methyl-4-phenyl-3-(trifluoromethyl)-7-oxa-1,2,8-triazaspiro[4.4]nona-2,8-dien-6-one (*syn*-**3q**). The synthesis of this compound was conducted using a standardized protocol, followed by purification through column chromatography on silica gel (200–300 mesh) with petroleum ether/ethyl acetate (20:1 *v*/*v*) as the eluent to give 112.7 mg, 72% yield, yellow oil; ^1^H NMR (400 MHz, CDCl_3_): δ 7.42–7.36 (m, 3H, ArH), 7.19–7.17 (m, 2H, ArH), 7.04–6.96 (m, 4H, ArH), 4.87 (s, 1H, CH), 2.21 (s, 3H, CH_3_) ppm; ^13^C NMR (101 MHz, CDCl_3_): δ 169.8, 165.2, 159.4 (d, ^1^*J* = 245.1 Hz), 138.9 (q, ^2^*J* = 38.0 Hz), 137.6 (d, ^4^*J* = 2.4 Hz), 130.1, 129.3, 129.1, 128.2, 119.8 (q, ^1^*J* = 272.6 Hz), 117.2 (d, ^3^*J* = 8.1 Hz), 116.7 (d, ^2^*J* = 22.9 Hz), 78.0, 60.6, 11.8 ppm; ^19^F NMR (376 MHz, CDCl_3_): δ −62.3 (s, 3F), 118.4 (m, 1F) ppm. HRMS (ESI): *m/z* calcd. for C_19_H_14_F_4_N_3_O_2_ [M + H]^+^ 392.1017, found 392.0983.

(rel-4*R*,5*R*)-1-(4-Fluorophenyl)-9-methyl-4-phenyl-3-(trifluoromethyl)-7-oxa-1,2,8-triazaspiro[4.4]nona-2,8-dien-6-one (*anti*-**3q**). The synthesis of this compound was conducted using a standardized protocol, followed by purification through column chromatography on silica gel (200–300 mesh) with petroleum ether/ethyl acetate (20:1 *v*/*v*) as the eluent to give 21.9 mg, 14% yield, yellow oil; ^1^H NMR (400 MHz, CDCl_3_): δ 7.46–7.44 (m, 3H, ArH), 7.23–7.21 (m, 2H, ArH), 7.04–6.96 (m, 4H, ArH), 5.15 (s,1H, CH), 1.39 (s, 3H, CH_3_) ppm; ^13^C NMR (101 MHz, CDCl_3_): δ 174.2, 164.3, 159.7 (d, ^1^*J* = 245.5 Hz), 139.4 (q, ^2^*J*_C-F_ = 38.3 Hz), 137.5 (d, ^4^*J* = 2.7 Hz), 130.1, 129.7, 129.5, 128.7, 119.8 (q, ^1^*J*_C-F_ = 272.7 Hz), 117.9 (d, ^3^*J* = 8.2 Hz), 116.6 (d, ^2^*J* = 23.0 Hz), 77.6, 62.6, 13.5 ppm; ^19^F NMR (376 MHz, CDCl_3_): δ −62.0 (s, 3F), 117.9 (m, 1F) ppm. HRMS (ESI): *m/z* calcd. for C_19_H_14_F_4_N_3_O_2_ [M + H]^+^ 392.1017, found 392.1004.

(rel-4*S*,5*R*)-1-(4-Fluorophenyl)-9-methyl-4-(*p*-tolyl)-3-(trifluoromethyl)-7-oxa-1,2,8-triazaspiro[4.4]nona-2,8-dien-6-one (*syn*-**3r**). The synthesis of this compound was conducted using a standardized protocol, followed by purification through column chromatography on silica gel (200–300 mesh) with petroleum ether/ethyl acetate (20:1 *v*/*v*) as the eluent to give 123.2 mg, 76% yield, yellow oil; ^1^H NMR (500 MHz, CDCl_3_): δ 7.19 (d, *J* = 8.0 Hz, 2H, ArH), 7.06 (d, *J* = 8.0 Hz, 2H, ArH), 7.03–6.96 (m, 4H, ArH), 4.85 (s, 1H, CH), 2.34 (s, 3H, CH_3_), 2.21 (s, 3H, CH_3_) ppm; ^13^C NMR (126 MHz, CDCl_3_): δ 169.8, 165.2, 159.4 (d, ^1^*J* = 244.9 Hz), 140.2, 139.1 (q, ^2^*J* = 37.8 Hz), 137.7 (^4^d, *J* = 2.5 Hz), 129.9, 129.2, 125.1, 119.8 (q, ^1^*J* = 272.2 Hz), 117.1 (d, ^3^*J* = 7.3 Hz), 116.6 (d, ^2^*J* = 22.9 Hz), 78.1, 60.4, 21.2, 11.8 ppm; ^19^F NMR (471 MHz, CDCl_3_): δ −62.3 (s, 3F), 118.6 (m, 1F) ppm. HRMS (ESI): *m/z* calcd. for C_20_H_16_F_4_N_3_O_2_ [M + H]^+^ 406.1173, found 406.1178.

(rel-4*R*,5*R*)-1-(4-Fluorophenyl)-9-methyl-4-(*p*-tolyl)-3-(trifluoromethyl)-7-oxa-1,2,8-triazaspiro[4.4]nona-2,8-dien-6-one (*anti*-**3r**). The synthesis of this compound was conducted using a standardized protocol, followed by purification through column chromatography on silica gel (200–300 mesh) with petroleum ether/ethyl acetate (20:1 *v*/*v*) as the eluent to give 19.5 mg, 12% yield, yellow oil; ^1^H NMR (500 MHz, CDCl_3_): δ 7.24 (d, *J* = 8.0 Hz, 2H, ArH), 7.09 (d, *J* = 8.0 Hz, 2H, ArH), 7.04–6.95 (m, 4H, ArH), 5.12 (s, 1H, CH), 2.38 (s, 3H, CH_3_), 1.42 (s, 3H, CH_3_) ppm; ^13^C NMR (126 MHz, CDCl_3_): δ 174.3, 164.4, 159.6 (d, ^1^*J* = 244.9 Hz), 140.3, 139.6 (q, ^2^*J* = 37.9 Hz), 137.6 (d, ^4^*J* = 2.6 Hz), 130.3, 128.6, 126.4, 119.9 (q, ^1^*J* = 272.3 Hz), 117.7 (d, ^3^*J* = 8.1 Hz), 116.6 (d, ^2^*J* = 23.1 Hz), 77.7, 62.5, 21.2, 13.6 ppm; ^19^F NMR (471 MHz, CDCl_3_): δ −62.0 (s, 3F), 118.1 (m, 1F) ppm. HRMS (ESI): *m/z* calcd. for C_20_H_16_F_4_N_3_O_2_ [M + H]^+^ 406.1173, found 406.1181.

(rel-4*S*,5*R*)-1-(4-Fluorophenyl)-9-methyl-4-(*o*-tolyl)-3-(trifluoromethyl)-7-oxa-1,2,8-triazaspiro[4.4]nona-2,8-dien-6-one (*syn*-**3s**). The synthesis of this compound was conducted using a standardized protocol, followed by purification through column chromatography on silica gel (200–300 mesh) with petroleum ether/ethyl acetate (20:1 *v*/*v*) as the eluent to give 77.8 mg, 48% yield, yellow oil; ^1^H NMR (500 MHz, CDCl_3_): δ 7.30–7.25 (m, 2H, ArH), 7.24–7.21 (m, 1H, ArH), 7.15 (d, *J* = 7.5 Hz, 1H, ArH), 7.03–6.95 (m, 4H, ArH), 5.16 (s,1H, CH), 2.29 (s, 3H, CH_3_), 2.17 (s, 3H, CH_3_) ppm; ^13^C NMR (126 MHz, CDCl_3_): δ 169.8, 165.4, 159.3 (d, ^1^*J* = 244.4 Hz), 139.6 (q, ^2^*J* = 38.0 Hz), 137.5 (d, ^4^*J* = 2.5 Hz), 135.6, 131.2, 130.5, 129.8, 126.8, 126.6, 119.8 (q, ^1^*J* = 272.2), 116.9 (d, ^3^*J* = 8.1 Hz), 116.7 (d, ^2^*J* = 23.2 Hz), 76.3, 56.2, 19.5, 11.8 ppm; ^19^F NMR (471 MHz, CDCl_3_): δ −62.7 (s, 3F), 118.7 (m, 1F) ppm. HRMS (ESI): *m/z* calcd. for C_20_H_16_F_4_N_3_O_2_ [M + H]^+^ 406.1173, found 406.1170.

(rel-4*R*,5*R*)-1-(4-Fluorophenyl)-9-methyl-4-(*o*-tolyl)-3-(trifluoromethyl)-7-oxa-1,2,8-triazaspiro[4.4]nona-2,8-dien-6-one (*anti*-**3s**). The synthesis of this compound was conducted using a standardized protocol, followed by purification through column chromatography on silica gel (200–300 mesh) with petroleum ether/ethyl acetate (20:1 *v*/*v*) as the eluent to give 9.7 mg, 6% yield, yellow oil; ^1^H NMR (500 MHz, CDCl_3_): δ 7.35–7.27 (m, 3H, ArH), 7.17 (d, *J* = 7.5 Hz, 1H, ArH), 7.04–6.95 (m, 4H, ArH), 5.36 (s, 1H, CH), 2.30 (s, 3H, CH_3_), 1.32 (s, 3H, CH_3_) ppm; ^13^C NMR (126 MHz, CDCl_3_): δ 174.1, 165.3, 159.7 (d, ^1^*J* = 245.1 Hz), 140.4 (q, ^2^*J* = 38.1 Hz), 137.7 (d, ^4^*J* = 2.6 Hz), 136.8, 131.9, 130.1, 129.0, 128.2, 126.7, 119.8 (q, ^1^*J* = 272.2 Hz), 118.1 (d, ^3^*J* = 8.2 Hz), 116.7 (d, ^2^*J* = 23.1 Hz), 76.3, 58.7, 19.6, 13.5 ppm; ^19^F NMR (471 MHz, CDCl_3_): δ −62.1 (s, 3F), 117.9 (m, 1F) ppm. HRMS (ESI): *m/z* calcd. for C_20_H_16_F_4_N_3_O_2_ [M + H]^+^ 406.1173, found 406.1170.

### 3.4. Procedure for the Scaled-Up Synthesis of Compound ***3a***

To a dried 50 mL round-bottom flask, trifluoromethyl bromohydrazone **1a** (4,8 mmol, 1.28 g), unsaturated isoxazolone derivative **2a** (4 mmol, 0.749 g), K_2_CO_3_ (4 mmol, 0.553 g) and 1,2-dichloroethane (20 mL) were added. The reaction mixture was stirred at room temperature for 24 h. Upon completion, the solvent was removed under reduced pressure, and the residue was purified by flash chromatography on silica gel (eluent: petroleum ether/ethyl acetate = 20:1, *v*/*v*) to afford product **3a** in 83% overall yield with 3.8:1 dr.

## 4. Conclusions

In summary, a [3+2] cycloaddition reaction between nitrile imines and unsaturated isoxazolones was developed. This protocol employs inexpensive, readily available, and environmentally benign K_2_CO_3_ as the base, affording a series of trifluoromethylated spiroisoxazolone derivatives in moderate to excellent yields. Furthermore, the reaction exhibits a broad substrate scope, as both the trifluoroacetyl bromohydrazones and unsaturated isoxazolones can be widely varied. This versatility enables the synthesis of products bearing diverse potential pharmacophores, thereby providing a valuable library of candidate compounds for new drug discovery.

## Figures and Tables

**Table 1:** Optimization of reaction conditions *^a^*.

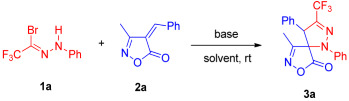					
Entry	1a/2a/Base (Mole Ratio)	Solvent	Base	Total Yield (%)	dr *^b^*
1	1:1:1	THF	K_2_CO_3_	52	1.7:1
2	1:1:1	CH_2_Cl_2_	K_2_CO_3_	66	3.9:1
3	1:1:1	DCE	K_2_CO_3_	78	4.1:1
4	1:1:1	CHCl_3_	K_2_CO_3_	45	3.1:1
5	1:1:1	EtOH	K_2_CO_3_	60	2.6:1
6	1:1:1	DCE	Cs_2_CO_3_	32	1.3:1
7	1:1:1	DCE	Et_3_N	40	1.7:1
8	1:1:1	DCE	DIPEA	54	2.9:1
9	1:1:1	DCE	DABCO	25	2.1:1
10	1.2:1:1.2	DCE	K_2_CO_3_	92	4.2:1
11	1.5:1:1.5	DCE	K_2_CO_3_	83	4.4:1
12 *^c^*	1.2:1:1.2	DCE	K_2_CO_3_	80	3.7:1
13 *^d^*	1.2:1:1.2	DCE	K_2_CO_3_	77	4.6:1

*^a^* Unless otherwise specified, reactions were carried out with **1a** (0.48 mmol), **2a** (0.40 mmol) and base (0.48 mmol) in solvent (2.0 mL) at room temperature for 24 h. *^b^* Diastereomeric ratio was determined by ^1^H NMR analysis of the crude reaction mixture. *^c^* The reaction was conducted at 50 °C. *^d^* The reaction was conducted at 0 °C.

## Data Availability

Data are contained within this article and Supplementary Materials.

## Supplementary Materials

The following supporting information can be downloaded at: https://www.mdpi.com/article/10.3390/molecules31010073/s1, Copies of ^1^H and ^13^C NMR spectra of new compounds.
