# Supplementary material for: Synthesis of Trifluoromethylated Spiroisoxazolones via a [3+2] Cycloaddition of Nitrile Imines and Unsaturated Isoxazolones
Source: Molecules. 2025 Dec 24;31(1):73. doi: 10.3390/molecules31010073 (PMC12786404; doi:10.3390/molecules31010073)
Supplement: Supplementary file 1 [file molecules-31-00073-s001.zip › molecules-4022635-supplementary.pdf]

Synthesis of Trifluoromethylated Spiroisoxazolones via a [3+2] Cycloaddition of Nitrile Imines and Unsaturated Isoxazolones

Wei Zhang[a,b] and Da-Ming Du[a,b*]

[a] *School of Chemistry and Chemical Engineering, Beijing Institute of Technology, Beijing 100081, China*

[b] *Key Laboratory of Medicinal Molecule Science and Pharmaceutical Technology, Ministry of Industry and Information Technology, Beijing 100081, China*

E-mail: dudm@bit.edu.cn

Supporting Information

Contents

1. Copies of ^{1}H and ^{13}C NMR spectra of new compounds……………………..................................S1-S60

1. Copies of ^{1}H and ^{13}C NMR spectra of new compounds

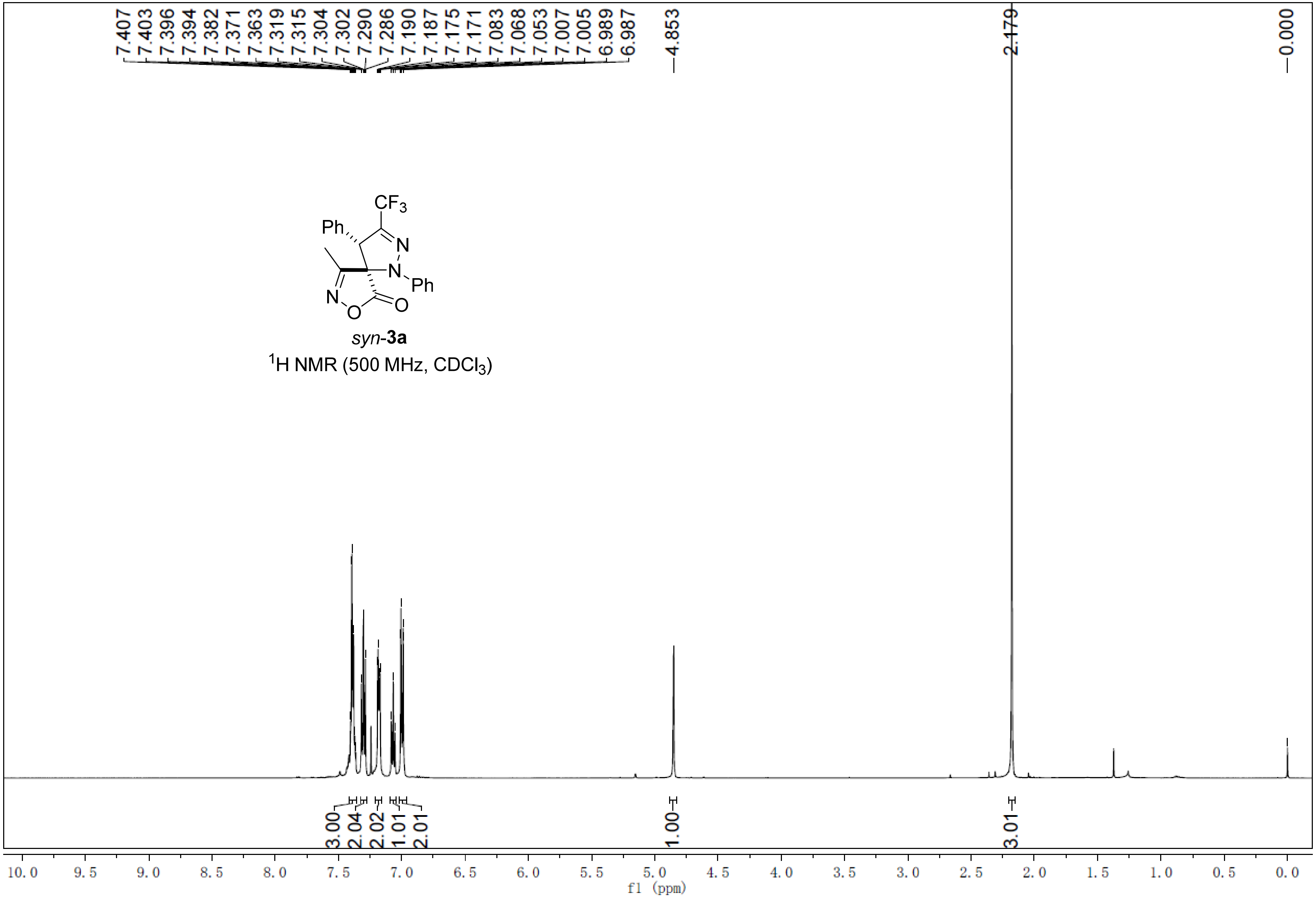

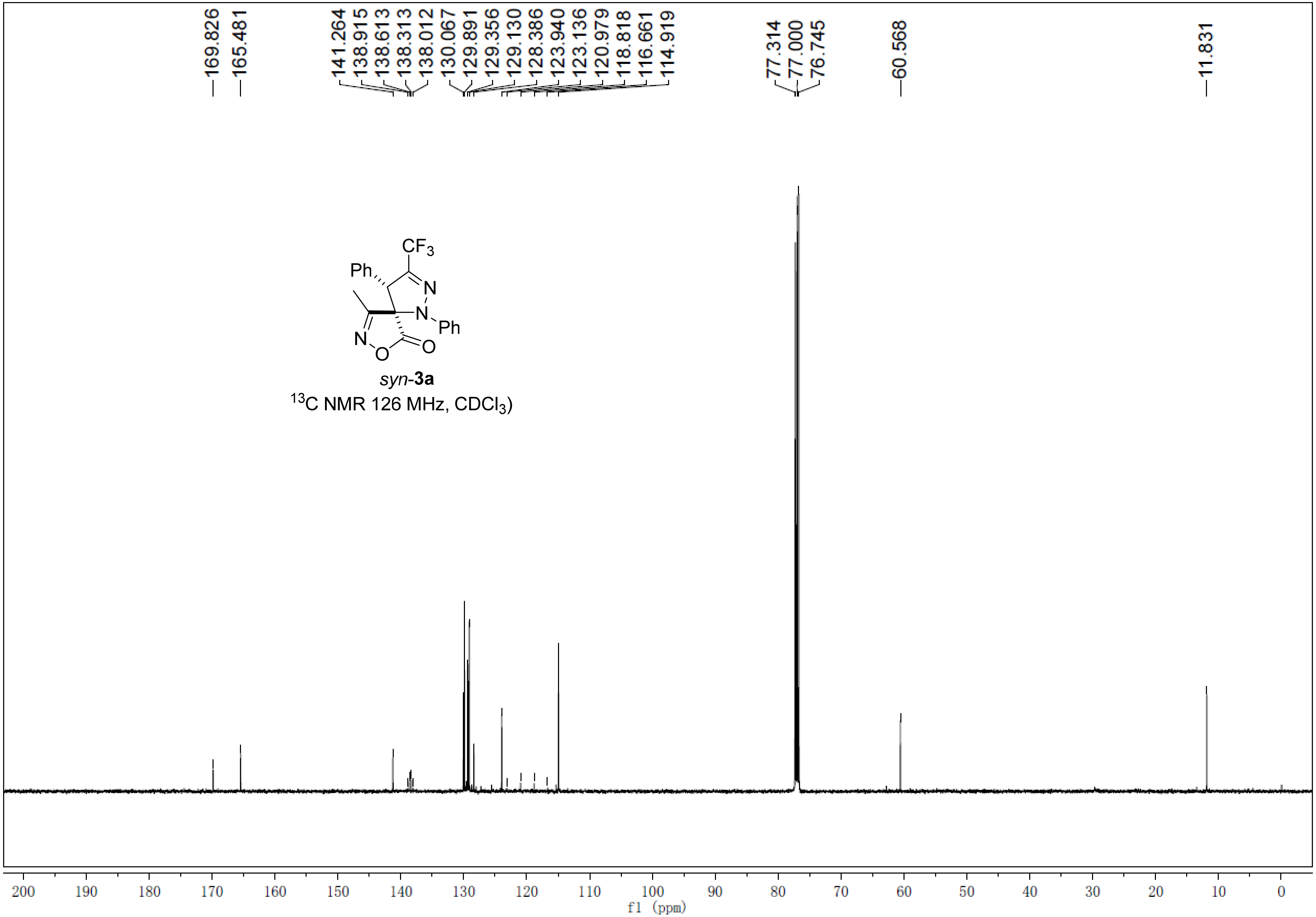
169.826
165.481
141.264
138.915
138.613
138.313
138.012
130.067
129.891
129.356
129.130
128.386
123.940
123.136
120.979
118.818
116.661
114.919
77.314
77.000
76.745
60.568
11.831
CF3
Ph
N
N
Ph
N
O
O
syn-3a
13C NMR 126 MHz, CDCl3)
200
190
180
170
160
150
140
130
120
110
100
90
80
70
60
50
40
30
20
10
0
f1 (ppm)

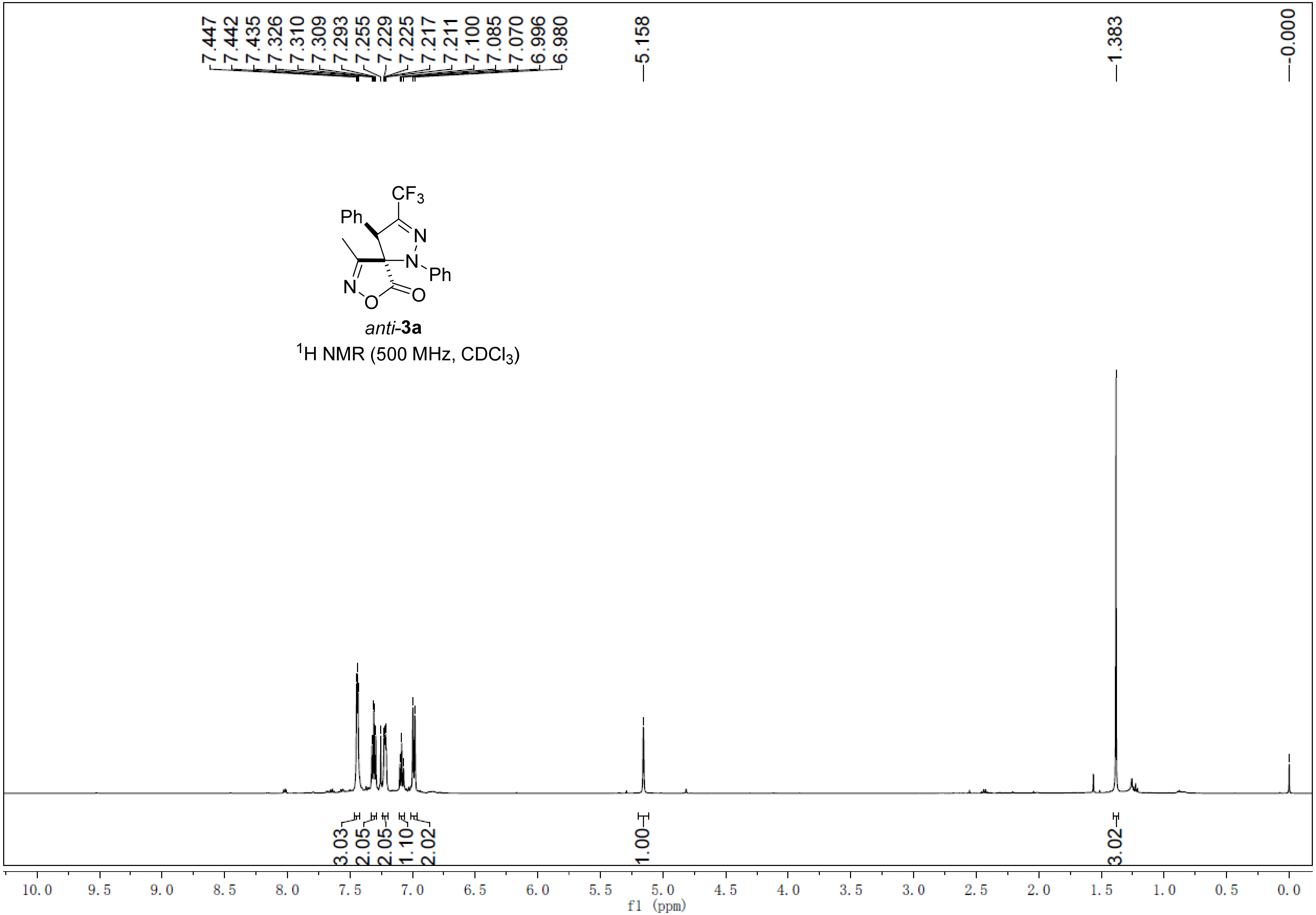

anti-**3a**

^{1}H NMR (500 MHz, $CDCl_3$)

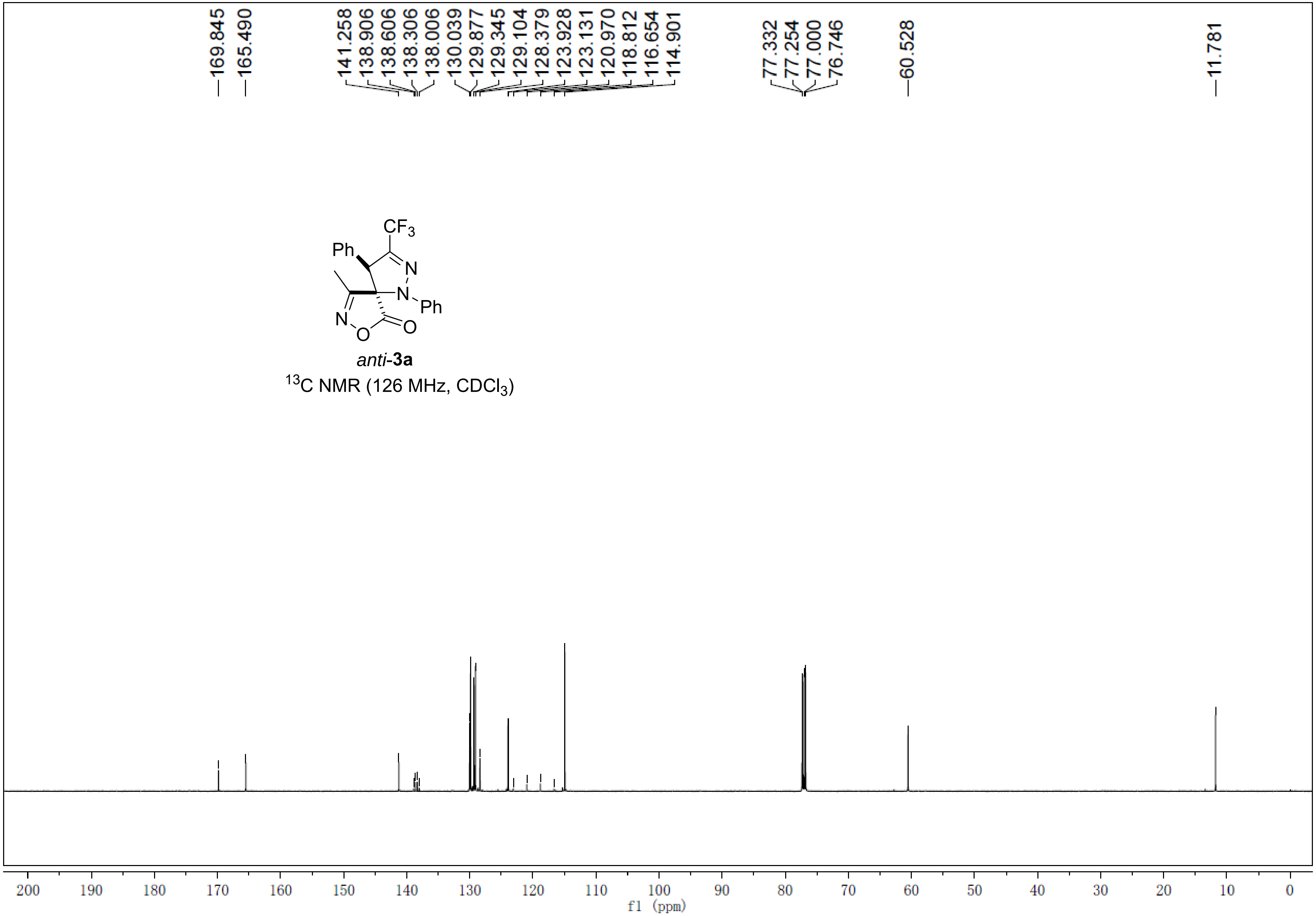
169.845
165.490
141.258
138.906
138.606
138.306
138.006
130.039
129.877
129.345
129.104
128.379
123.928
123.131
120.970
118.812
116.654
114.901
77.332
77.254
77.000
76.746
60.528
11.781
CF3
Ph
N
N
Ph
N
O
O
anti-3a
13C NMR (126 MHz, CDCl3)
200
190
180
170
160
150
140
130
120
110
100
90
80
70
60
50
40
30
20
10
0
f1 (ppm)

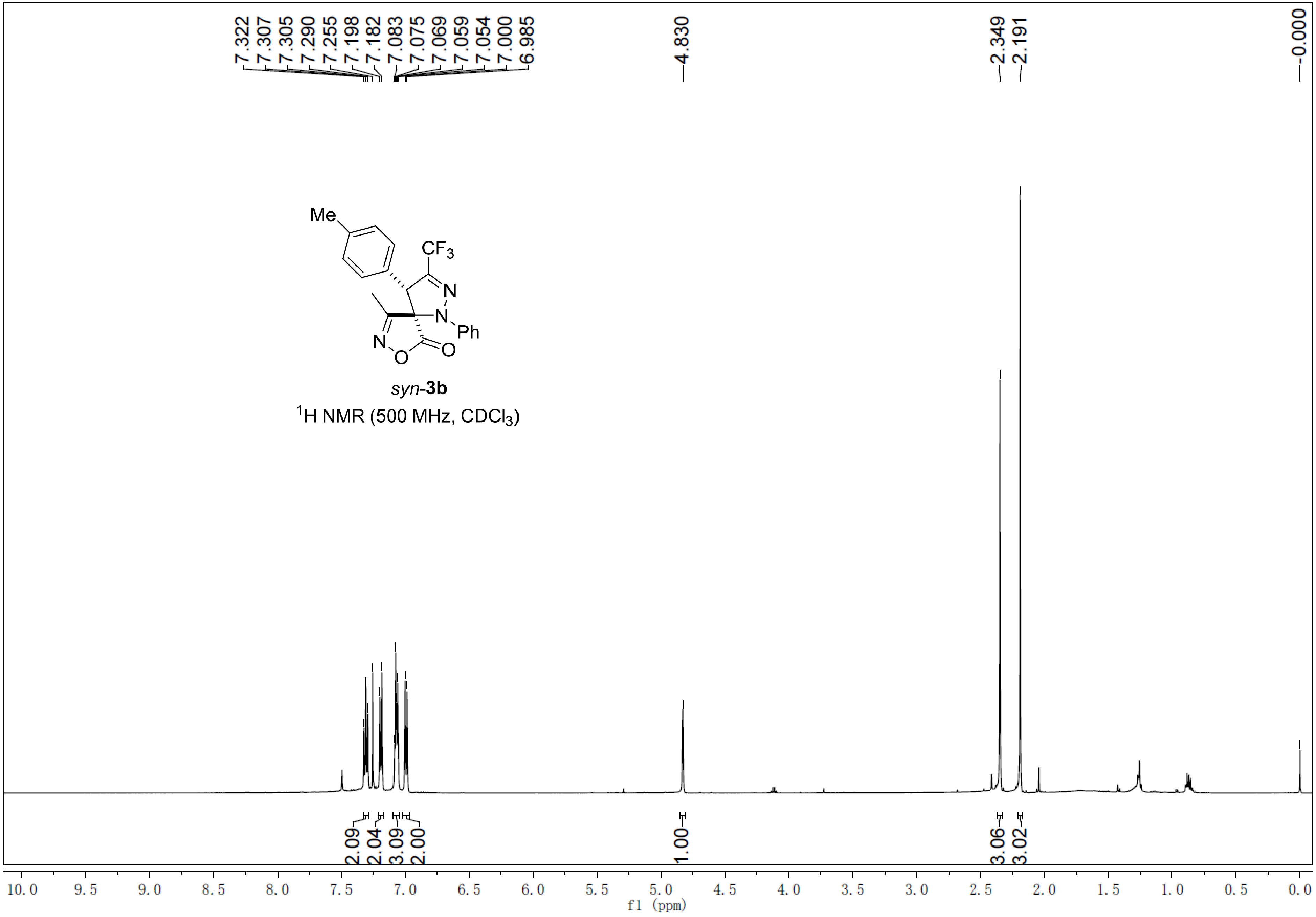
7.322
7.307
7.305
7.290
7.255
7.198
7.182
7.083
7.075
7.069
7.059
7.054
7.000
6.985
4.830
2.349
2.191
-0.000
Me
CF3
N
N
Ph
N
O
O
syn-3b
1H NMR (500 MHz, CDCl3)
2.09
2.04
3.09
2.00
1.00
3.06
3.02
10.0
9.5
9.0
8.5
8.0
7.5
7.0
6.5
6.0
5.5
5.0
4.5
4.0
3.5
3.0
2.5
2.0
1.5
1.0
0.5
0.0
f1 (ppm)

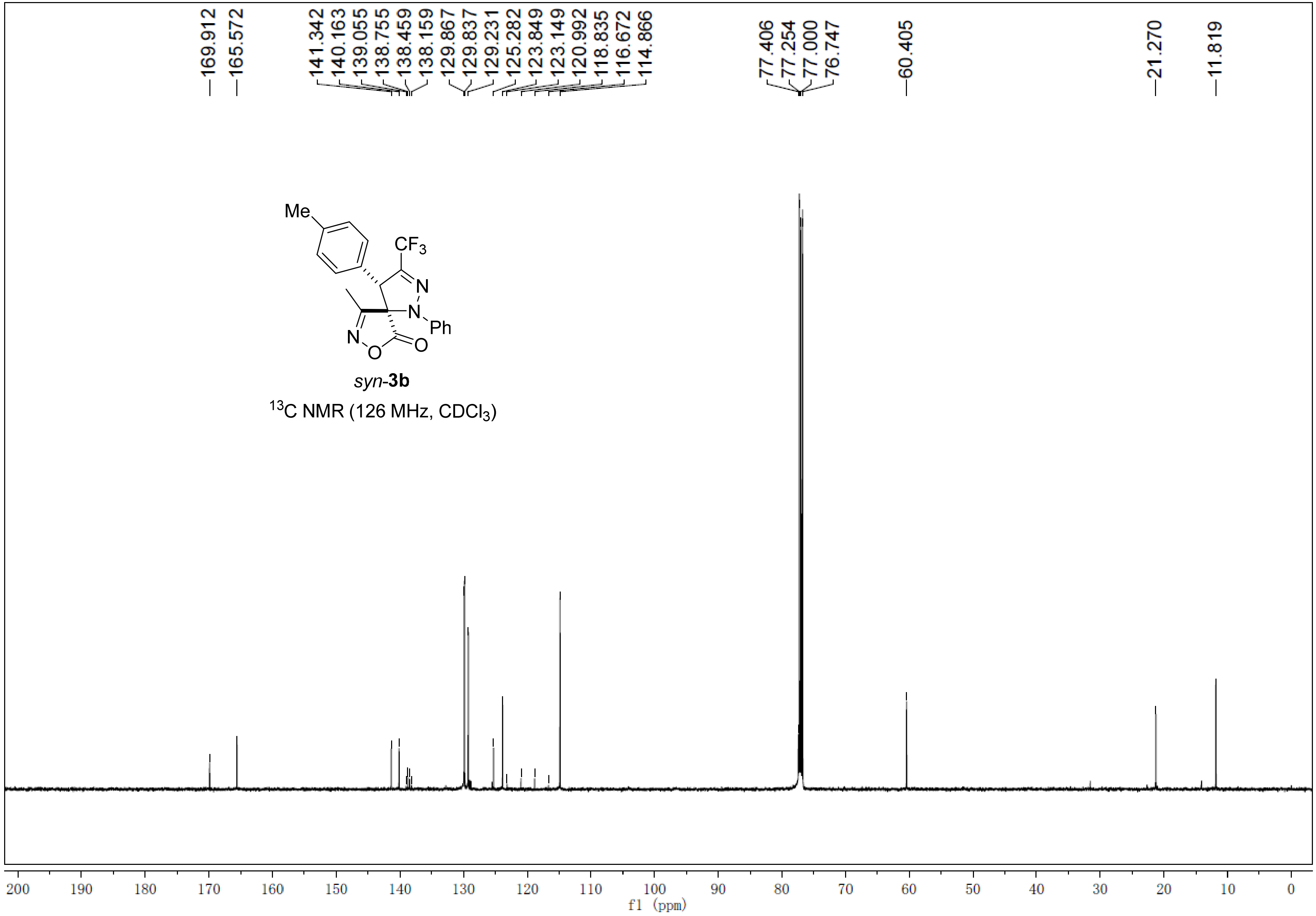
169.912
165.572
141.342
140.163
139.055
138.755
138.459
138.159
129.867
129.837
129.231
125.282
123.849
123.149
120.992
118.835
116.672
114.866
77.406
77.254
77.000
76.747
60.405
21.270
11.819
Me
CF3
N
N
Ph
N
O
O
syn-3b
13C NMR (126 MHz, CDCl3)
200
190
180
170
160
150
140
130
120
110
100
90
80
70
60
50
40
30
20
10
0
f1 (ppm)

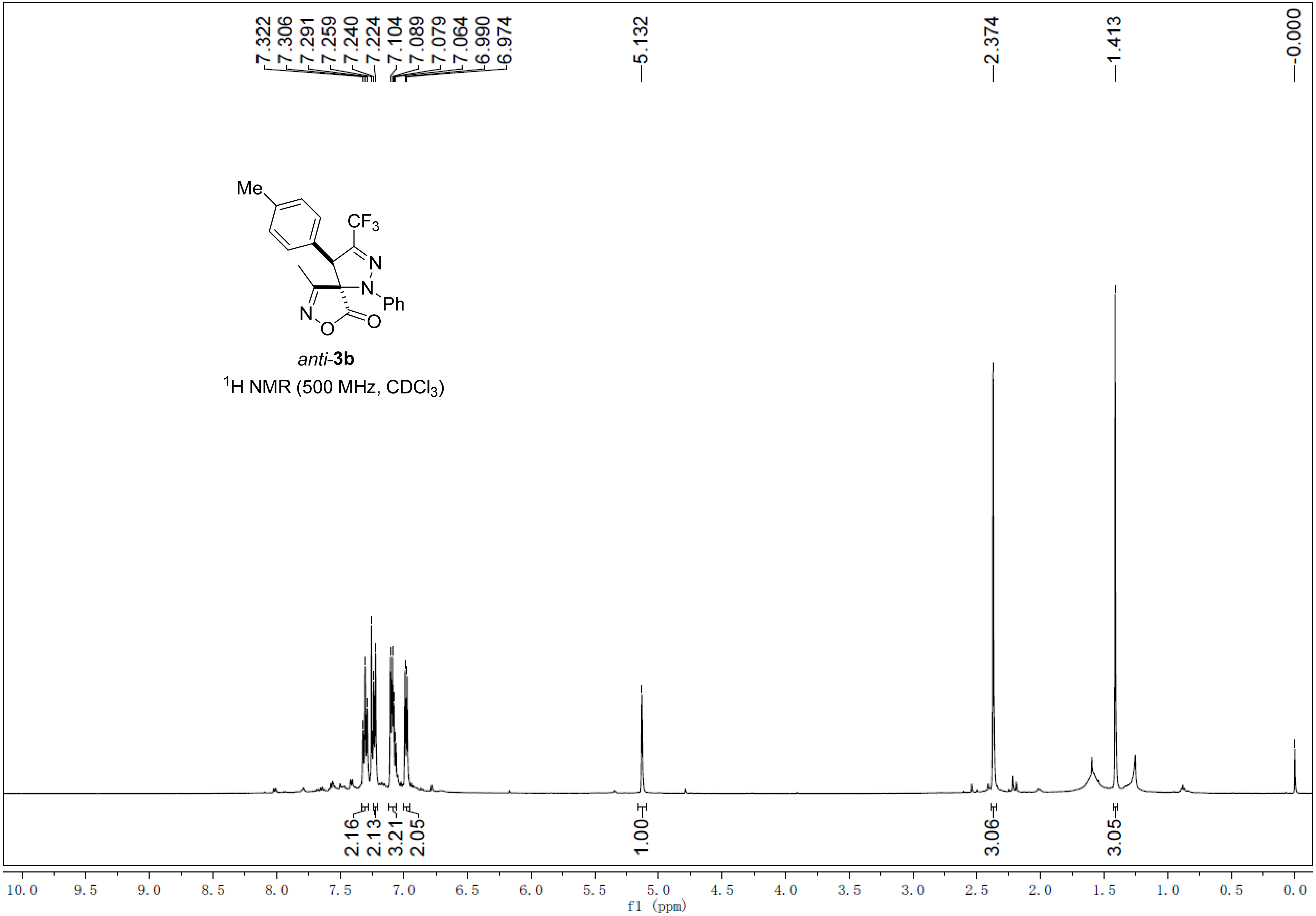
7.322
7.306
7.291
7.259
7.240
7.224
7.104
7.089
7.079
7.064
6.990
6.974
5.132
2.374
1.413
0.000
Me
CF3
N
N
Ph
N
O
O
anti-3b
1H NMR (500 MHz, CDCl3)
2.16
2.13
3.21
2.05
1.00
3.06
3.05
10.0
9.5
9.0
8.5
8.0
7.5
7.0
6.5
6.0
5.5
5.0
4.5
4.0
3.5
3.0
2.5
2.0
1.5
1.0
0.5
0.0
f1 (ppm)

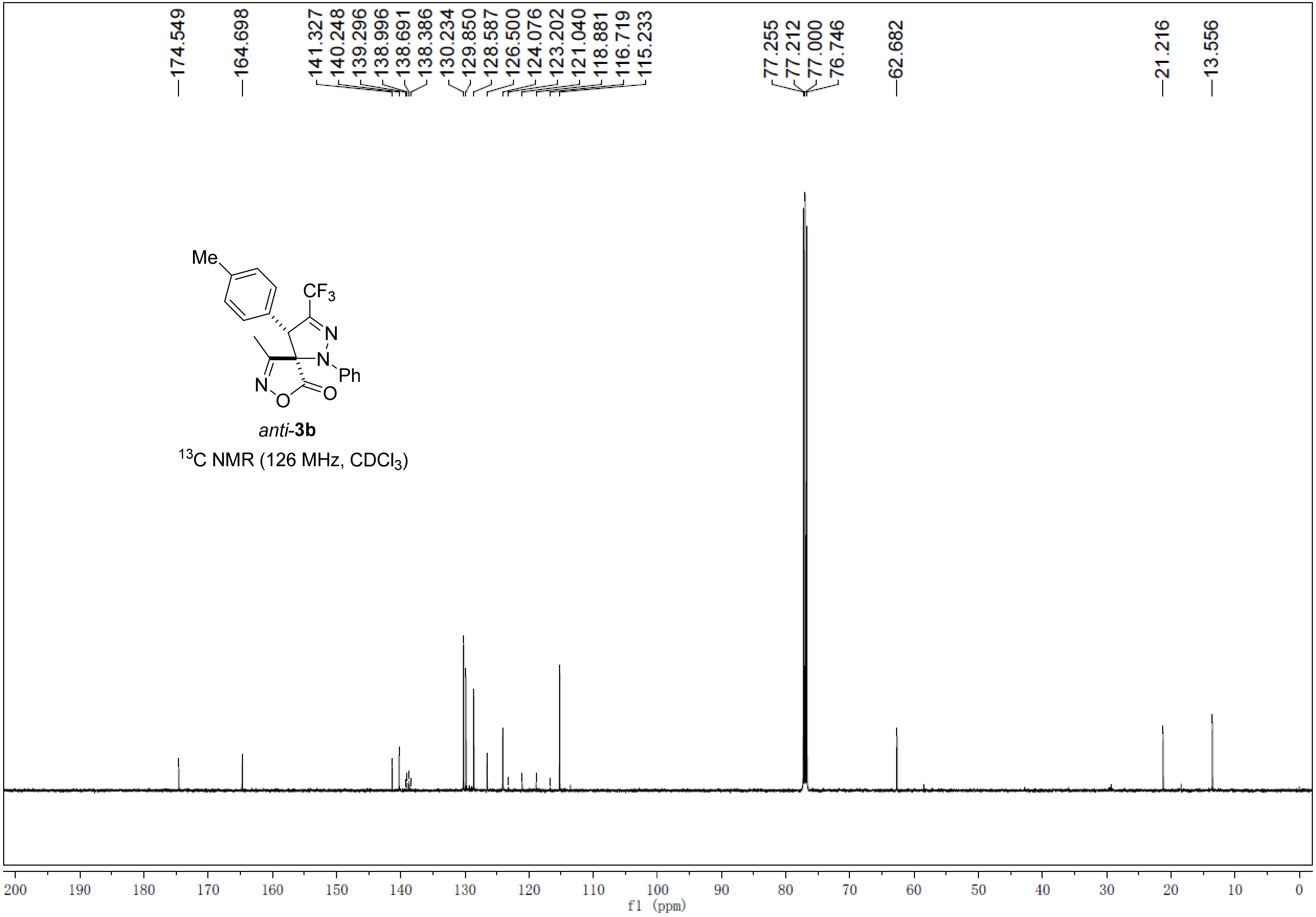
174.549
164.698
141.327
140.248
139.296
138.996
138.691
138.386
130.234
129.850
128.587
126.500
124.076
123.202
121.040
118.881
116.719
115.233
77.255
77.212
77.000
76.746
62.682
21.216
13.556
Me
CF3
N
N
Ph
N
O
O
anti-3b
13C NMR (126 MHz, CDCl3)
200 190 180 170 160 150 140 130 120 110 100 90 80 70 60 50 40 30 20 10 0
f1 (ppm)

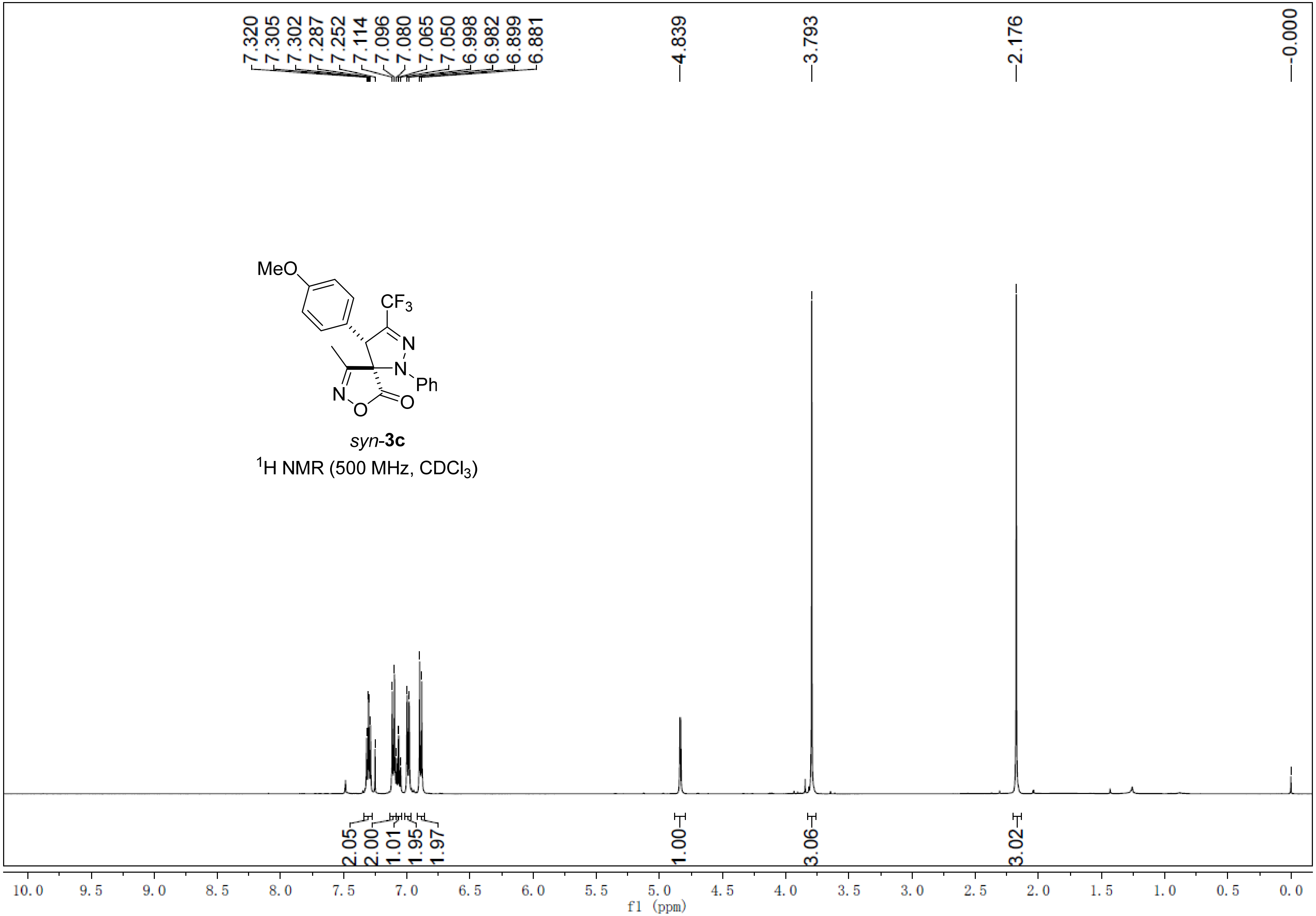
7.320
7.305
7.302
7.287
7.252
7.114
7.096
7.080
7.065
7.050
6.998
6.982
6.899
6.881
4.839
3.793
2.176
-0.000
MeO
CF3
N
N
Ph
N
O
O
syn-3c
1H NMR (500 MHz, CDCl3)
2.05
2.00
1.01
1.95
1.97
1.00
3.06
3.02
10.0
9.5
9.0
8.5
8.0
7.5
7.0
6.5
6.0
5.5
5.0
4.5
4.0
3.5
3.0
2.5
2.0
1.5
1.0
0.5
0.0
f1 (ppm)

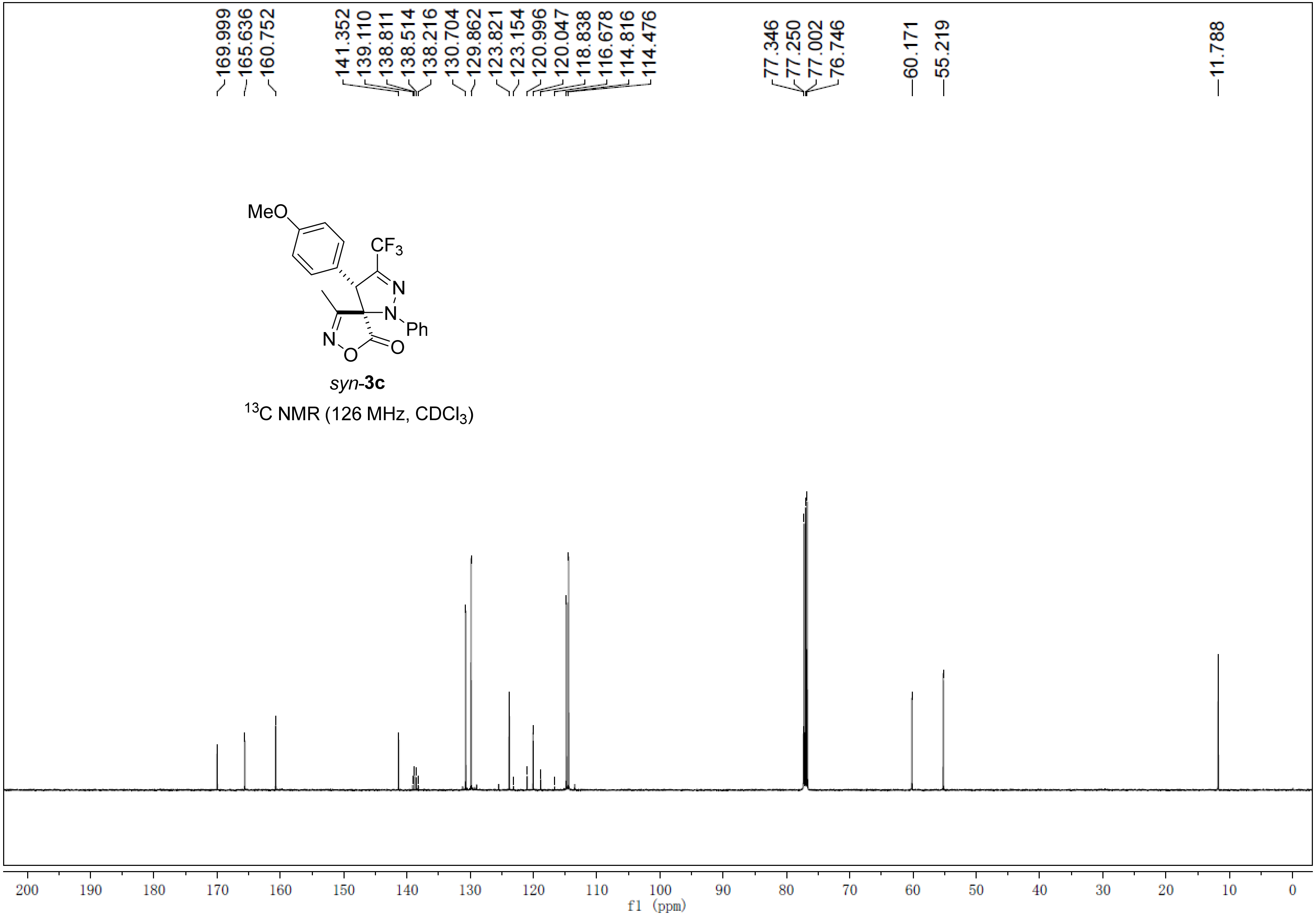
169.999
165.636
160.752
141.352
139.110
138.811
138.514
138.216
130.704
129.862
123.821
123.154
120.996
120.047
118.838
116.678
114.816
114.476
77.346
77.250
77.002
76.746
60.171
55.219
11.788
MeO
CF_3
N
N
Ph
N
O
O
syn-**3c**
^{13}C NMR (126 MHz, $CDCl_3$)
200
190
180
170
160
150
140
130
120
110
100
90
80
70
60
50
40
30
20
10
0
f1 (ppm)

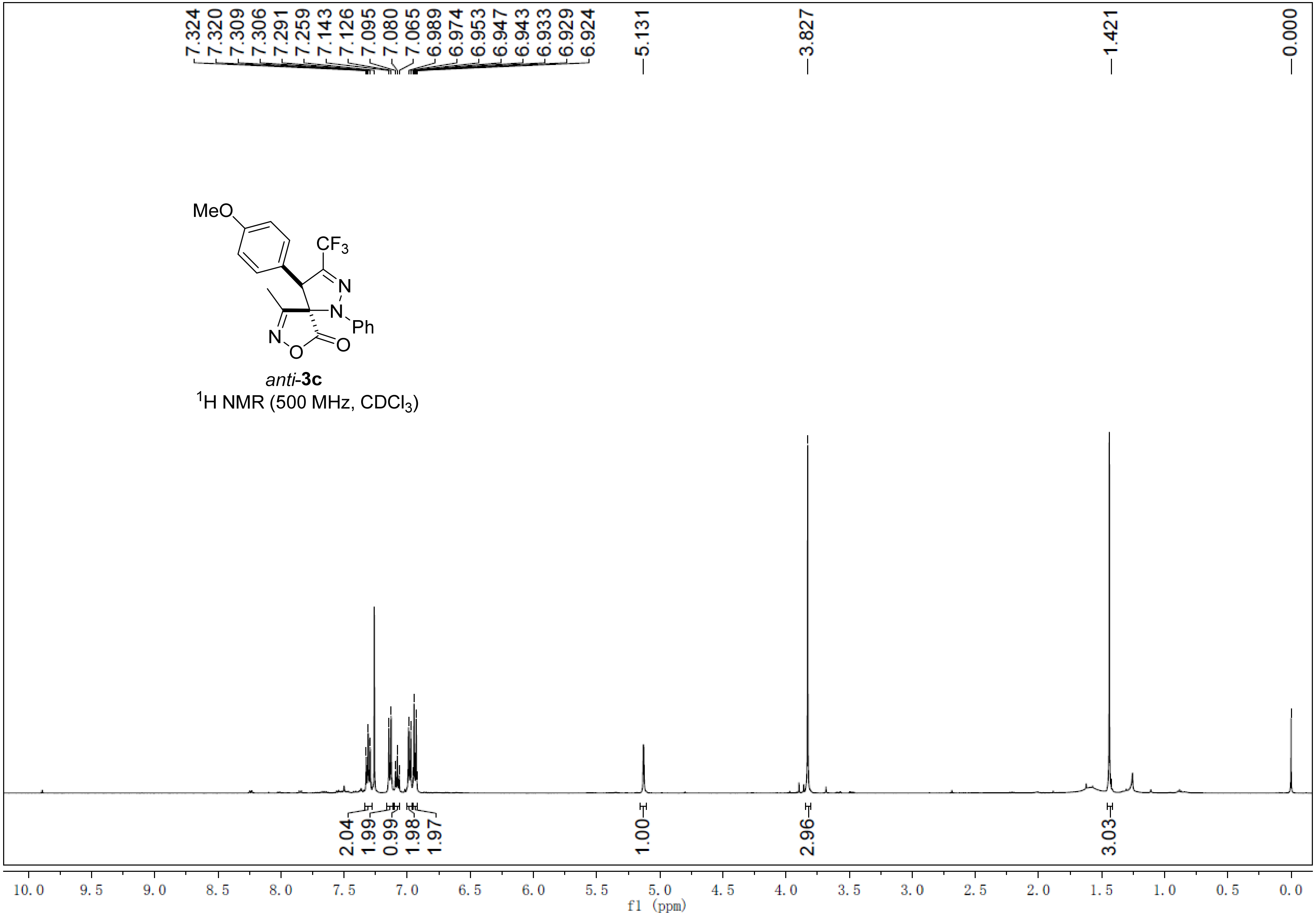
7.324
7.320
7.309
7.306
7.291
7.259
7.143
7.126
7.095
7.080
7.065
6.989
6.974
6.953
6.947
6.943
6.933
6.929
6.924
5.131
3.827
1.421
0.000
MeO
CF3
N
N
Ph
N
O
O
anti-3c
1H NMR (500 MHz, CDCl3)
2.04
1.99
0.99
1.98
1.97
1.00
2.96
3.03
10.0
9.5
9.0
8.5
8.0
7.5
7.0
6.5
6.0
5.5
5.0
4.5
4.0
3.5
3.0
2.5
2.0
1.5
1.0
0.5
0.0
f1 (ppm)

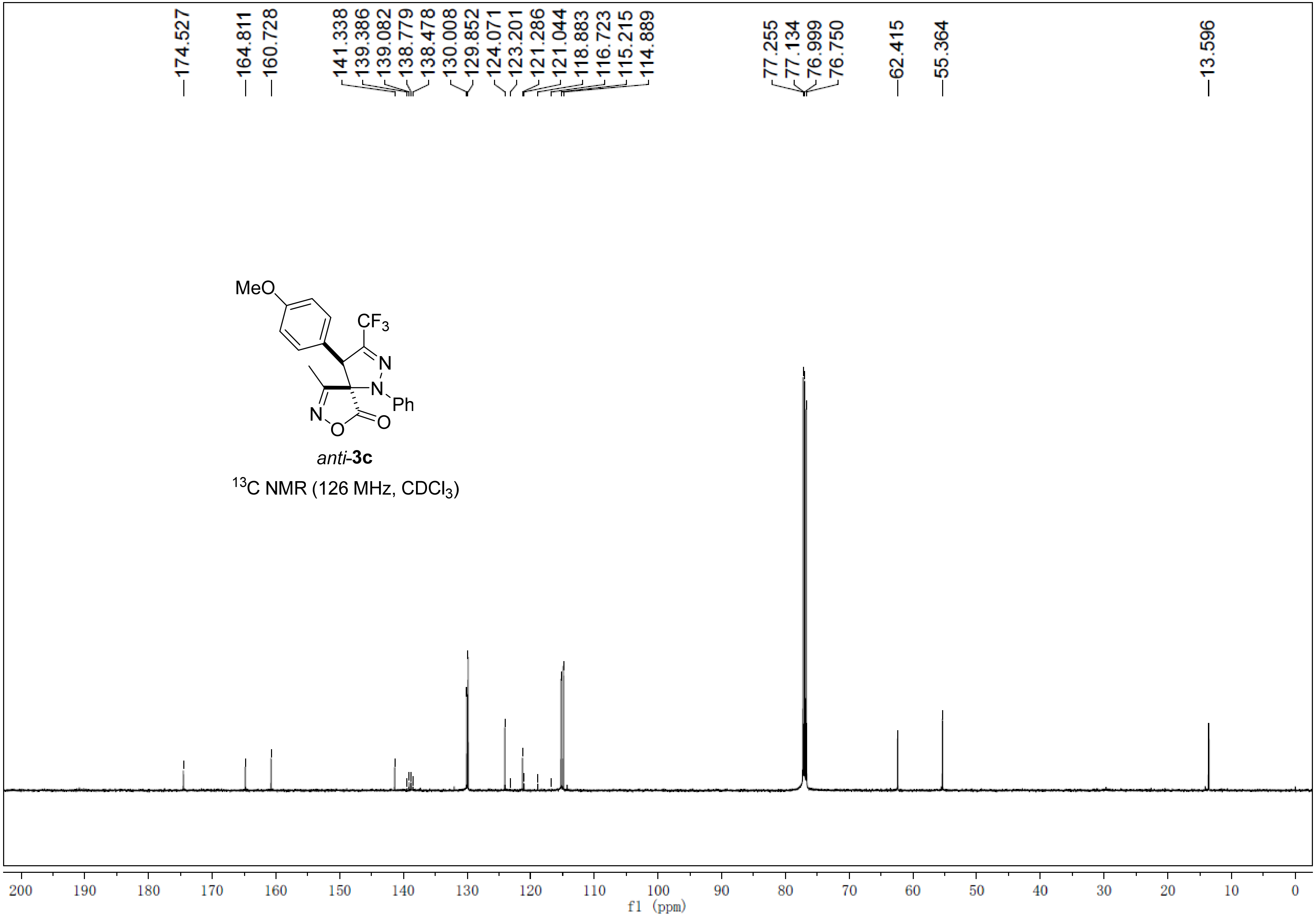

anti-**3c**

^{13}C NMR (126 MHz, $CDCl_3$)

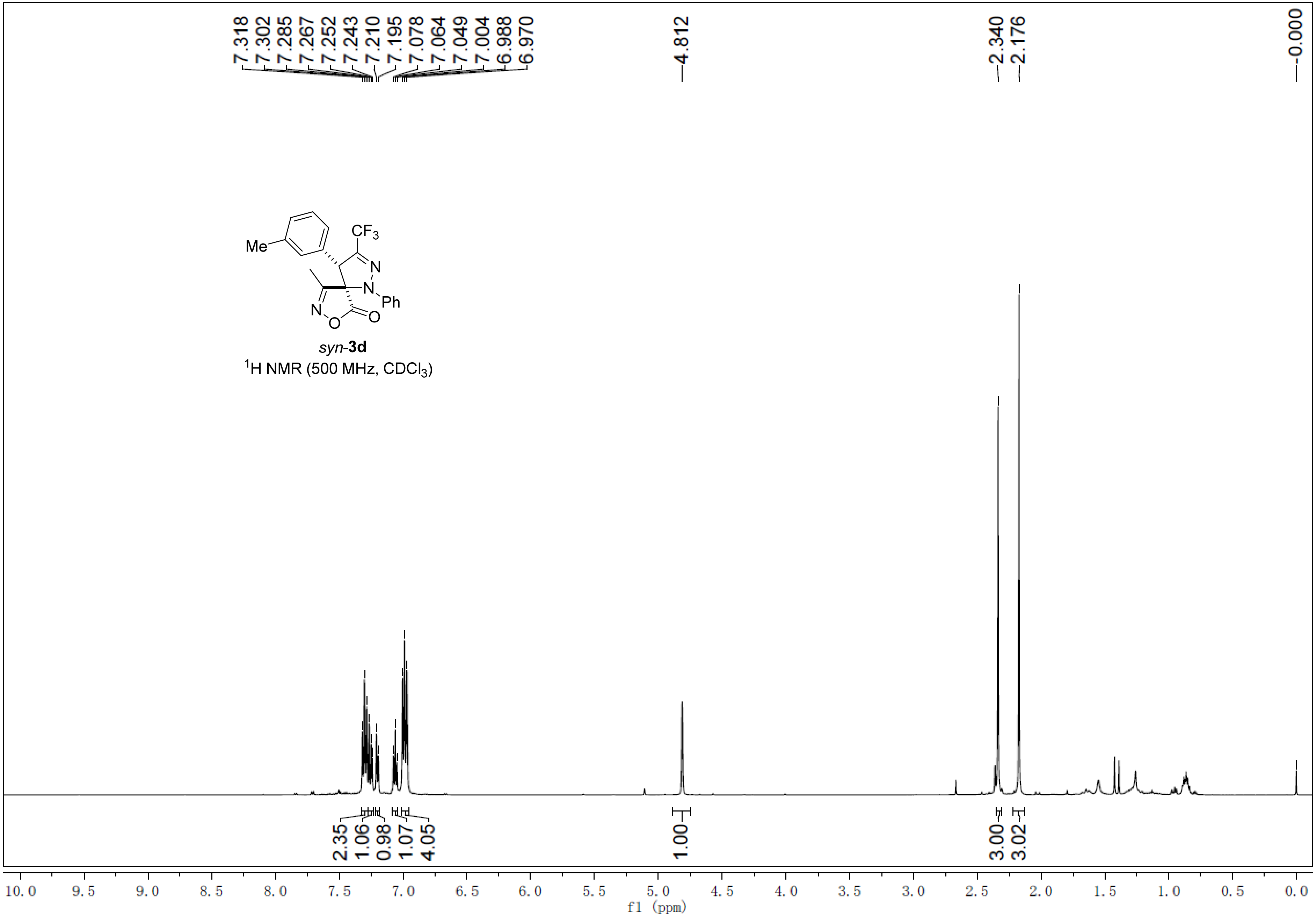
7.318
7.302
7.285
7.267
7.252
7.243
7.210
7.195
7.078
7.064
7.049
7.004
6.988
6.970
4.812
2.340
2.176
-0.000
CF3
Me
N
N
Ph
N
O
O
syn-3d
1H NMR (500 MHz, CDCl3)
2.35
1.06
0.98
1.07
4.05
1.00
3.00
3.02
10.0
9.5
9.0
8.5
8.0
7.5
7.0
6.5
6.0
5.5
5.0
4.5
4.0
3.5
3.0
2.5
2.0
1.5
1.0
0.5
0.0
f1 (ppm)

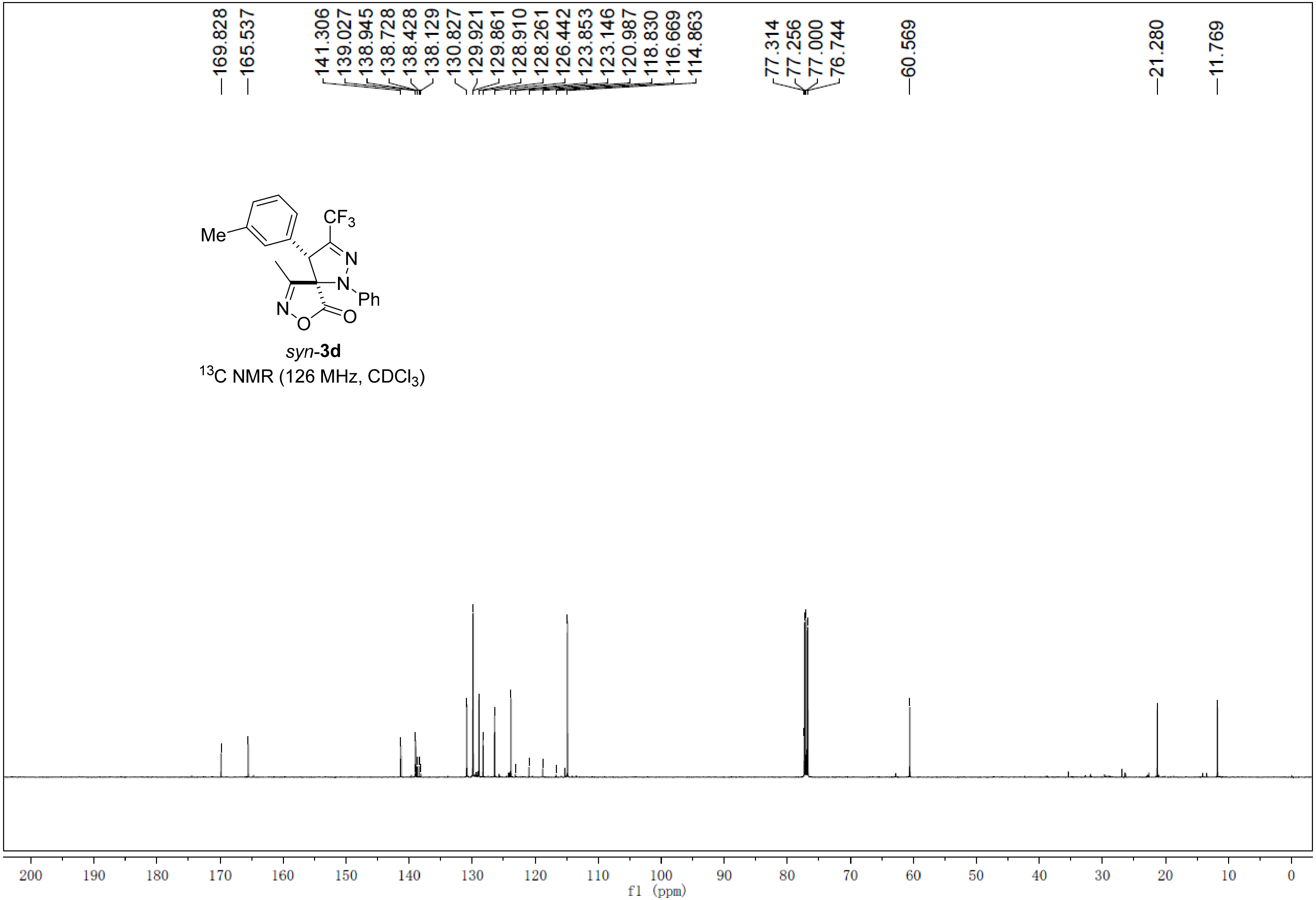

syn-**3d**

^{13}C NMR (126 MHz, $CDCl_3$)

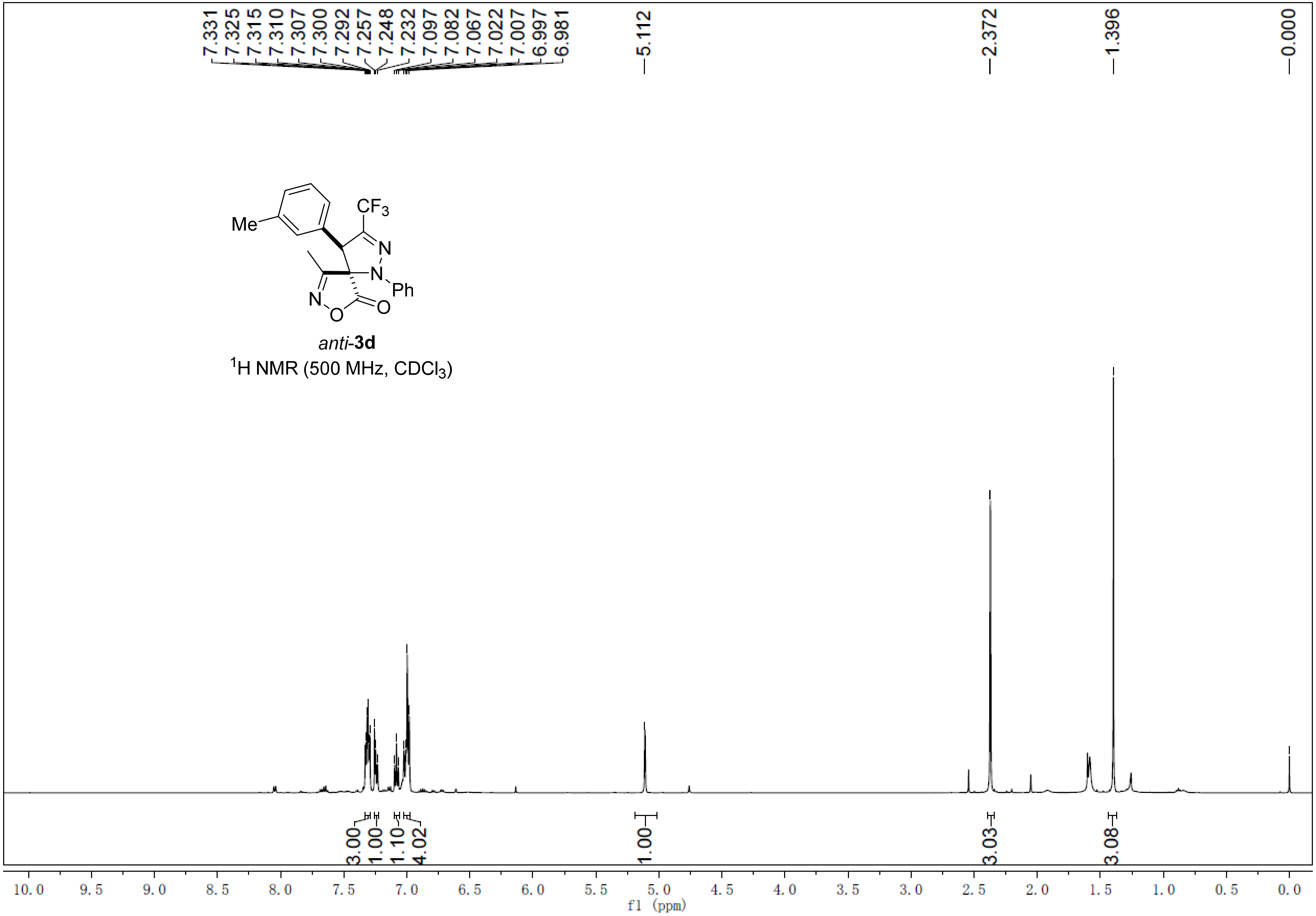
7.331
7.325
7.315
7.310
7.307
7.300
7.292
7.257
7.248
7.232
7.097
7.082
7.067
7.022
7.007
6.997
6.981
5.112
2.372
1.396
0.000
Me
CF_3
N
N
Ph
N
O
O
anti-**3d**
^{1}H NMR (500 MHz, $CDCl_3$)
3.00
1.00
1.10
4.02
1.00
3.03
3.08
10.0 9.5 9.0 8.5 8.0 7.5 7.0 6.5 6.0 5.5 5.0 4.5 4.0 3.5 3.0 2.5 2.0 1.5 1.0 0.5 0.0
f1 (ppm)

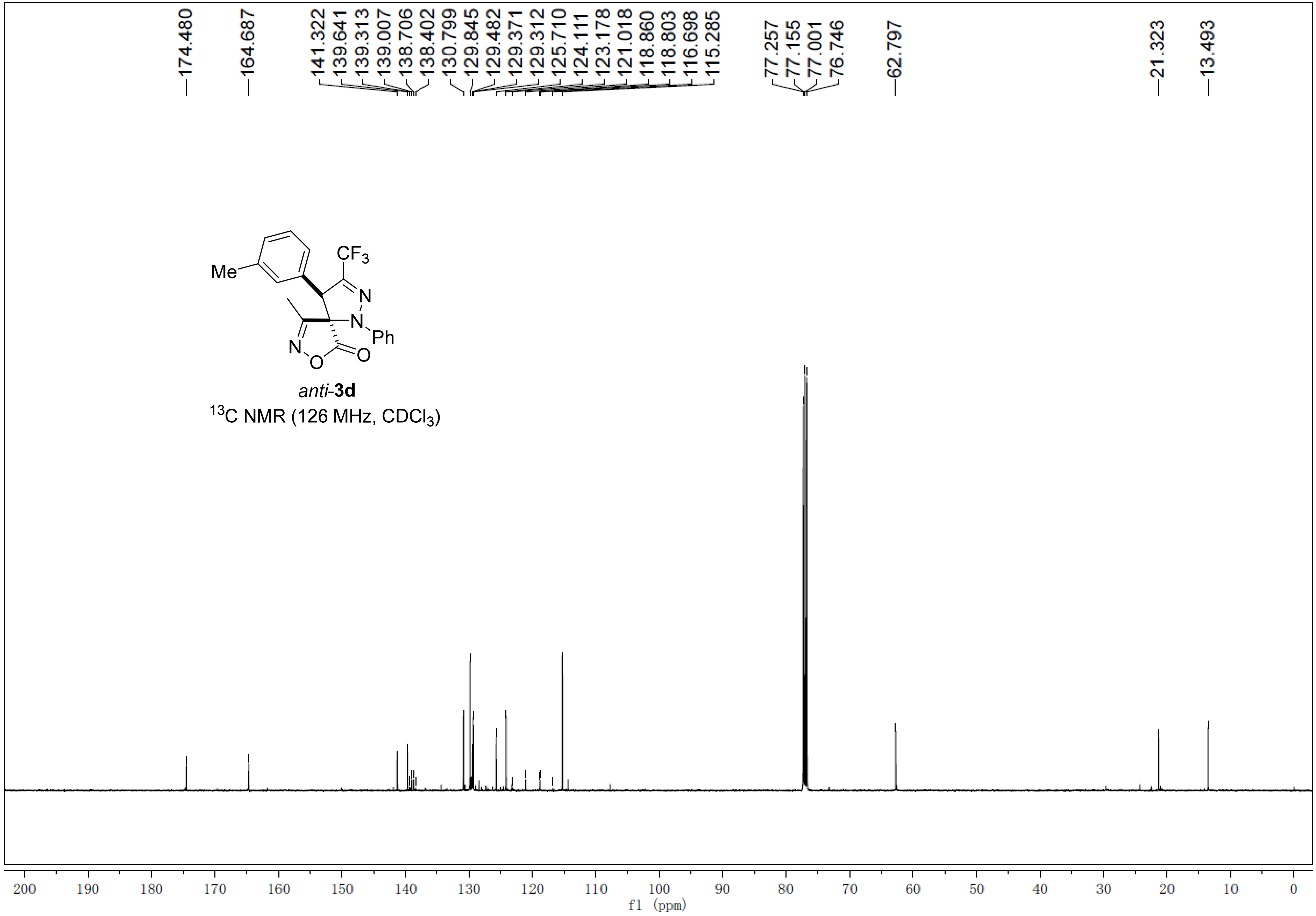

174.480
164.687
141.322
139.641
139.313
139.007
138.706
138.402
130.799
129.845
129.482
129.371
129.312
125.710
124.111
123.178
121.018
118.860
118.803
116.698
115.285
77.257
77.155
77.001
76.746
62.797
21.323
13.493
Me
CF3
N
N
Ph
N
O
O
anti-3d
13C NMR (126 MHz, CDCl3)
200
190
180
170
160
150
140
130
120
110
100
90
80
70
60
50
40
30
20
10
0
f1 (ppm)

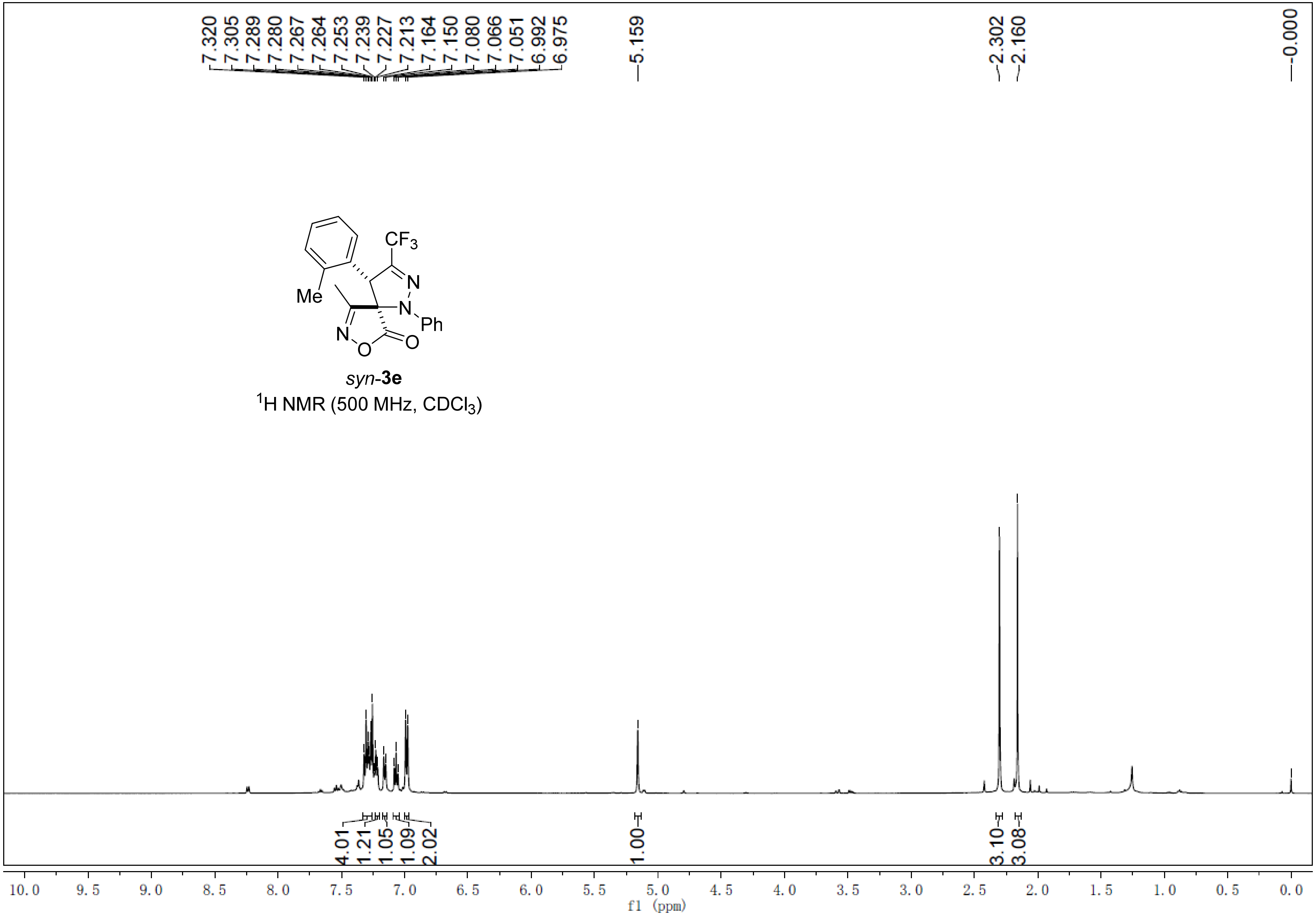
7.320
7.305
7.289
7.280
7.267
7.264
7.253
7.239
7.227
7.213
7.164
7.150
7.080
7.066
7.051
6.992
6.975
5.159
2.302
2.160
0.000
CF3
N
Me
N
Ph
N
O
O
syn-3e
1H NMR (500 MHz, CDCl3)
4.01
1.21
1.05
1.09
2.02
1.00
3.10
3.08
10.0
9.5
9.0
8.5
8.0
7.5
7.0
6.5
6.0
5.5
5.0
4.5
4.0
3.5
3.0
2.5
2.0
1.5
1.0
0.5
0.0
f1 (ppm)

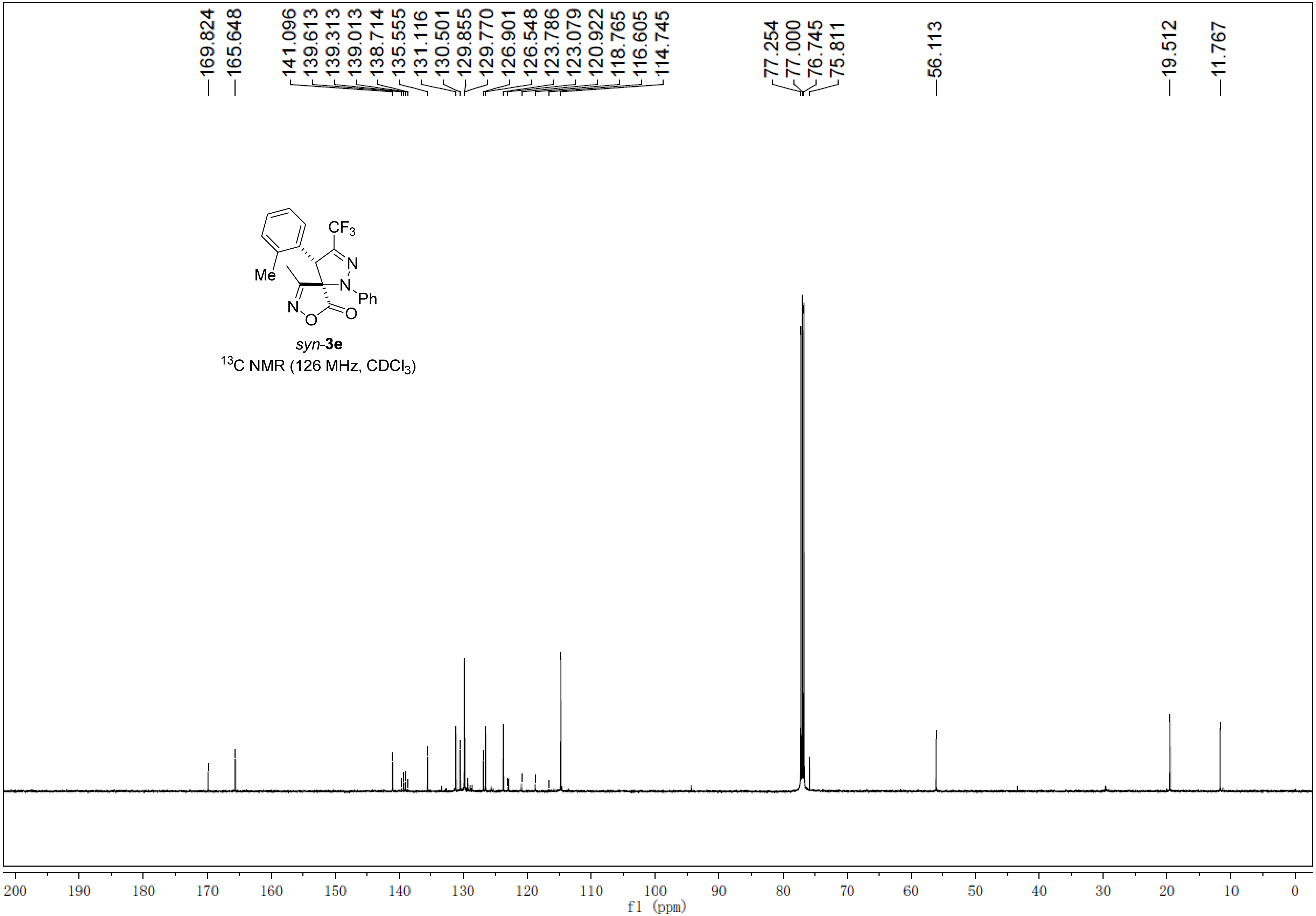
169.824
165.648
141.096
139.613
139.313
139.013
138.714
135.555
131.116
130.501
129.855
129.770
126.901
126.548
123.786
123.079
120.922
118.765
116.605
114.745
77.254
77.000
76.745
75.811
56.113
19.512
11.767
CF3
Me
N
N
Ph
N
O
O
syn-3e
13C NMR (126 MHz, CDCl3)
200
190
180
170
160
150
140
130
120
110
100
90
80
70
60
50
40
30
20
10
0
f1 (ppm)

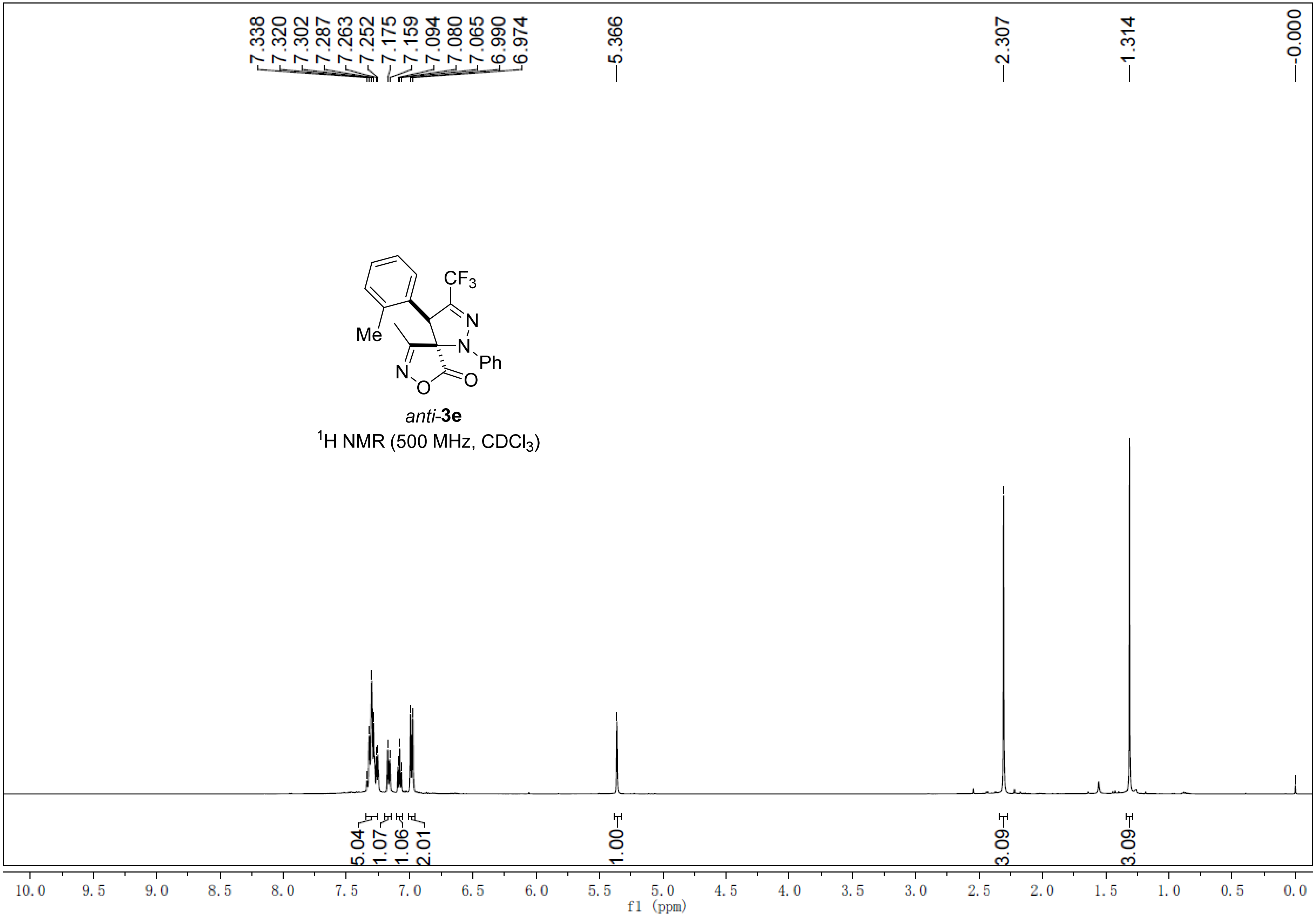
7.338
7.320
7.302
7.287
7.263
7.252
7.175
7.159
7.094
7.080
7.065
6.990
6.974
5.366
2.307
1.314
0.000
CF3
N
N
Me
Ph
N
O
O
anti-3e
1H NMR (500 MHz, CDCl3)
5.04
1.07
1.06
2.01
1.00
3.09
3.09
10.0
9.5
9.0
8.5
8.0
7.5
7.0
6.5
6.0
5.5
5.0
4.5
4.0
3.5
3.0
2.5
2.0
1.5
1.0
0.5
0.0
f1 (ppm)

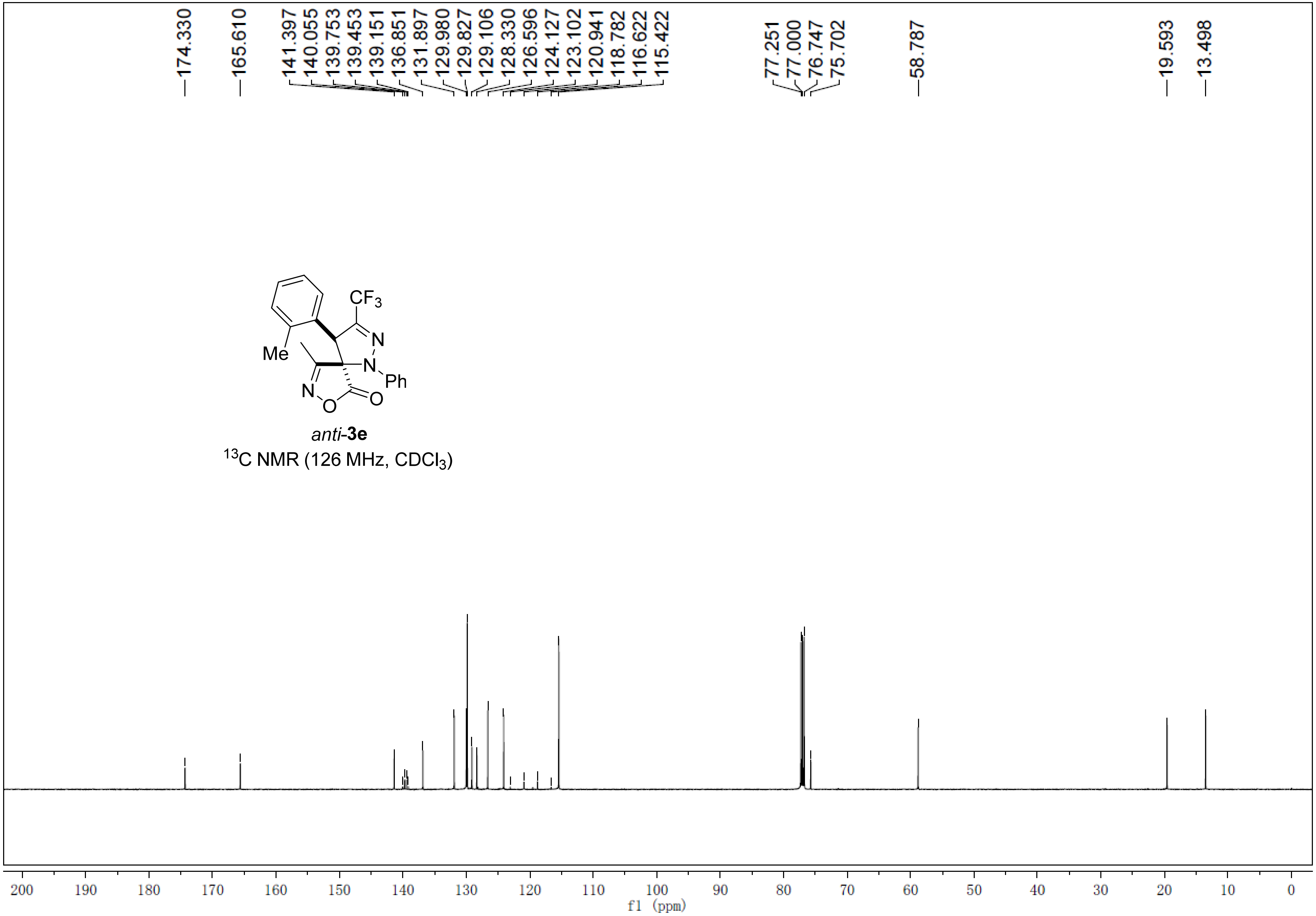

anti-**3e**

^{13}C NMR (126 MHz, $CDCl_3$)

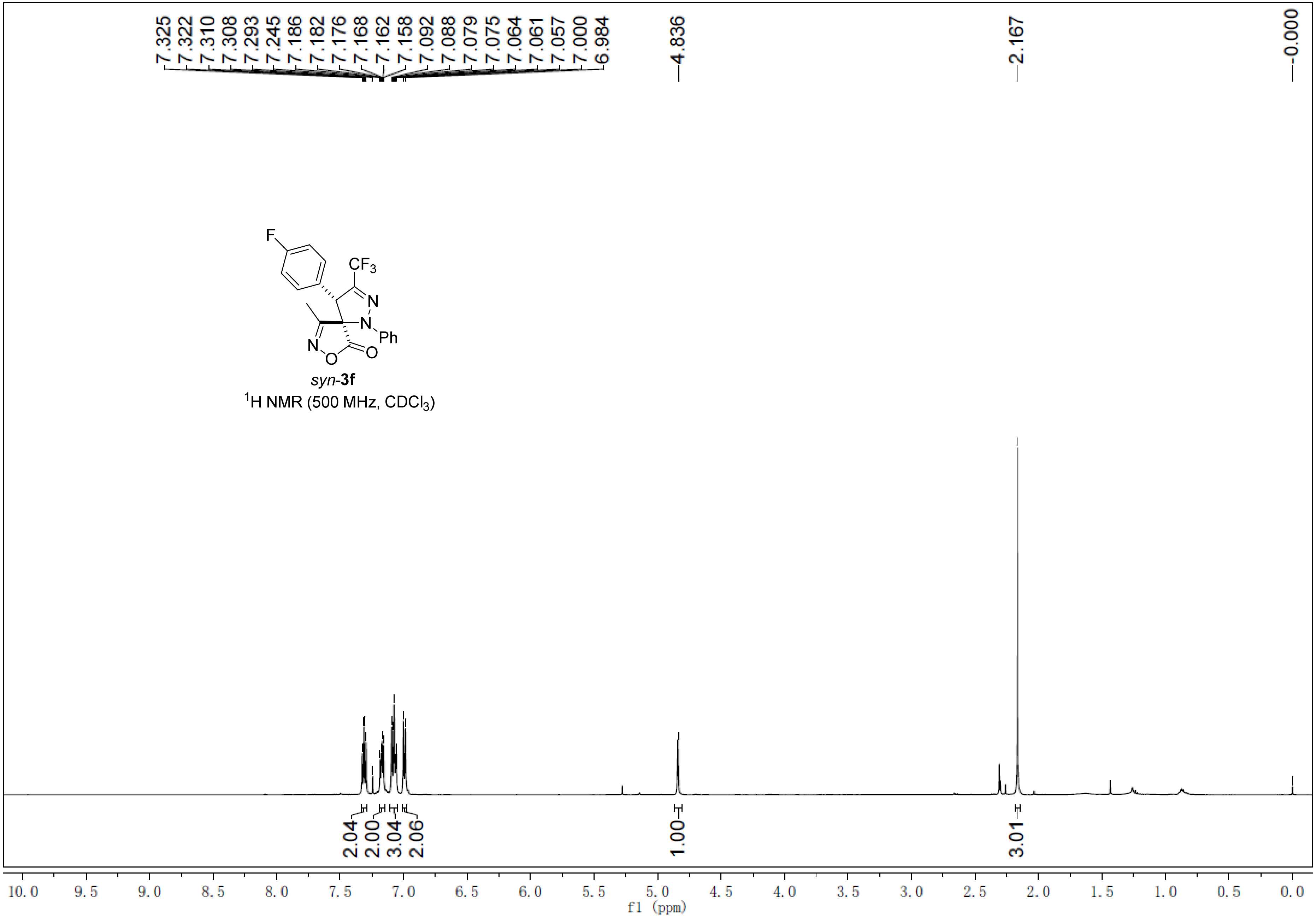

7.325
7.322
7.310
7.308
7.293
7.245
7.186
7.182
7.176
7.168
7.162
7.158
7.092
7.088
7.079
7.075
7.064
7.061
7.057
7.000
6.984
4.836
2.167
-0.000
F
CF3
N
N
Ph
N
O
O
syn-3f
1H NMR (500 MHz, CDCl3)
2.04
2.00
3.04
2.06
1.00
3.01
10.0
9.5
9.0
8.5
8.0
7.5
7.0
6.5
6.0
5.5
5.0
4.5
4.0
3.5
3.0
2.5
2.0
1.5
1.0
0.5
0.0
f1 (ppm)

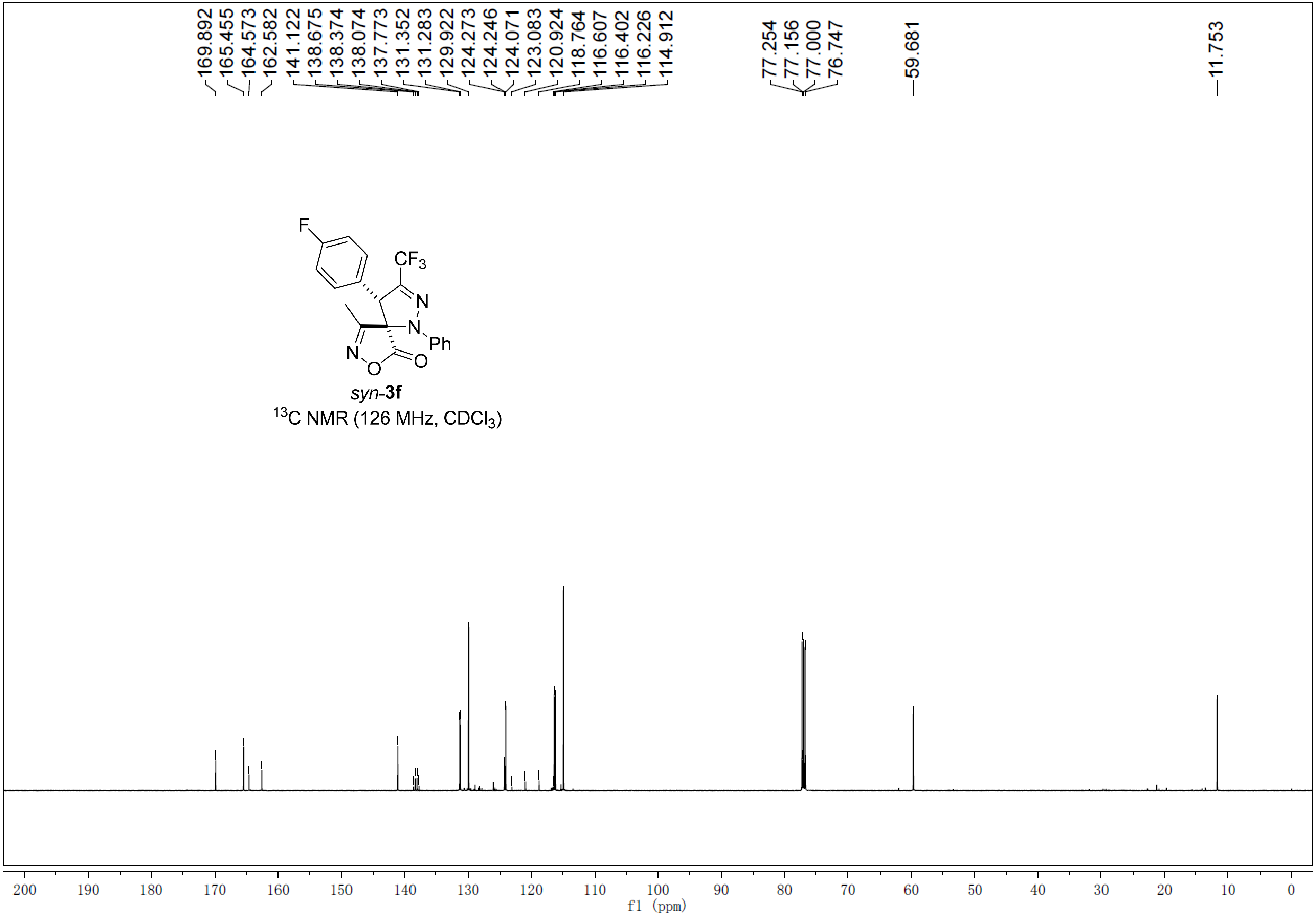
169.892
165.455
164.573
162.582
141.122
138.675
138.374
138.074
137.773
131.352
131.283
129.922
124.273
124.246
124.071
123.083
120.924
118.764
116.607
116.402
116.226
114.912
77.254
77.156
77.000
76.747
59.681
11.753
F
CF_3
N
N
Ph
N
O
O
syn-**3f**
^{13}C NMR (126 MHz, $CDCl_3$)
200
190
180
170
160
150
140
130
120
110
100
90
80
70
60
50
40
30
20
10
0
f1 (ppm)

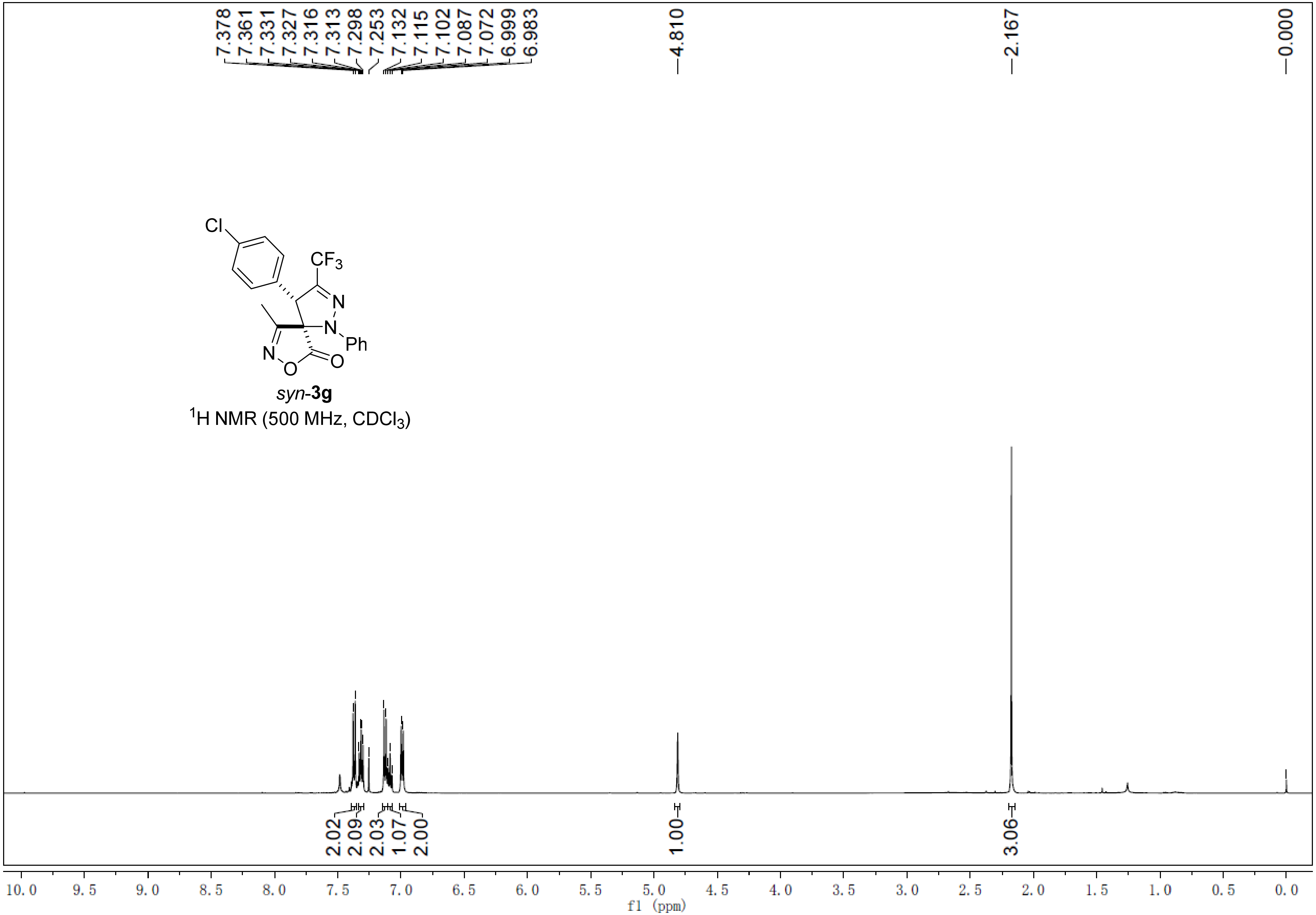

syn-**3g**

^{1}H NMR (500 MHz, $CDCl_3$)

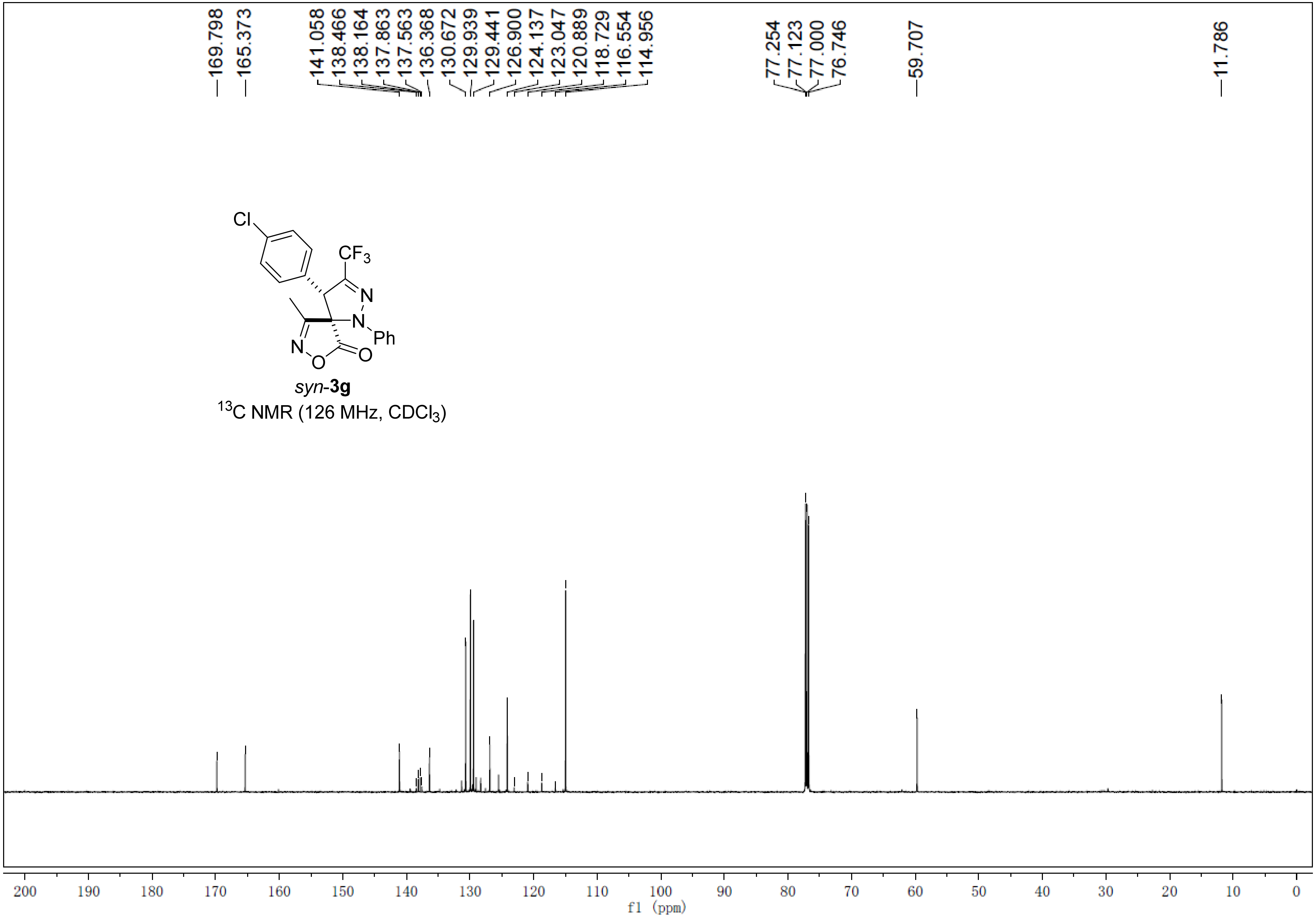
169.798
165.373
141.058
138.466
138.164
137.863
137.563
136.368
130.672
129.939
129.441
126.900
124.137
123.047
120.889
118.729
116.554
114.956
77.254
77.123
77.000
76.746
59.707
11.786
Cl
CF3
N
N
Ph
N
O
O
syn-3g
13C NMR (126 MHz, CDCl3)
200
190
180
170
160
150
140
130
120
110
100
90
80
70
60
50
40
30
20
10
0
f1 (ppm)

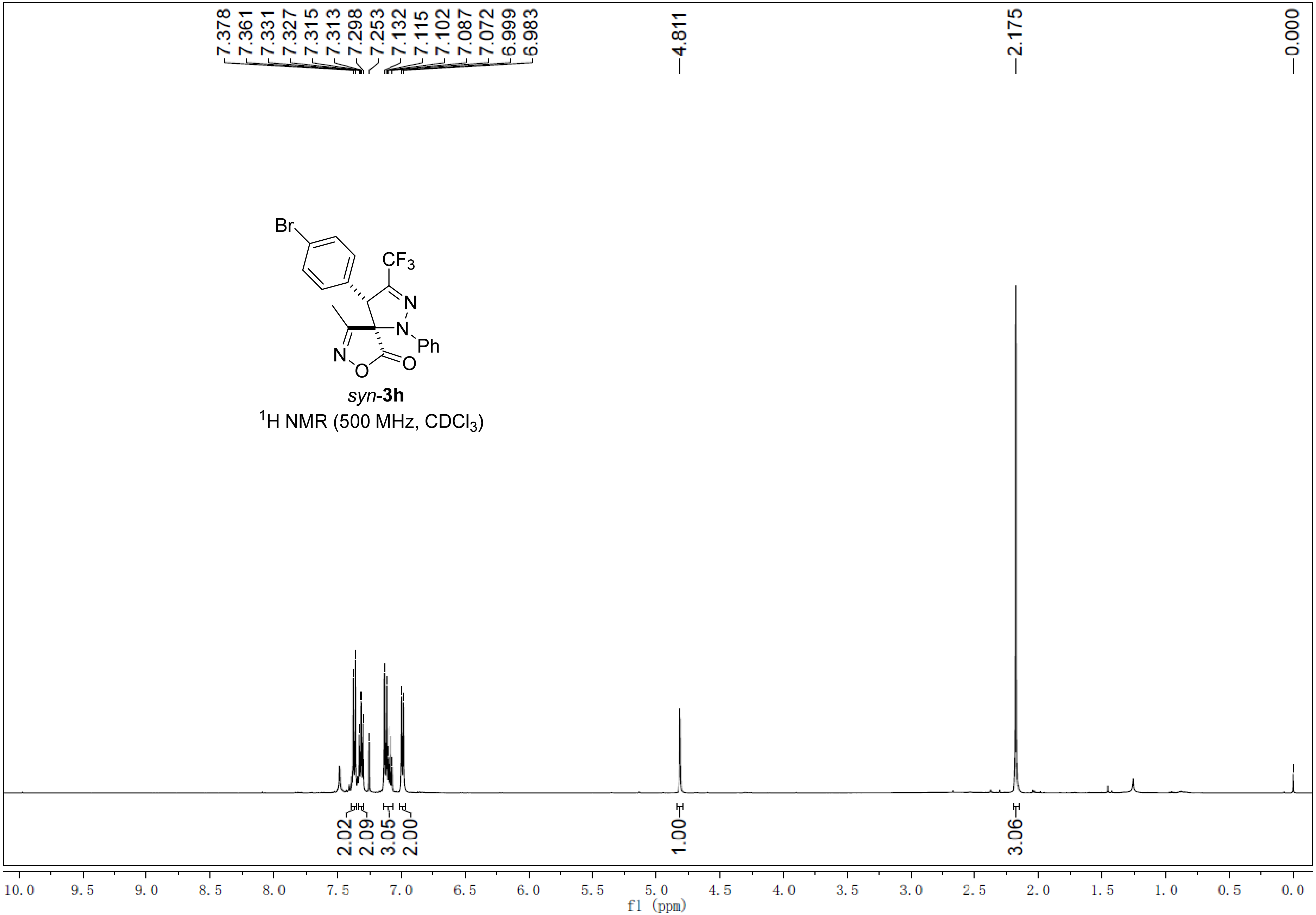

7.378
7.361
7.331
7.327
7.315
7.313
7.298
7.253
7.132
7.115
7.102
7.087
7.072
6.999
6.983
4.811
2.175
0.000
Br
CF_3
N
N
Ph
N
O
O
syn-**3h**
^{1}H NMR (500 MHz, $CDCl_3$)
2.02
2.09
3.05
2.00
1.00
3.06
10.0
9.5
9.0
8.5
8.0
7.5
7.0
6.5
6.0
5.5
5.0
4.5
4.0
3.5
3.0
2.5
2.0
1.5
1.0
0.5
0.0
f1 (ppm)

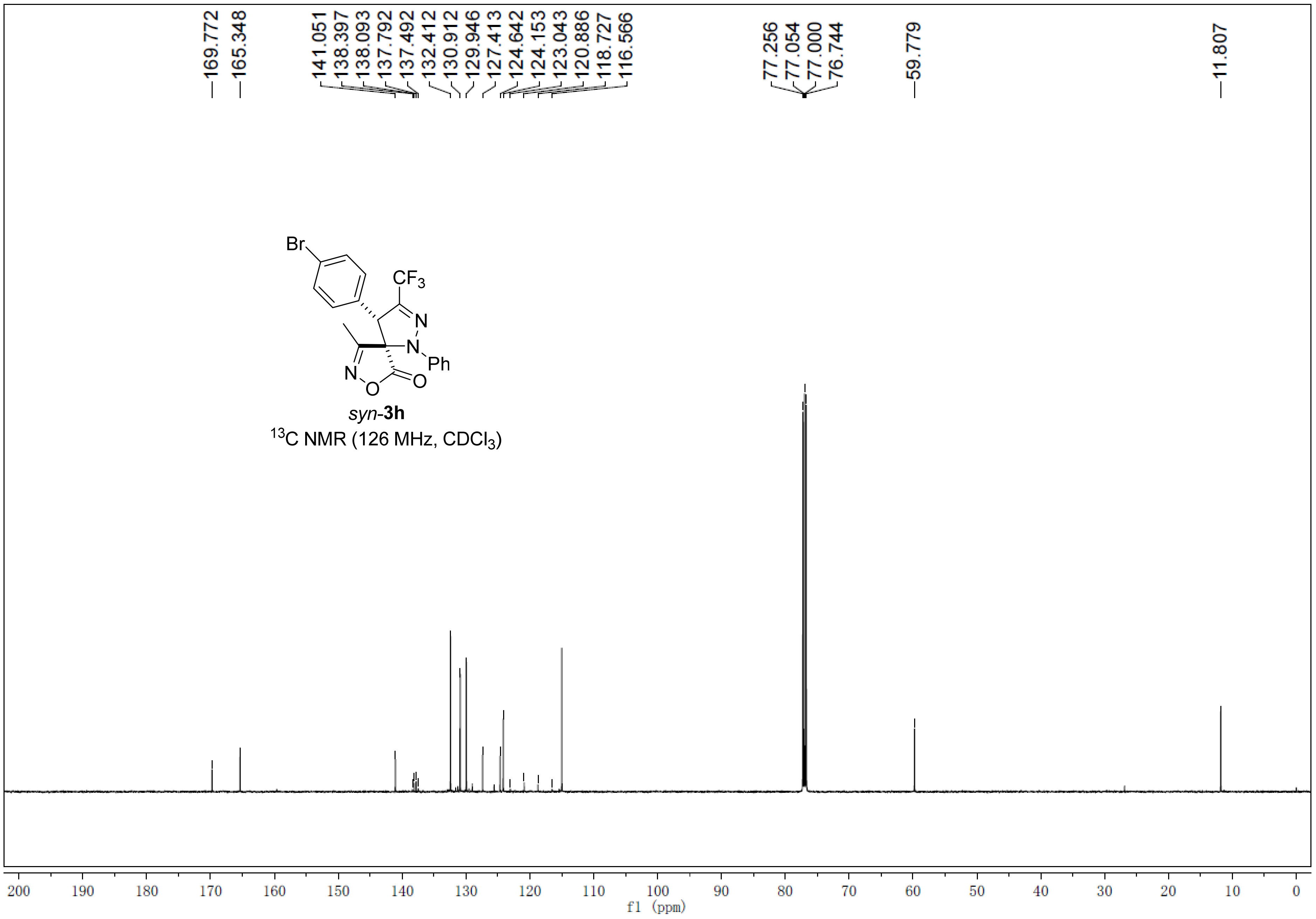

169.772
165.348
141.051
138.397
138.093
137.792
137.492
132.412
130.912
129.946
127.413
124.642
124.153
123.043
120.886
118.727
116.566
77.256
77.054
77.000
76.744
59.779
11.807
Br
CF3
N
N
Ph
N
O
O
syn-3h
13C NMR (126 MHz, CDCl3)
200
190
180
170
160
150
140
130
120
110
100
90
80
70
60
50
40
30
20
10
0
f1 (ppm)

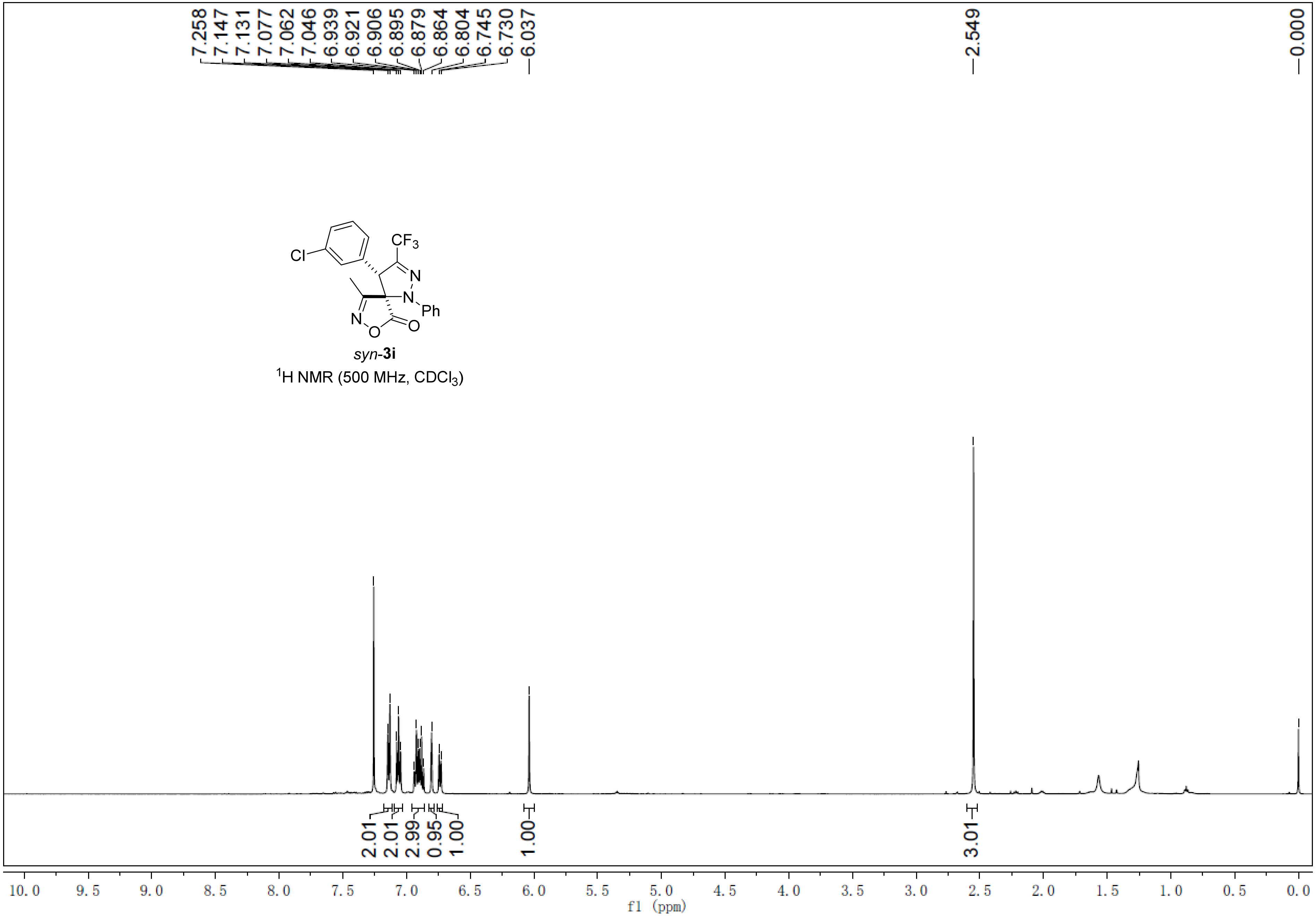
7.258
7.147
7.131
7.077
7.062
7.046
6.939
6.921
6.906
6.895
6.879
6.864
6.804
6.745
6.730
6.037
2.549
0.000
CF3
Cl
N
N
Ph
N
O
O
syn-3i
1H NMR (500 MHz, CDCl3)
2.01
2.01
2.99
0.95
1.00
1.00
3.01
10.0
9.5
9.0
8.5
8.0
7.5
7.0
6.5
6.0
5.5
5.0
4.5
4.0
3.5
3.0
2.5
2.0
1.5
1.0
0.5
0.0
f1 (ppm)

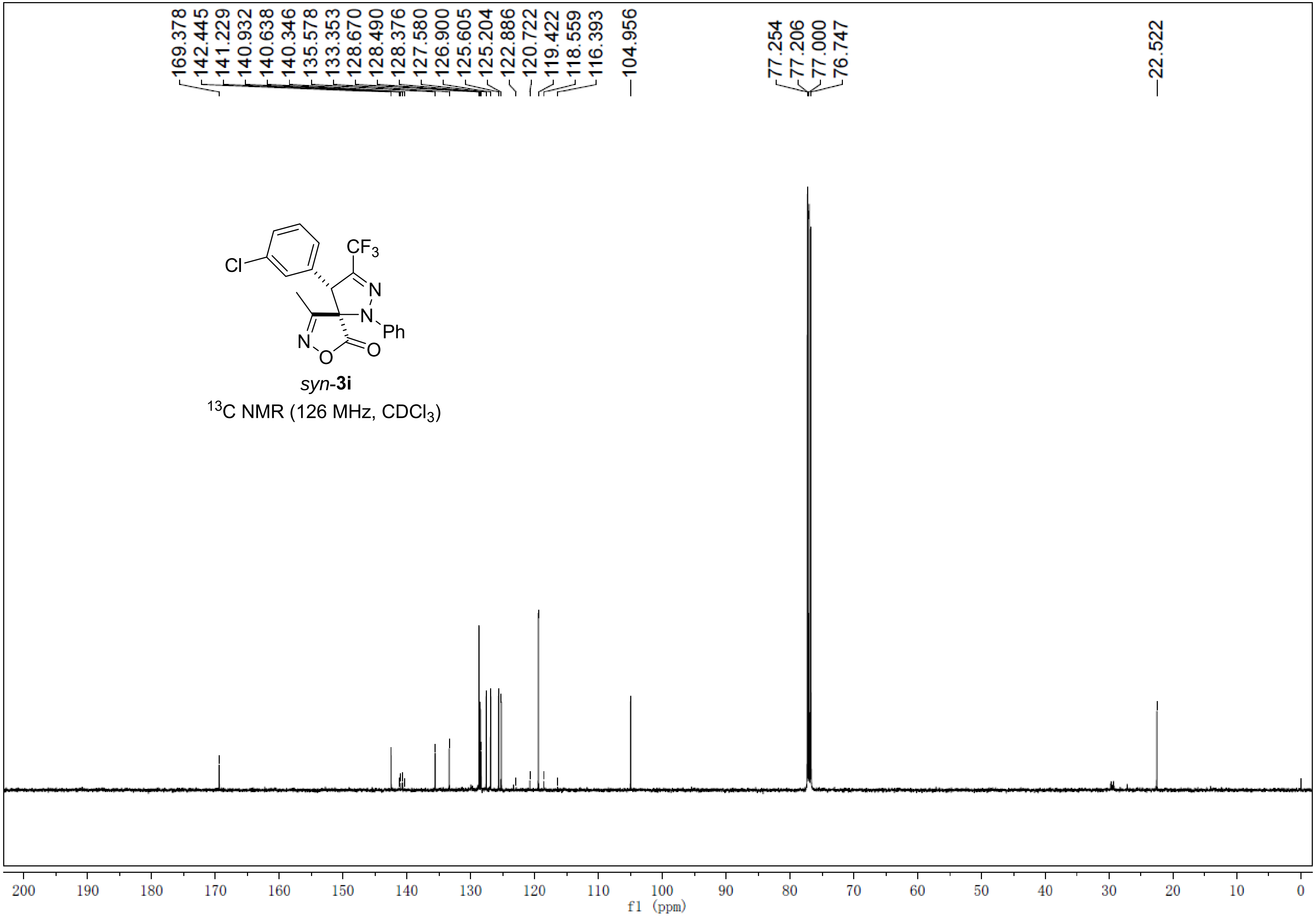
169.378
142.445
141.229
140.932
140.638
140.346
135.578
133.353
128.670
128.490
128.376
127.580
126.900
125.605
125.204
122.886
120.722
119.422
118.559
116.393
104.956
77.254
77.206
77.000
76.747
22.522
Cl
CF3
N
N
Ph
N
O
O
syn-3i
13C NMR (126 MHz, CDCl3)
200 190 180 170 160 150 140 130 120 110 100 90 80 70 60 50 40 30 20 10 0
f1 (ppm)

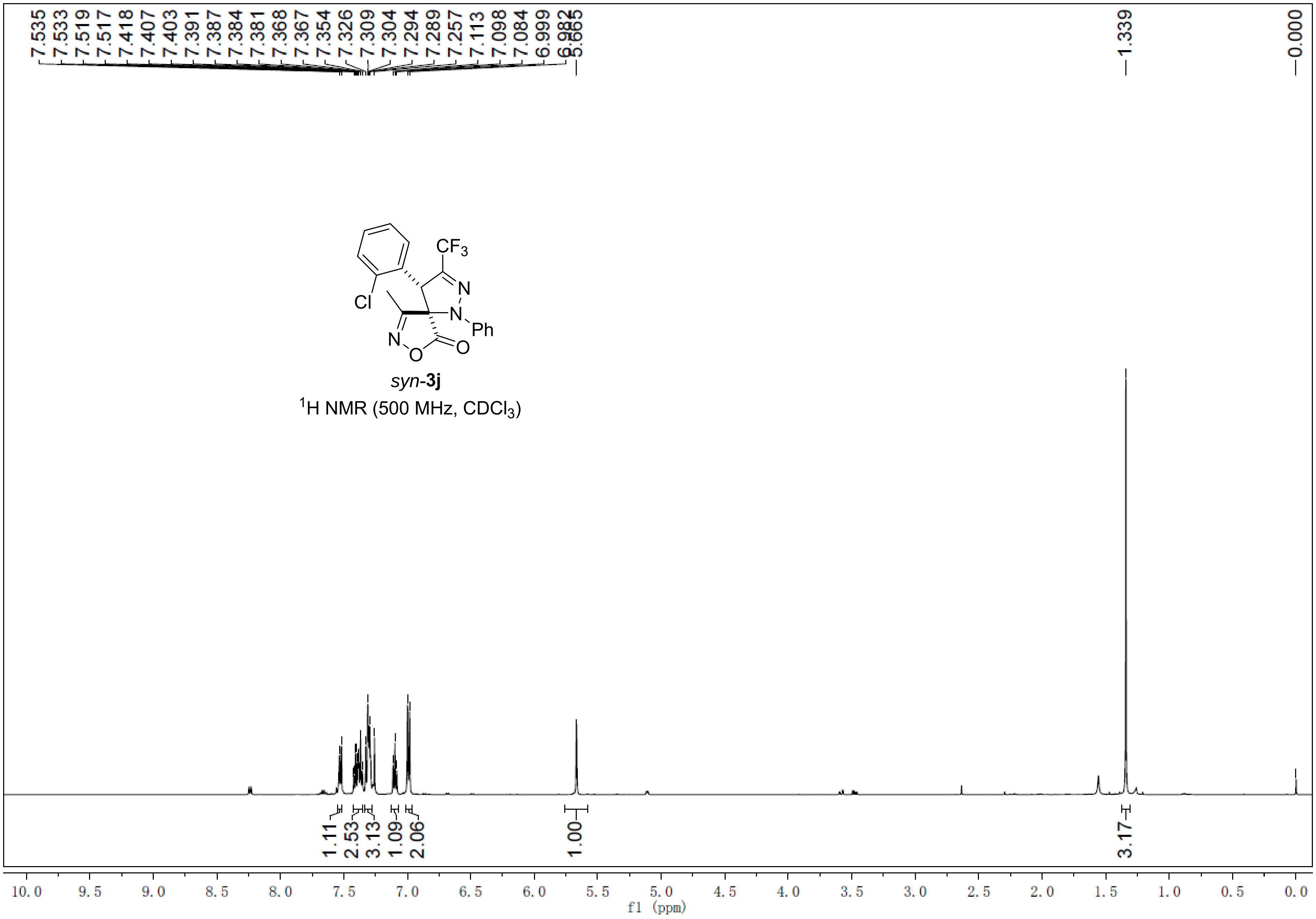
CF3
Cl
N
N
Ph
N
O
O
syn-3j
1H NMR (500 MHz, CDCl3)
f1 (ppm)

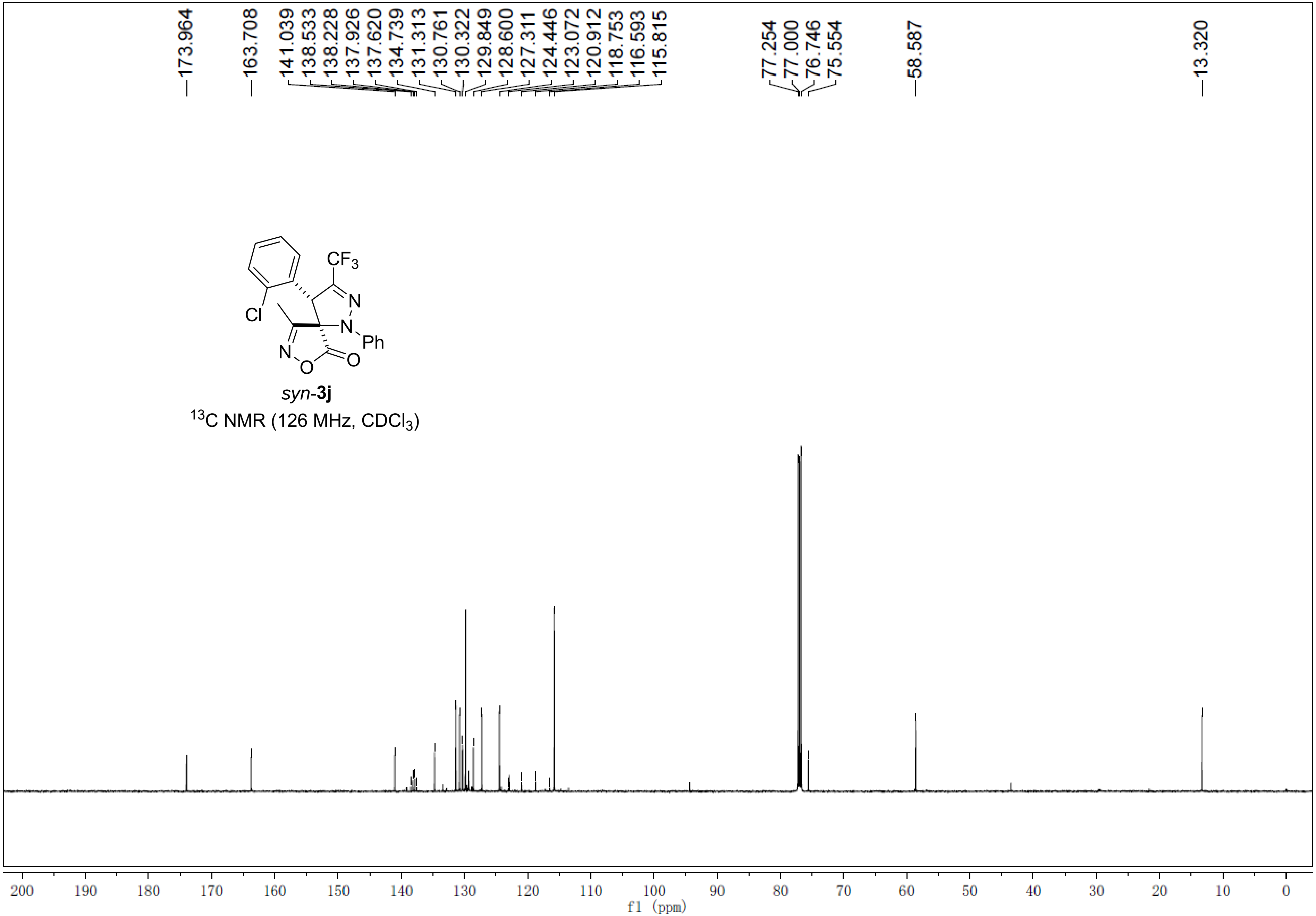
173.964
163.708
141.039
138.533
138.228
137.926
137.620
134.739
131.313
130.761
130.322
129.849
128.600
127.311
124.446
123.072
120.912
118.753
116.593
115.815
77.254
77.000
76.746
75.554
58.587
13.320
CF3
N
N
Ph
Cl
N
O
O
syn-3j
13C NMR (126 MHz, CDCl3)
200 190 180 170 160 150 140 130 120 110 100 90 80 70 60 50 40 30 20 10 0
f1 (ppm)

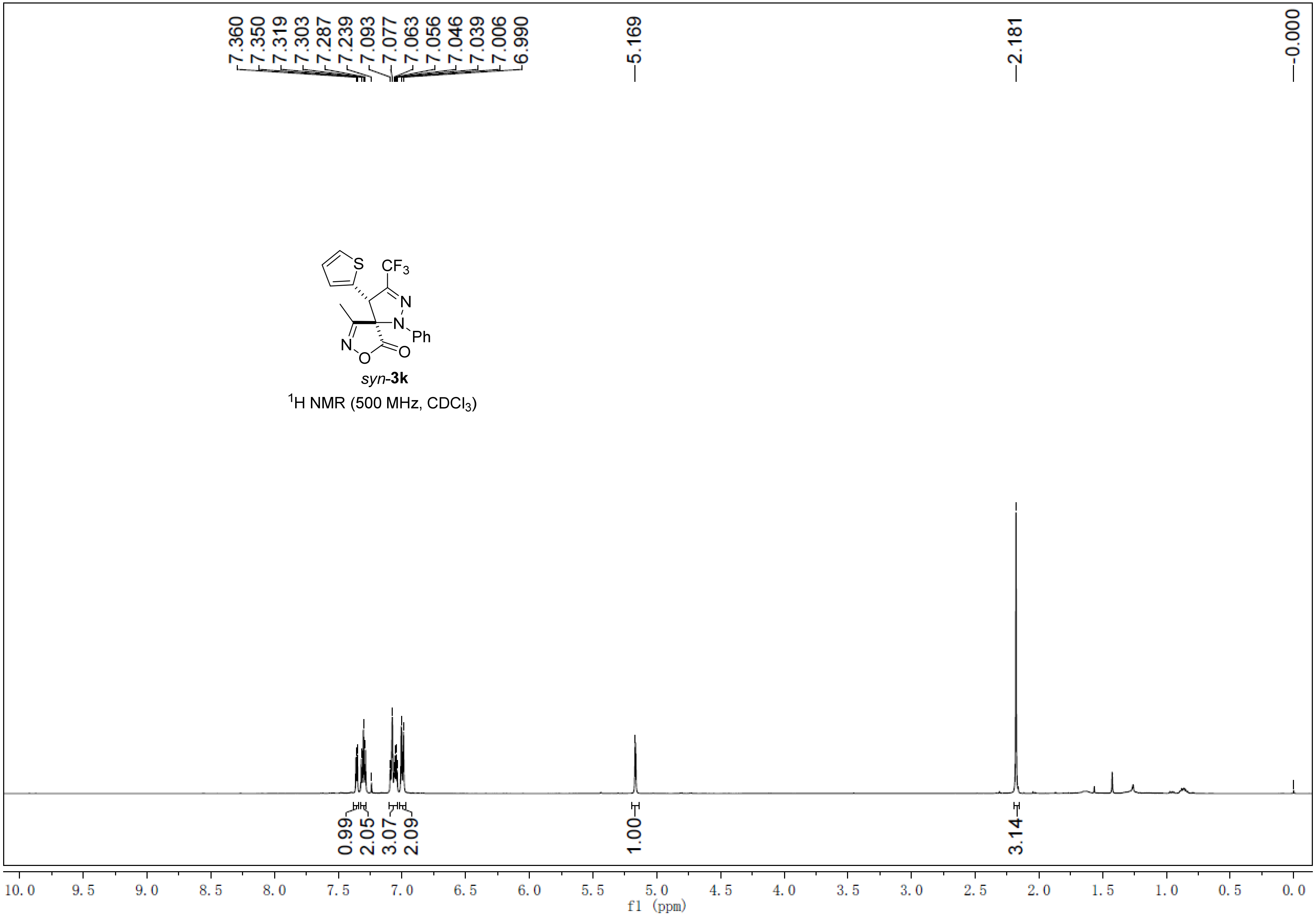

syn-**3k**

^{1}H NMR (500 MHz, $CDCl_3$)

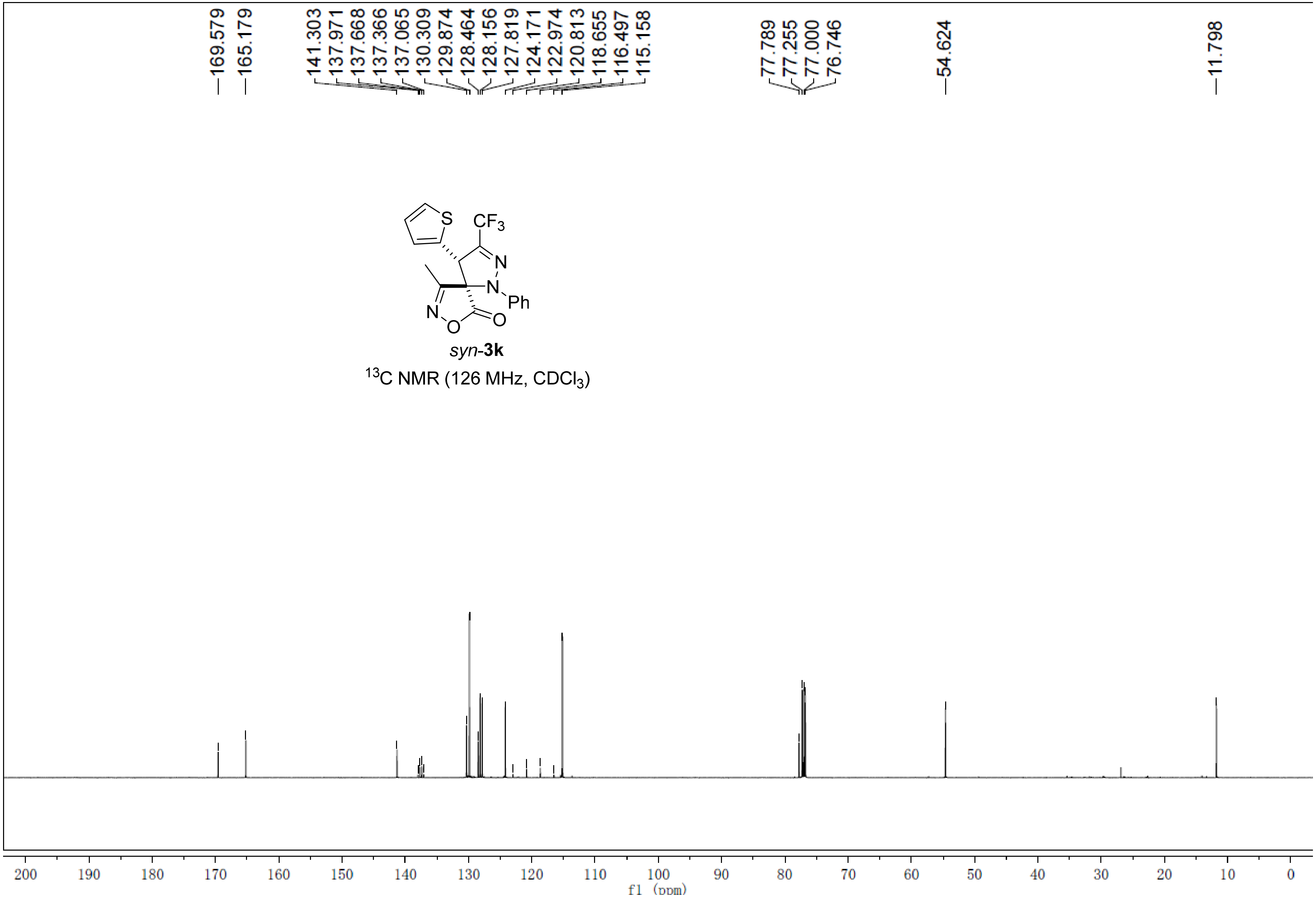

syn-**3k**

^{13}C NMR (126 MHz, $CDCl_3$)

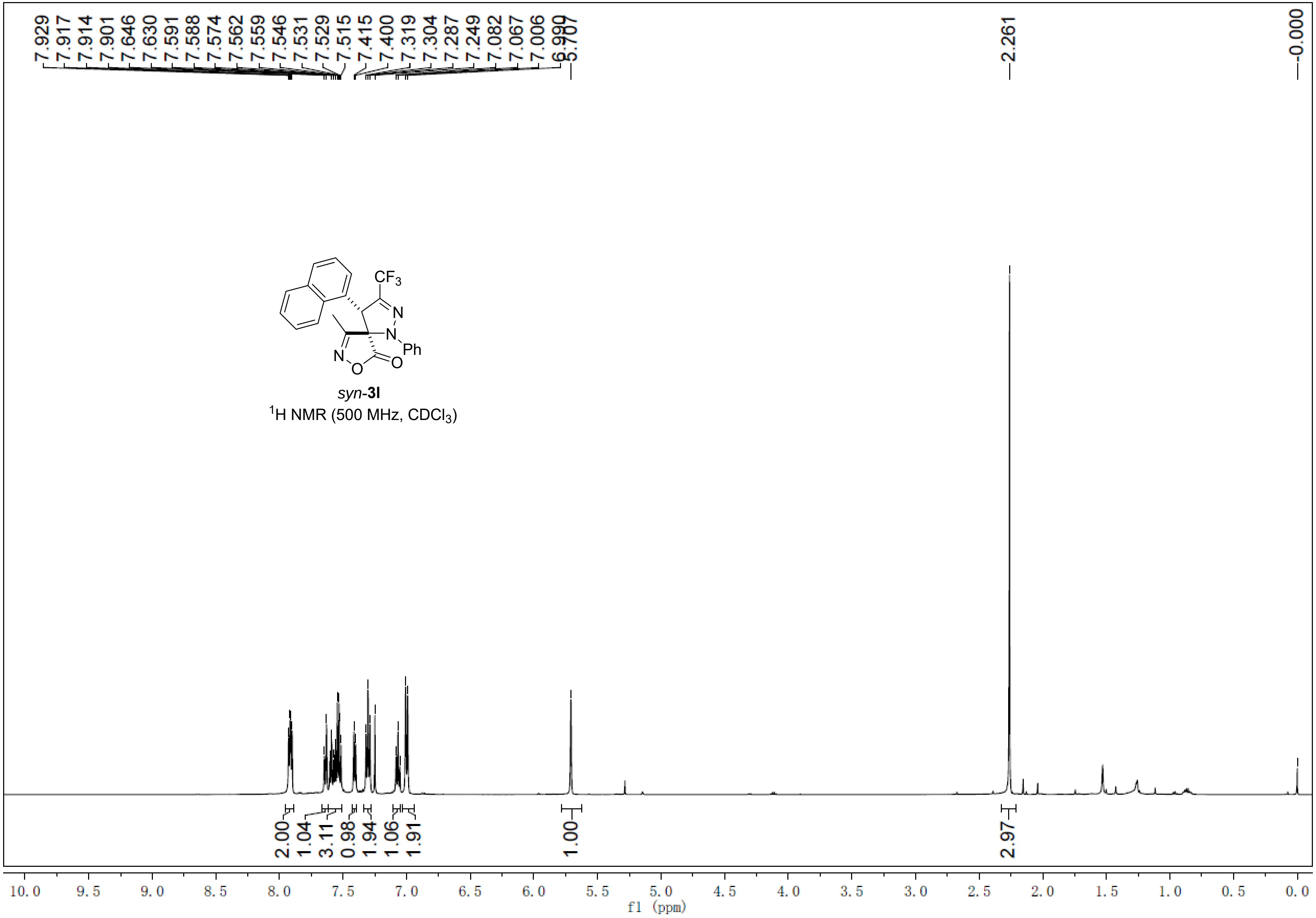
7.929
7.917
7.914
7.901
7.646
7.630
7.591
7.588
7.574
7.562
7.559
7.546
7.531
7.529
7.515
7.415
7.400
7.319
7.304
7.287
7.249
7.082
7.067
7.006
6.990
5.707
2.261
-0.000
CF3
N
N
Ph
N
O
O
syn-3l
1H NMR (500 MHz, CDCl3)
2.00
1.04
3.11
0.98
1.94
1.06
1.91
1.00
2.97
10.0 9.5 9.0 8.5 8.0 7.5 7.0 6.5 6.0 5.5 5.0 4.5 4.0 3.5 3.0 2.5 2.0 1.5 1.0 0.5 0.0
f1 (ppm)

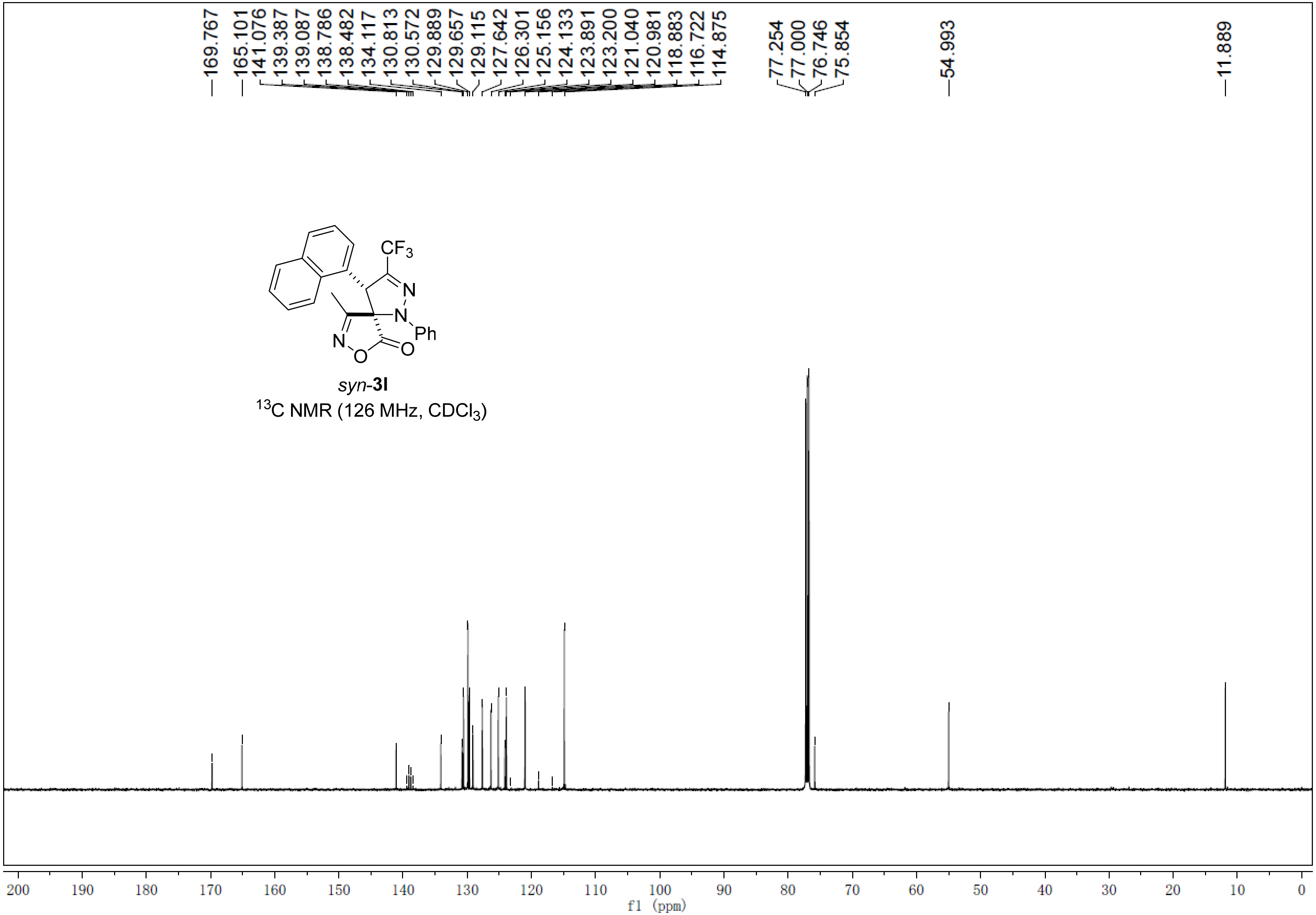

syn-**3l**

^{13}C NMR (126 MHz, $CDCl_3$)

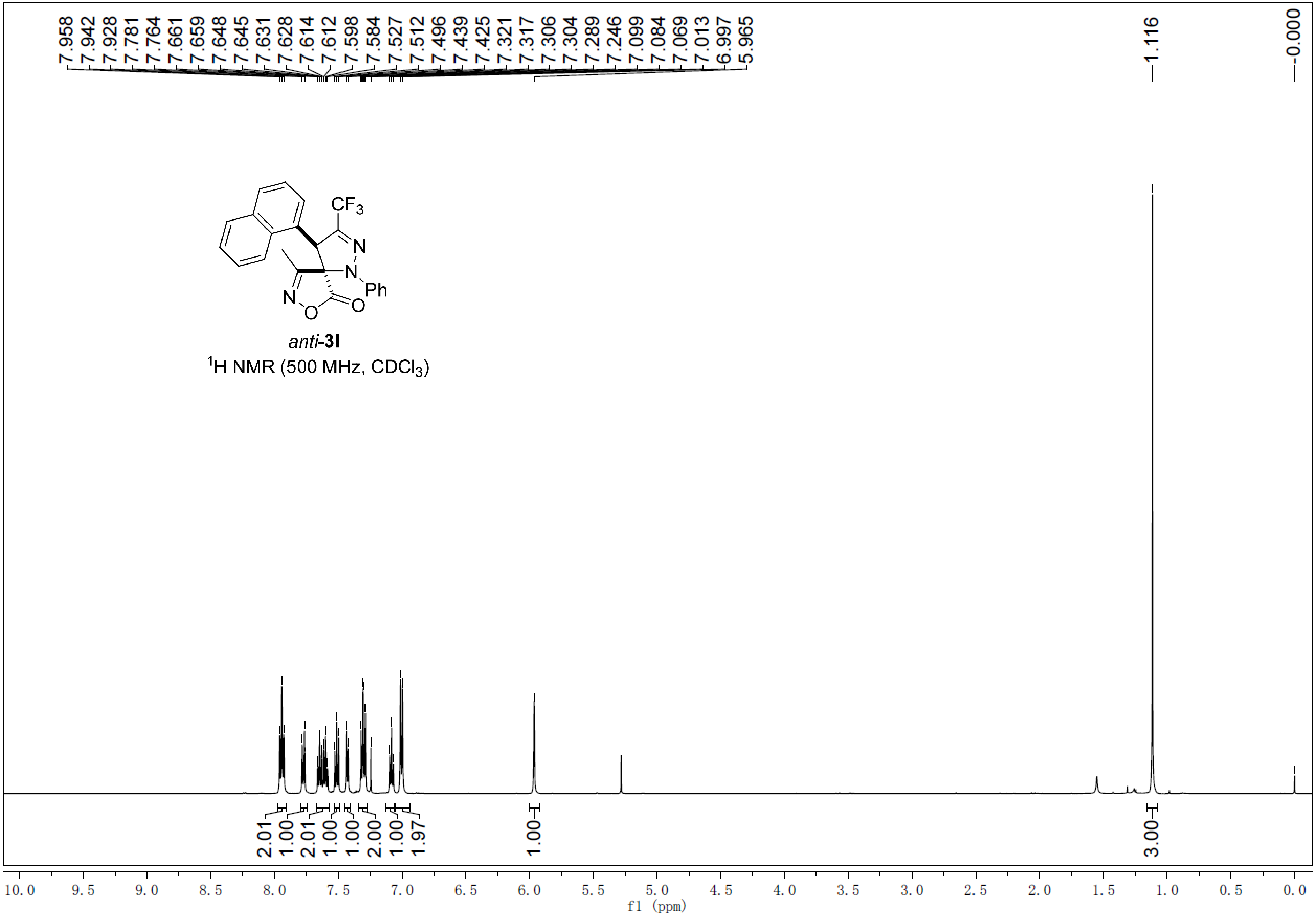
7.958
7.942
7.928
7.781
7.764
7.661
7.659
7.648
7.645
7.631
7.628
7.614
7.612
7.598
7.584
7.527
7.512
7.496
7.439
7.425
7.321
7.317
7.306
7.304
7.289
7.246
7.099
7.084
7.069
7.013
6.997
5.965
1.116
-0.000
CF3
N
N
Ph
N
O
O
anti-3l
1H NMR (500 MHz, CDCl3)
2.01
1.00
2.01
1.00
1.00
2.00
1.00
1.97
1.00
3.00
10.0
9.5
9.0
8.5
8.0
7.5
7.0
6.5
6.0
5.5
5.0
4.5
4.0
3.5
3.0
2.5
2.0
1.5
1.0
0.5
0.0
f1 (ppm)

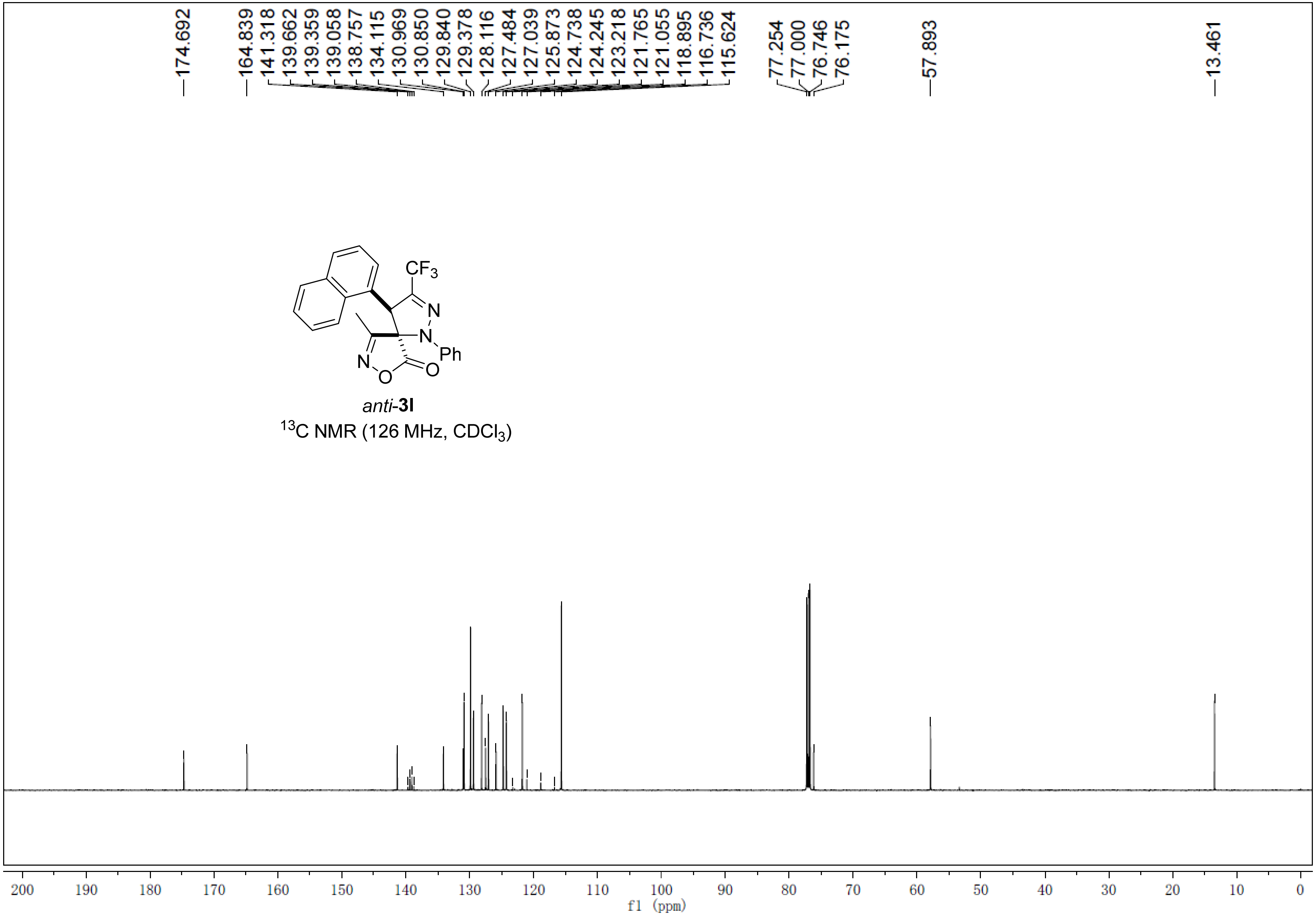
174.692
164.839
141.318
139.662
139.359
139.058
138.757
134.115
130.969
130.850
129.840
129.378
128.116
127.484
127.039
125.873
124.738
124.245
123.218
121.765
121.055
118.895
116.736
115.624
77.254
77.000
76.746
76.175
57.893
13.461
CF3
N
N
Ph
N
O
O
anti-3l
13C NMR (126 MHz, CDCl3)
200 190 180 170 160 150 140 130 120 110 100 90 80 70 60 50 40 30 20 10 0
f1 (ppm)

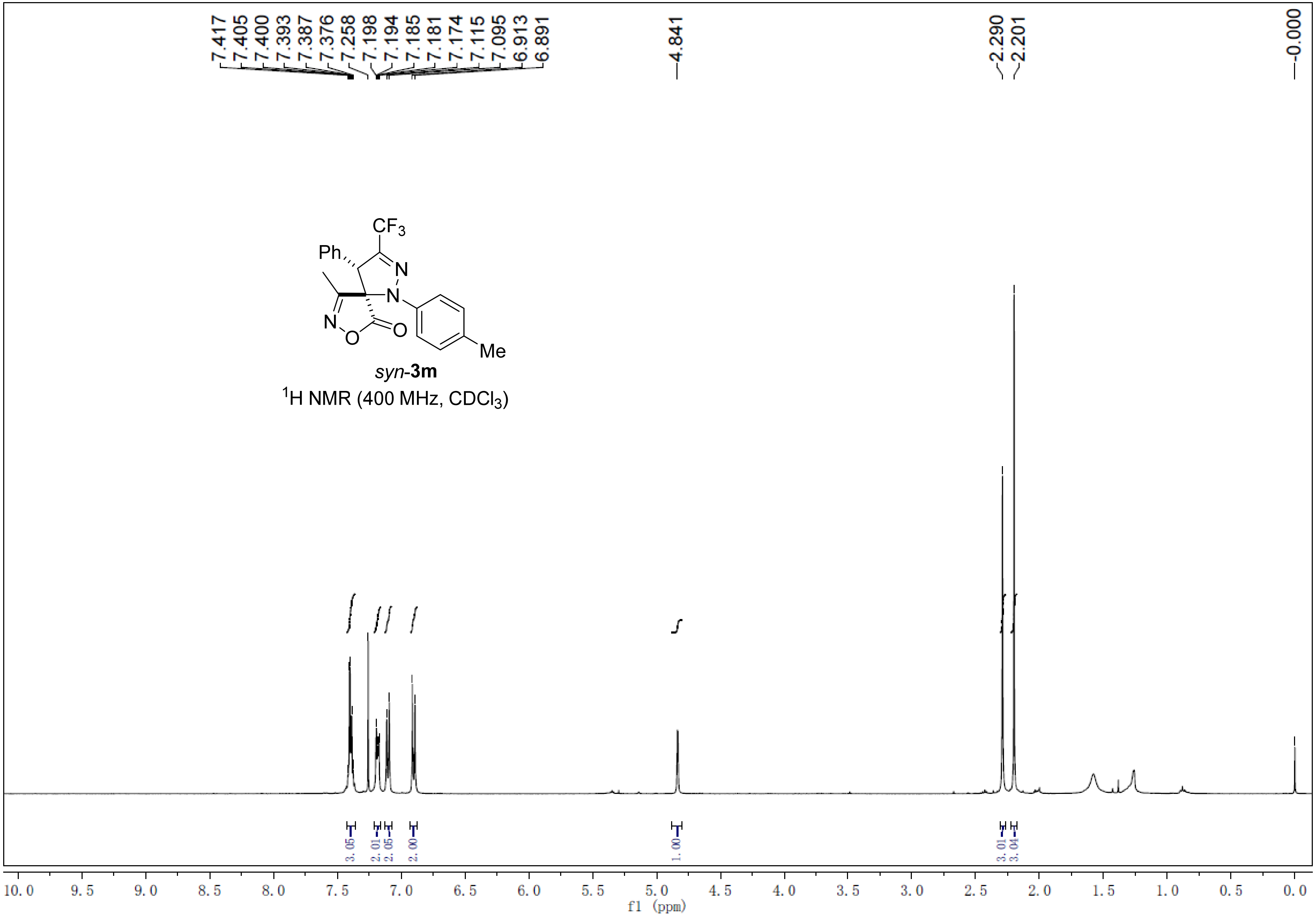
7.417
7.405
7.400
7.393
7.387
7.376
7.258
7.198
7.194
7.185
7.181
7.174
7.115
7.095
6.913
6.891
4.841
2.290
2.201
0.000
CF3
Ph
N
N
N
O
O
Me
syn-3m
1H NMR (400 MHz, CDCl3)
3.05
2.01
2.05
2.00
1.00
3.01
3.04
10.0
9.5
9.0
8.5
8.0
7.5
7.0
6.5
6.0
5.5
5.0
4.5
4.0
3.5
3.0
2.5
2.0
1.5
1.0
0.5
0.0
f1 (ppm)

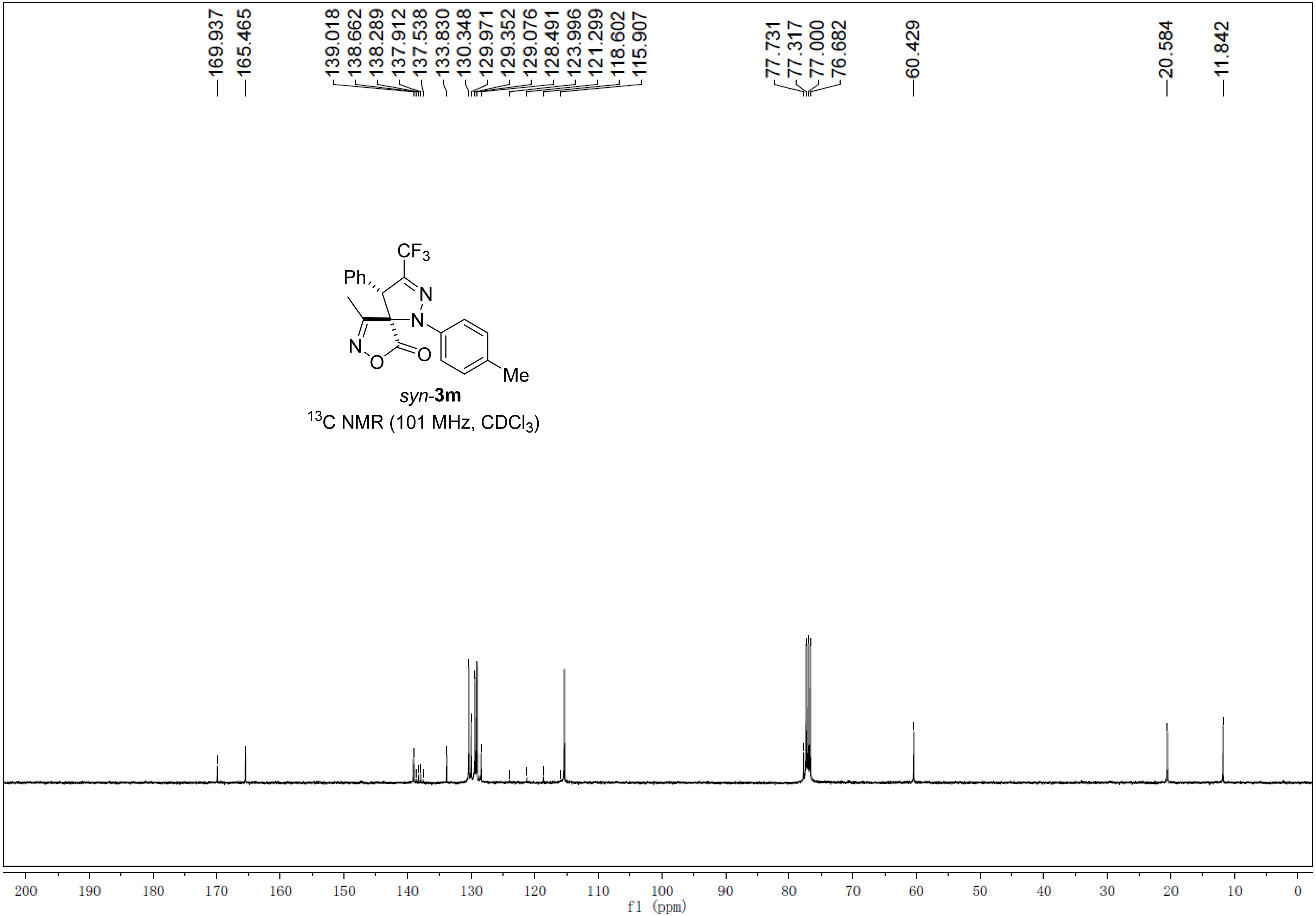

syn-**3m**

^{13}C NMR (101 MHz, $CDCl_3$)

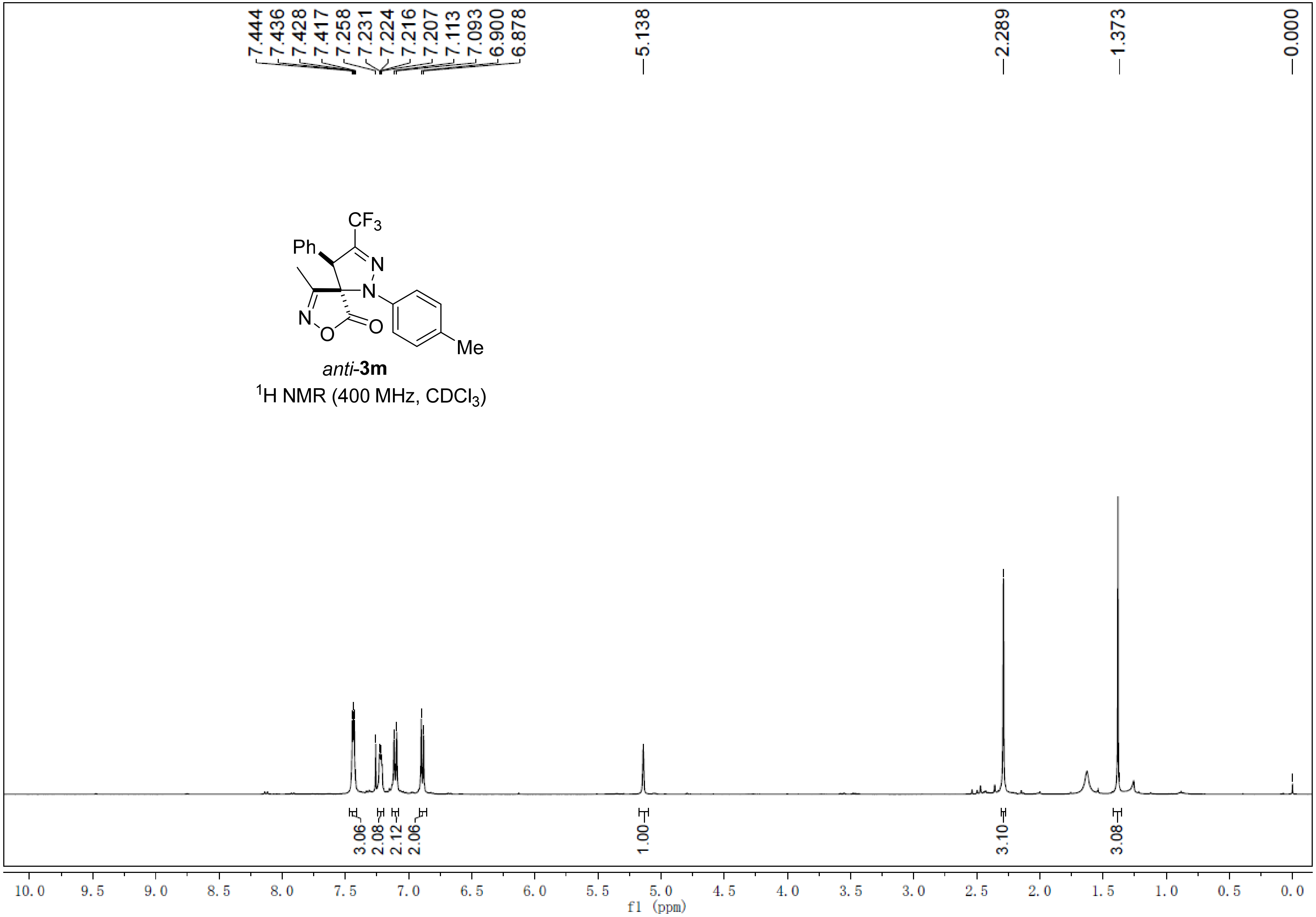

7.444
7.436
7.428
7.417
7.258
7.231
7.224
7.216
7.207
7.113
7.093
6.900
6.878
5.138
2.289
1.373
0.000
CF3
Ph
N
N
N
O
O
Me
anti-3m
1H NMR (400 MHz, CDCl3)
3.06
2.08
2.12
2.06
1.00
3.10
3.08
10.0
9.5
9.0
8.5
8.0
7.5
7.0
6.5
6.0
5.5
5.0
4.5
4.0
3.5
3.0
2.5
2.0
1.5
1.0
0.5
0.0
f1 (ppm)

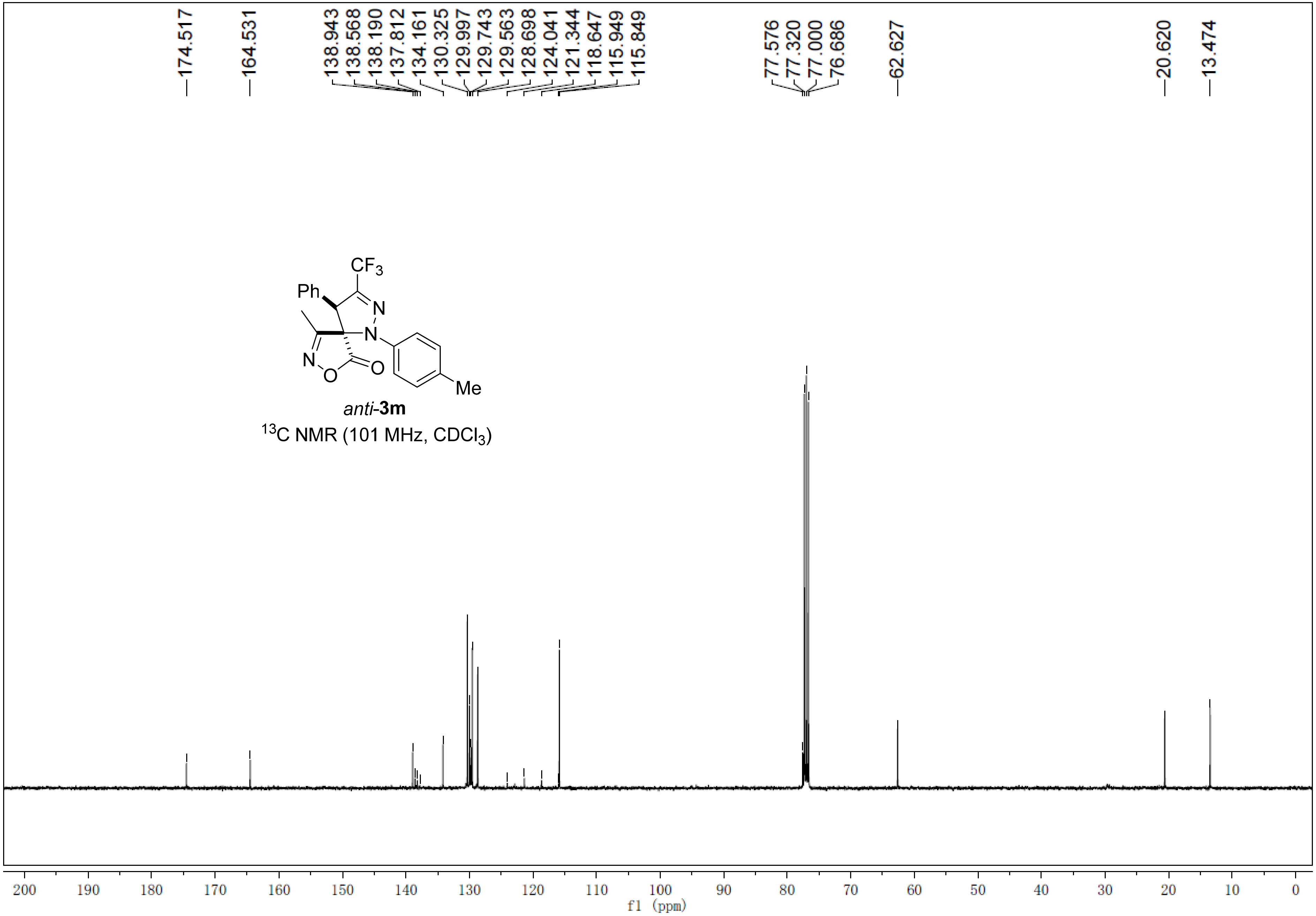
174.517
164.531
138.943
138.568
138.190
137.812
134.161
130.325
129.997
129.743
129.563
128.698
124.041
121.344
118.647
115.949
115.849
77.576
77.320
77.000
76.686
62.627
20.620
13.474
CF3
Ph
N
N
N
O
O
Me
anti-3m
13C NMR (101 MHz, CDCl3)
200
190
180
170
160
150
140
130
120
110
100
90
80
70
60
50
40
30
20
10
0
f1 (ppm)

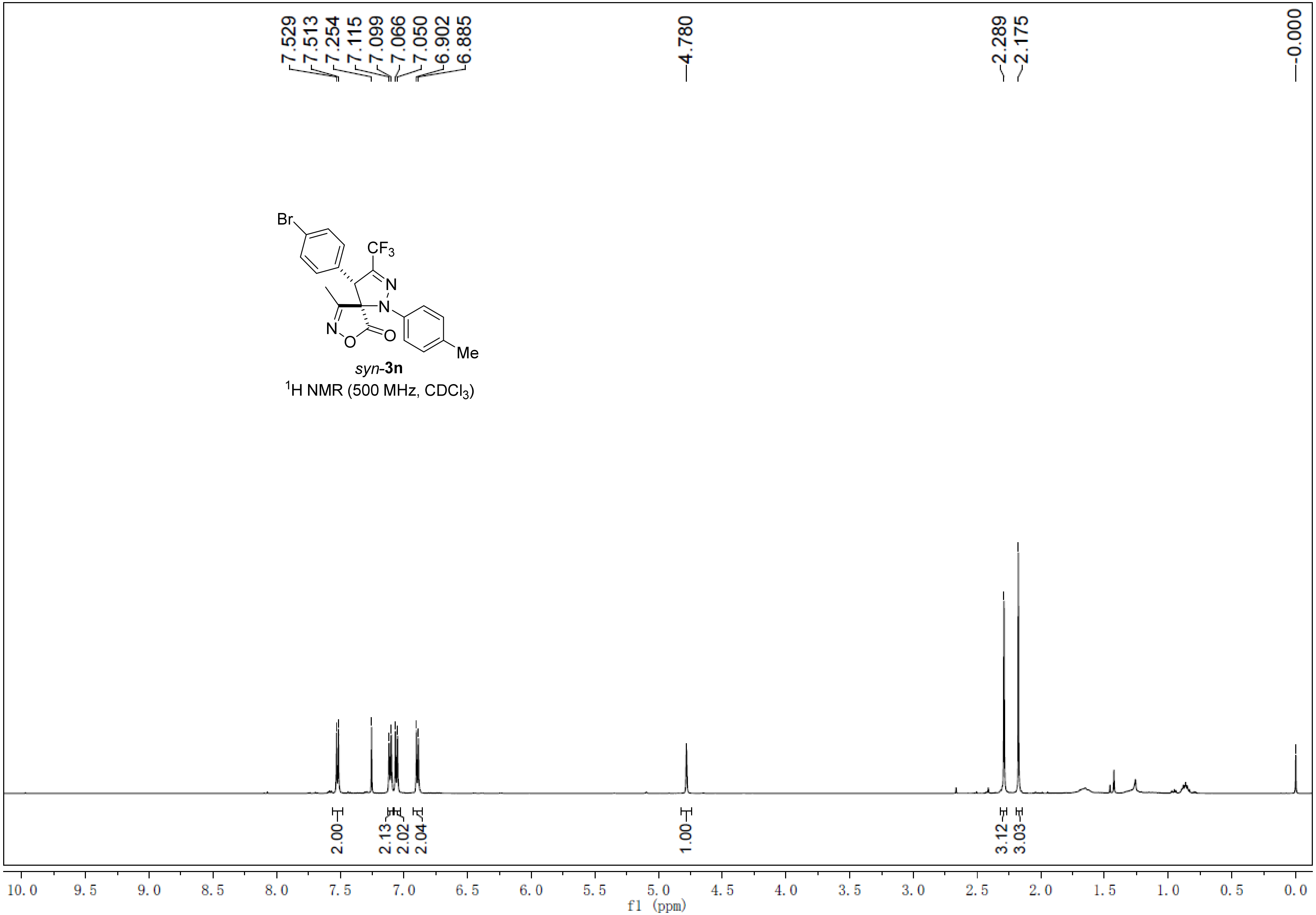
7.529
7.513
7.254
7.115
7.099
7.066
7.050
6.902
6.885
4.780
2.289
2.175
-0.000
Br
CF3
N
N
N
O
O
Me
syn-3n
1H NMR (500 MHz, CDCl3)
2.00
2.13
2.02
2.04
1.00
3.12
3.03
10.0 9.5 9.0 8.5 8.0 7.5 7.0 6.5 6.0 5.5 5.0 4.5 4.0 3.5 3.0 2.5 2.0 1.5 1.0 0.5 0.0
f1 (ppm)

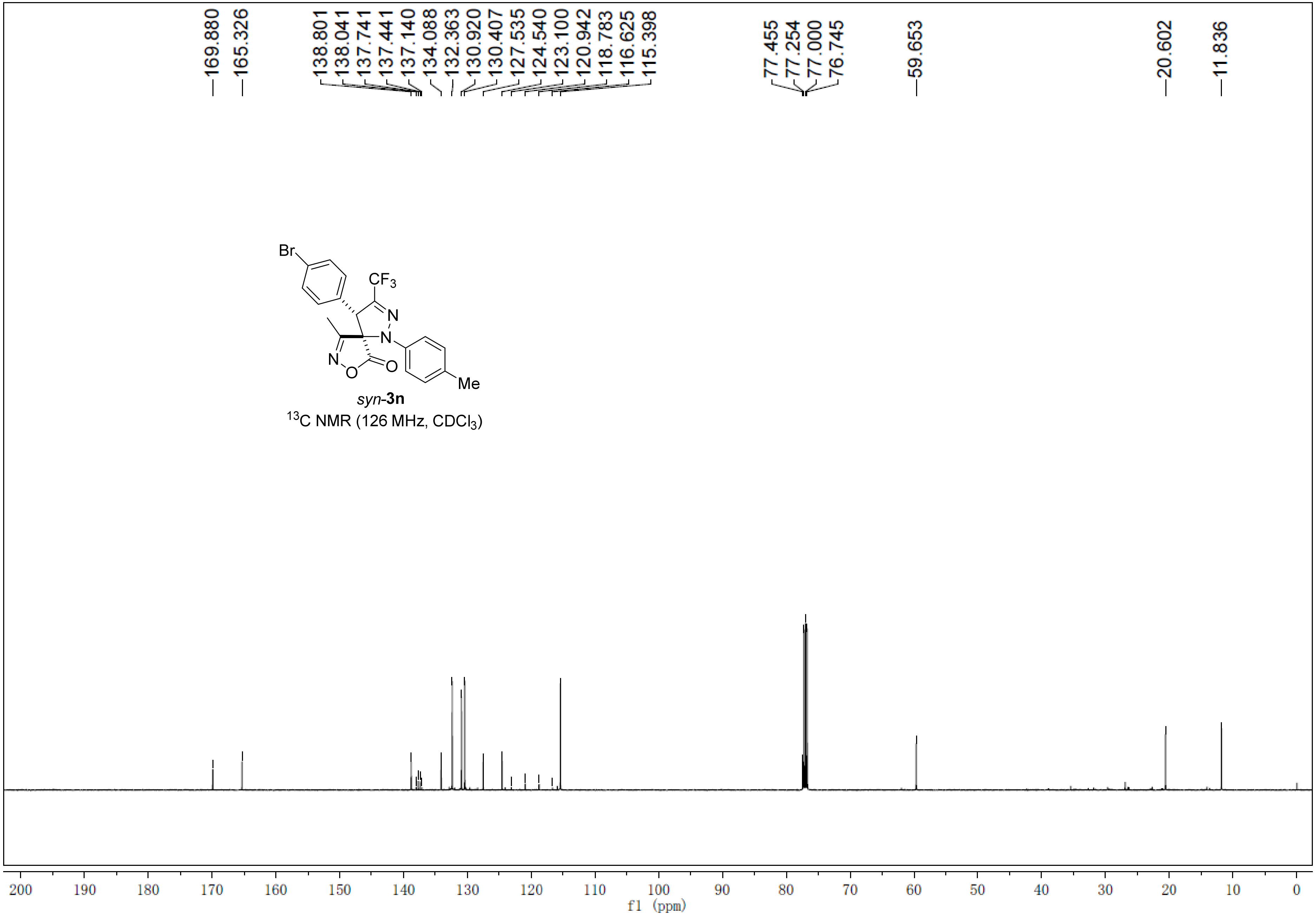

syn-**3n**

^{13}C NMR (126 MHz, $CDCl_3$)

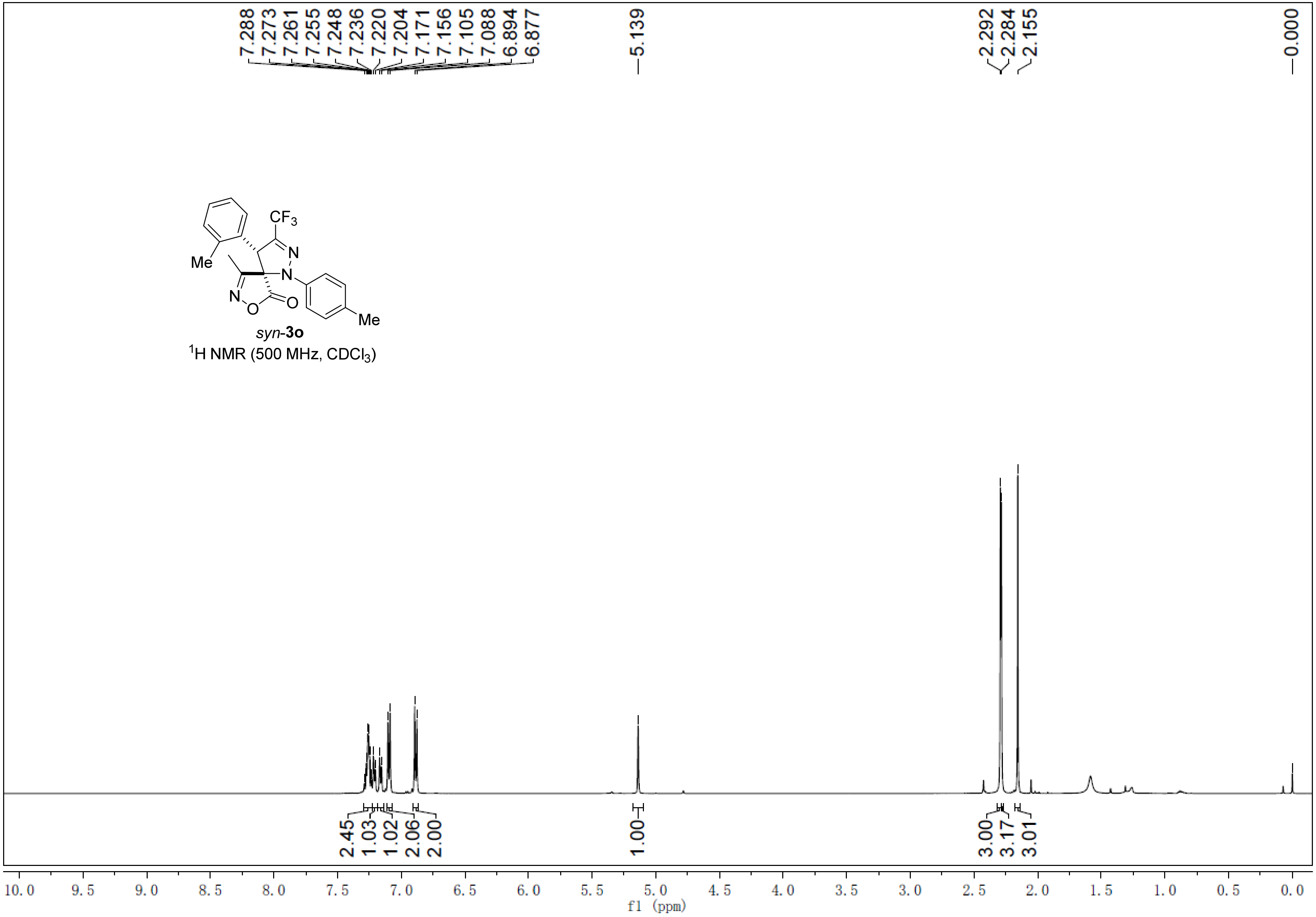
7.288
7.273
7.261
7.255
7.248
7.236
7.220
7.204
7.171
7.156
7.105
7.088
6.894
6.877
5.139
2.292
2.284
2.155
0.000
CF3
N
N
Me
N
O
O
Me
syn-3o
1H NMR (500 MHz, CDCl3)
2.45
1.03
1.02
2.06
2.00
1.00
3.00
3.17
3.01
10.0
9.5
9.0
8.5
8.0
7.5
7.0
6.5
6.0
5.5
5.0
4.5
4.0
3.5
3.0
2.5
2.0
1.5
1.0
0.5
0.0
f1 (ppm)

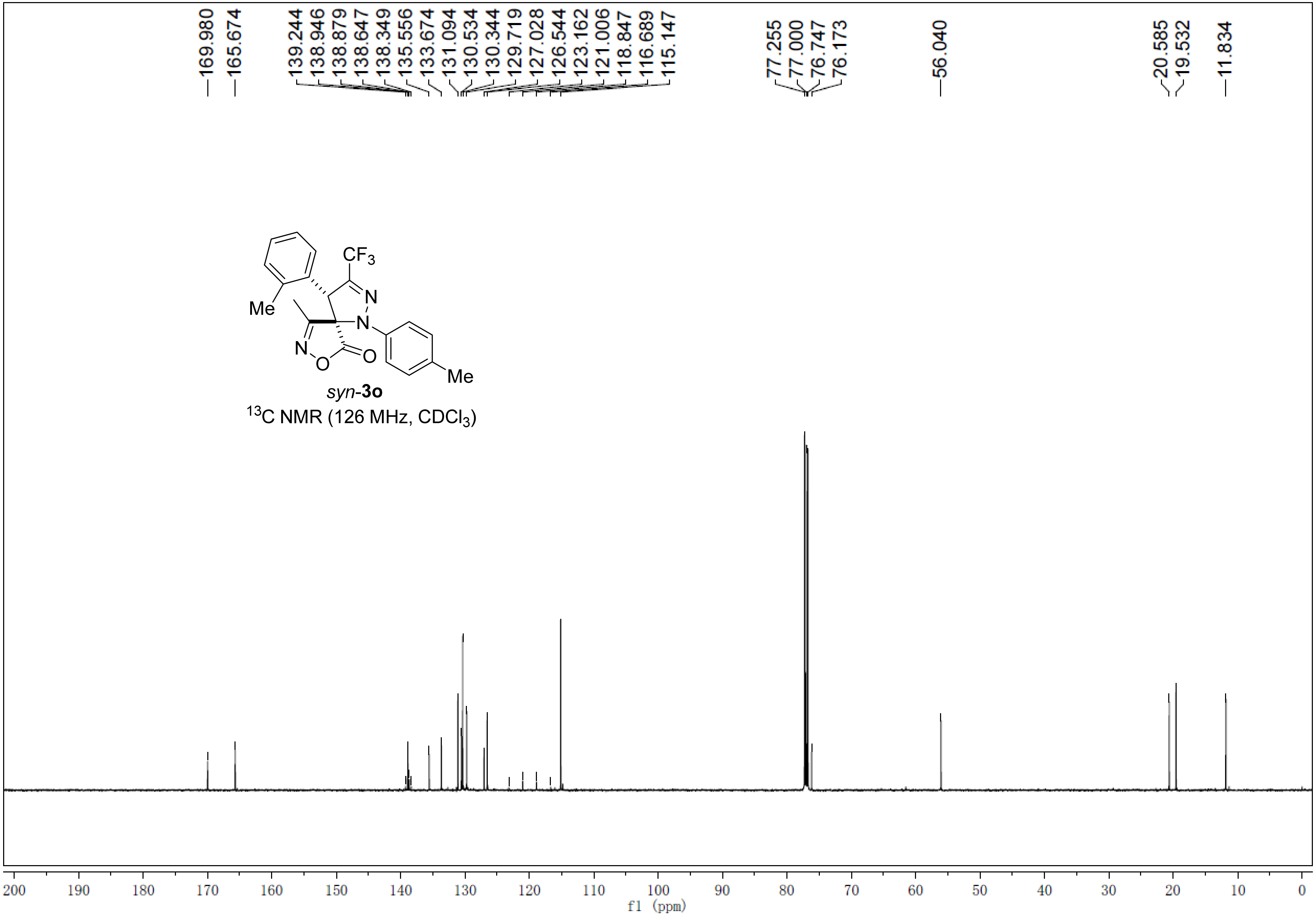

169.980
165.674
139.244
138.946
138.879
138.647
138.349
135.556
133.674
131.094
130.534
130.344
129.719
127.028
126.544
123.162
121.006
118.847
116.689
115.147
77.255
77.000
76.747
76.173
56.040
20.585
19.532
11.834
CF3
N
N
Me
N
O
O
Me
syn-3o
13C NMR (126 MHz, CDCl3)
200
190
180
170
160
150
140
130
120
110
100
90
80
70
60
50
40
30
20
10
0
f1 (ppm)

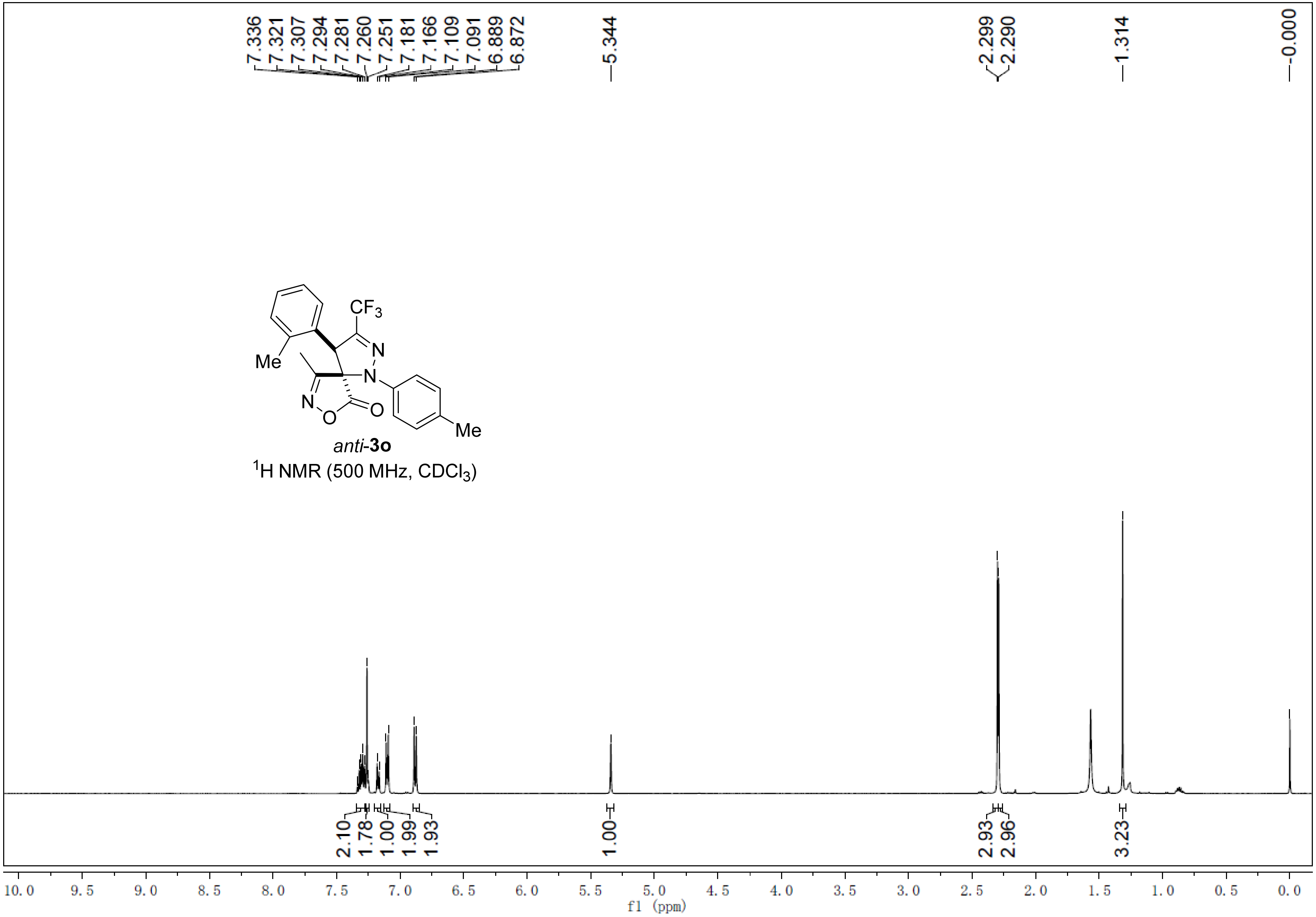

7.336
7.321
7.307
7.294
7.281
7.260
7.251
7.181
7.166
7.109
7.091
6.889
6.872
5.344
2.299
2.290
1.314
-0.000
CF3
N
N
Me
Me
N
O
O
anti-3o
1H NMR (500 MHz, CDCl3)
2.10
1.78
1.00
1.99
1.93
1.00
2.93
2.96
3.23
10.0
9.5
9.0
8.5
8.0
7.5
7.0
6.5
6.0
5.5
5.0
4.5
4.0
3.5
3.0
2.5
2.0
1.5
1.0
0.5
0.0
f1 (ppm)

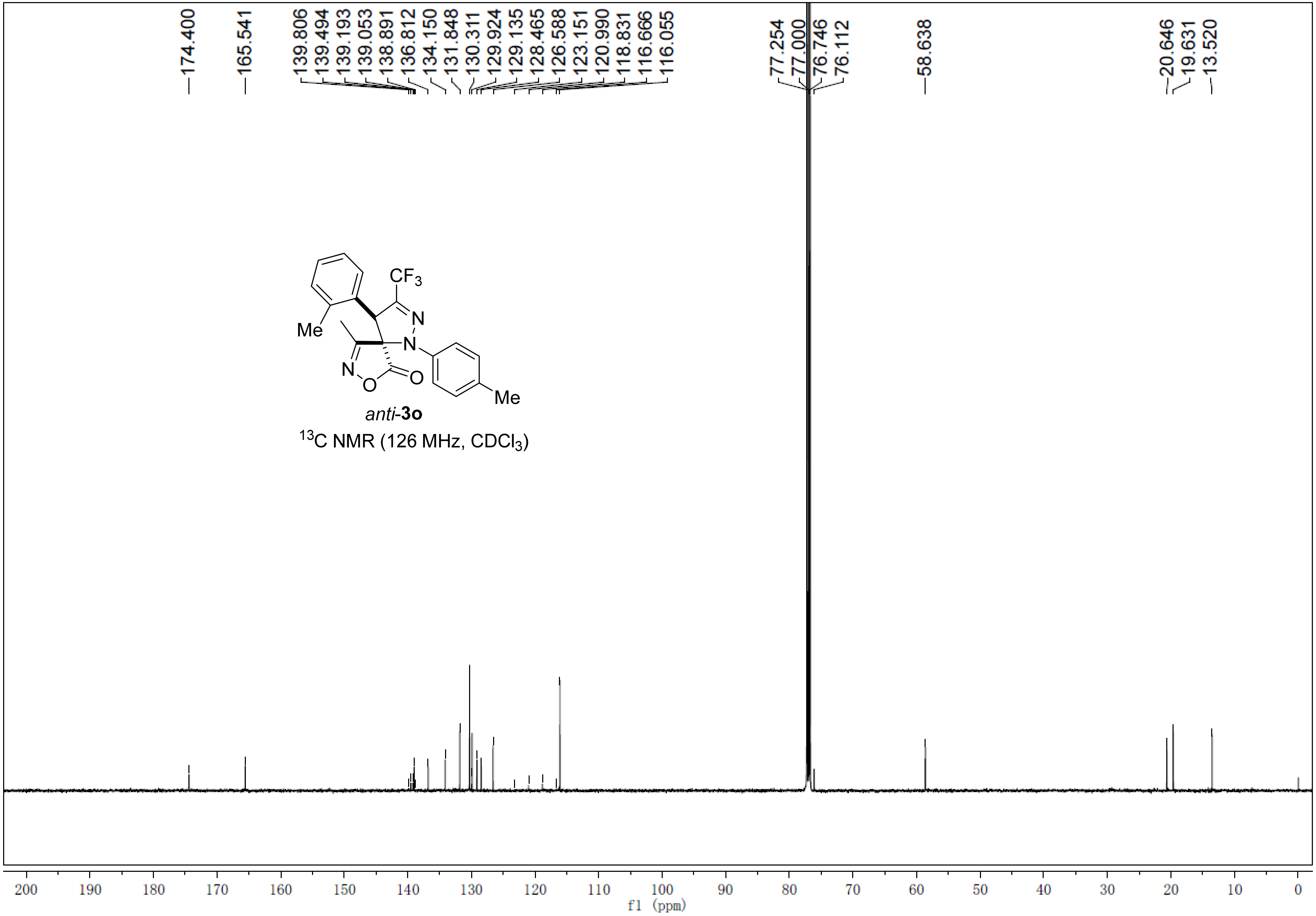

174.400
165.541
139.806
139.494
139.193
139.053
138.891
136.812
134.150
131.848
130.311
129.924
129.135
128.465
126.588
123.151
120.990
118.831
116.666
116.055
77.254
77.000
76.746
76.112
58.638
20.646
19.631
13.520
CF3
N
N
Me
N
O
O
Me
anti-3o
13C NMR (126 MHz, CDCl3)
200 190 180 170 160 150 140 130 120 110 100 90 80 70 60 50 40 30 20 10 0
f1 (ppm)

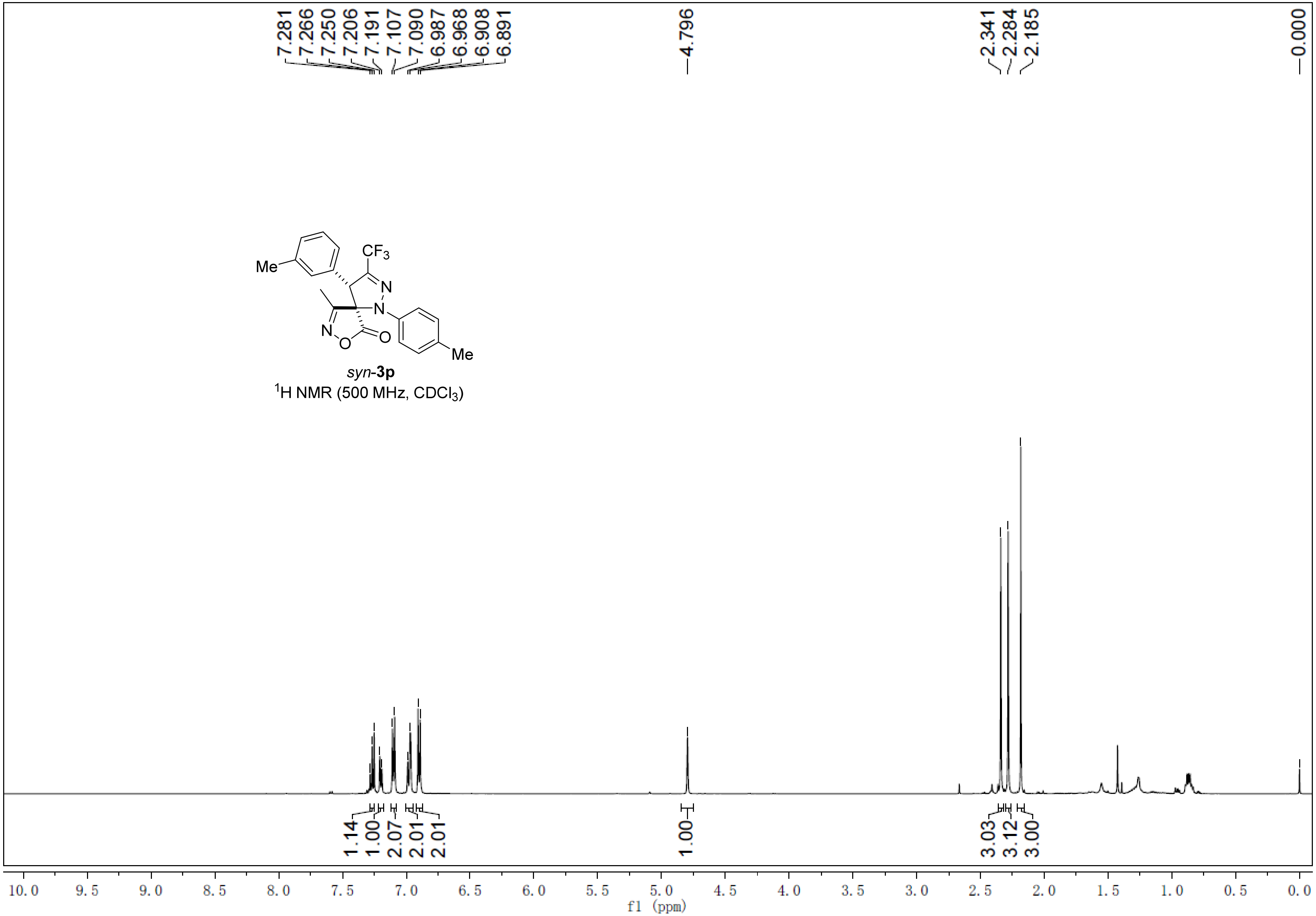

syn-**3p**

^{1}H NMR (500 MHz, $CDCl_3$)

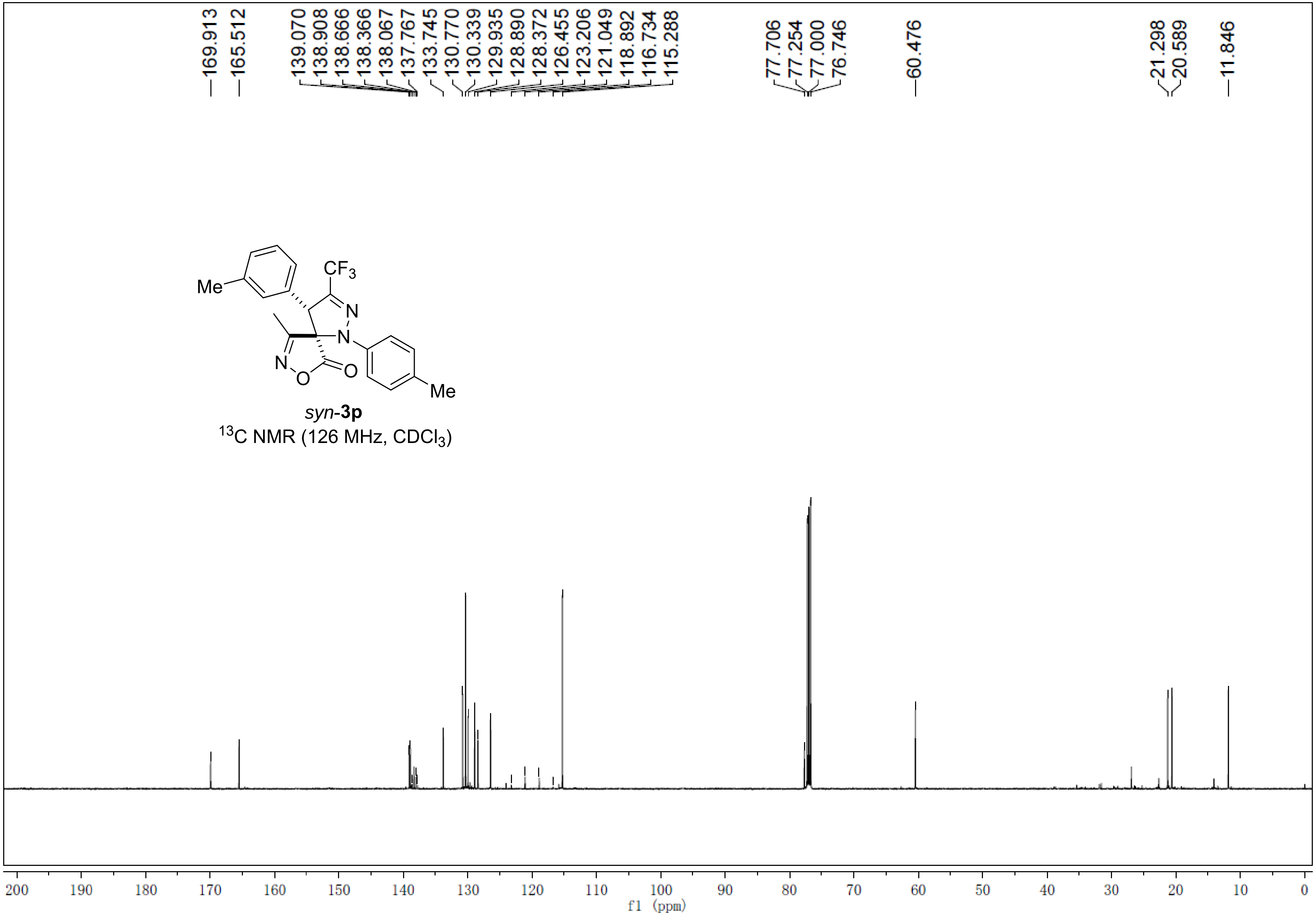

syn-**3p**
^{13}C NMR (126 MHz, $CDCl_3$)

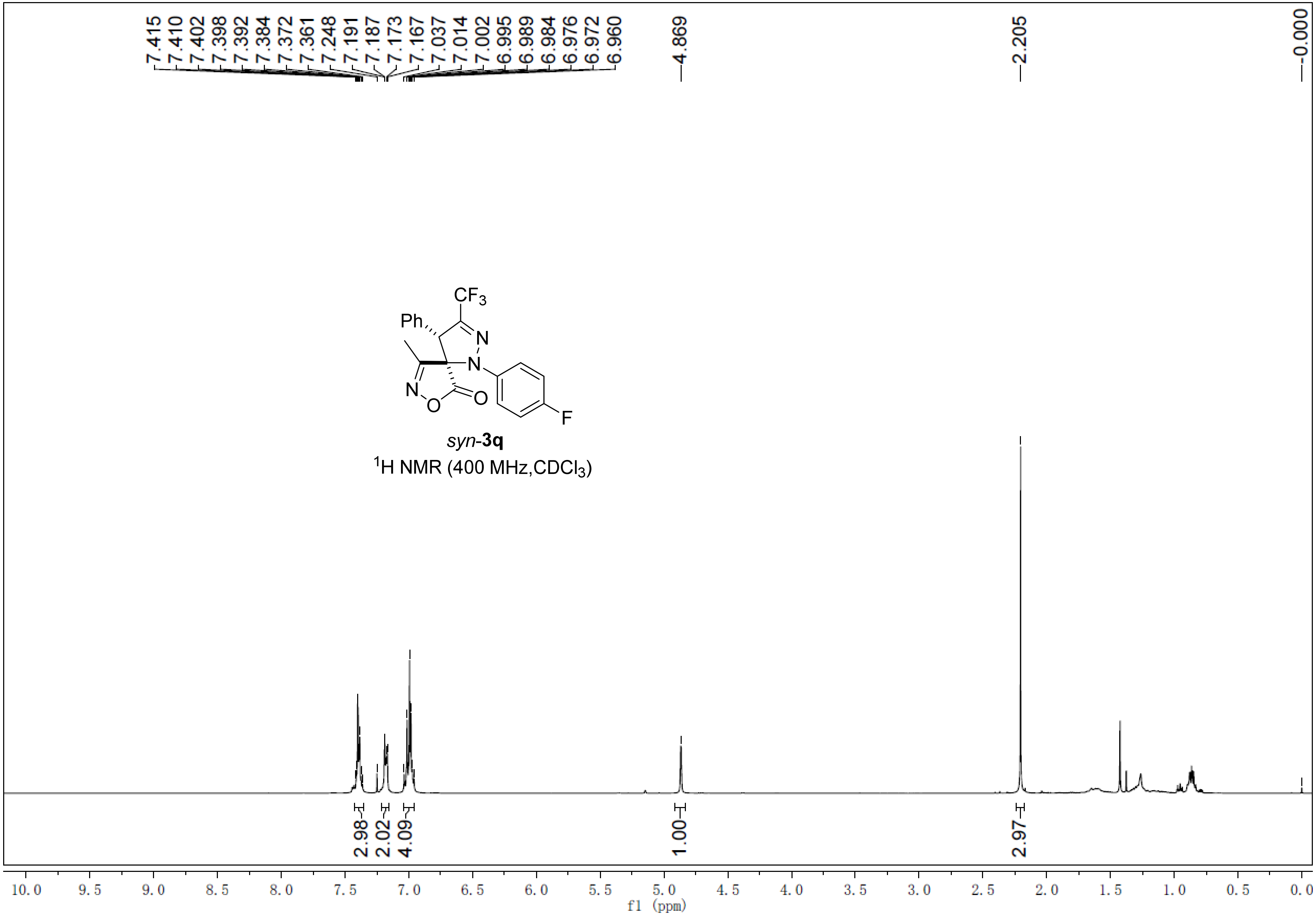
7.415
7.410
7.402
7.398
7.392
7.384
7.372
7.361
7.248
7.191
7.187
7.173
7.167
7.037
7.014
7.002
6.995
6.989
6.984
6.976
6.972
6.960
4.869
2.205
-0.000
CF3
Ph
N
N
N
O
O
F
syn-3q
1H NMR (400 MHz,CDCl3)
2.98
2.02
4.09
1.00
2.97
f1 (ppm)

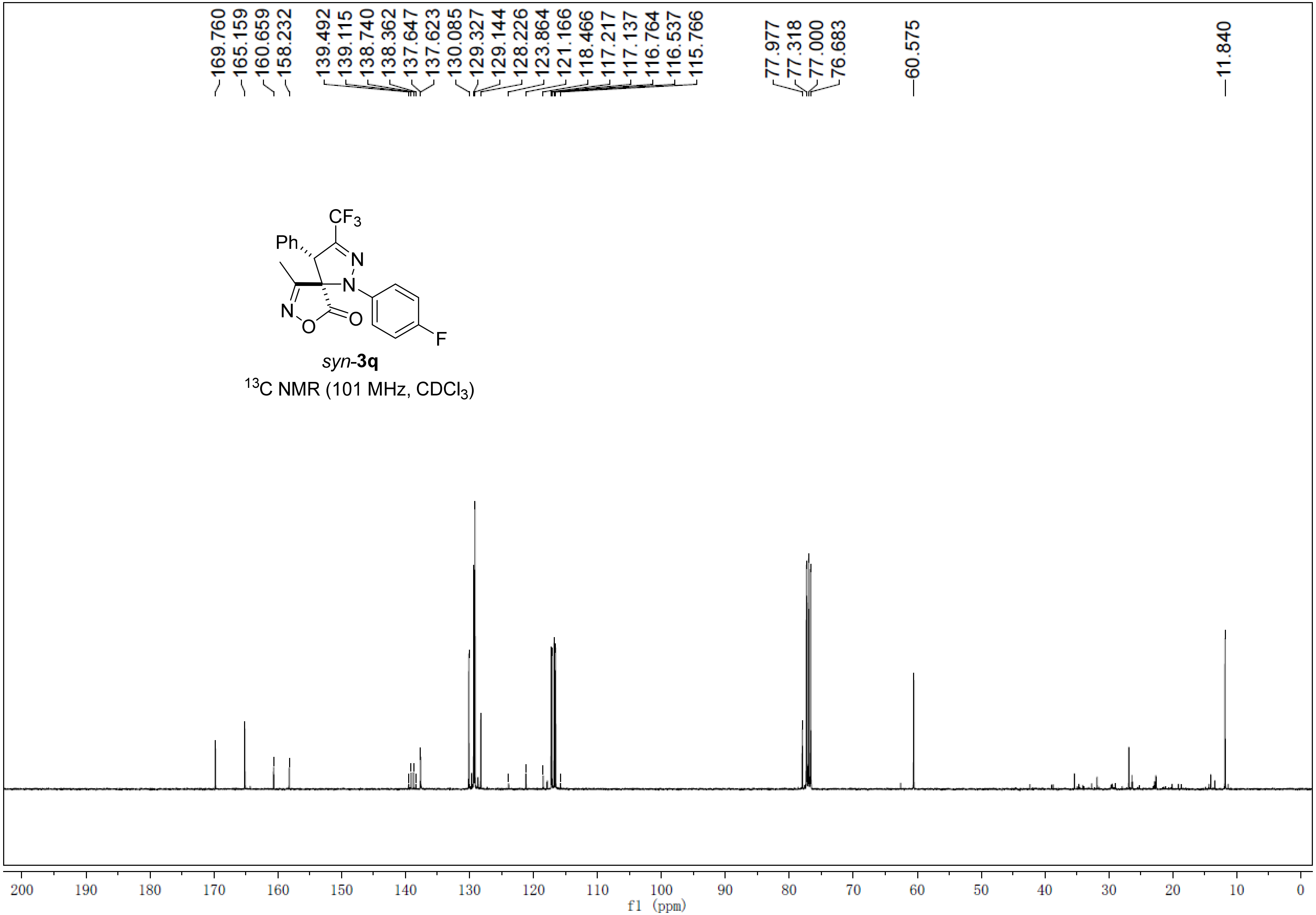
169.760
165.159
160.659
158.232
139.492
139.115
138.740
138.362
137.647
137.623
130.085
129.327
129.144
128.226
123.864
121.166
118.466
117.217
117.137
116.764
116.537
115.766
77.977
77.318
77.000
76.683
60.575
11.840
CF3
Ph
N
N
N
O
O
F
syn-3q
13C NMR (101 MHz, CDCl3)
200
190
180
170
160
150
140
130
120
110
100
90
80
70
60
50
40
30
20
10
0
f1 (ppm)

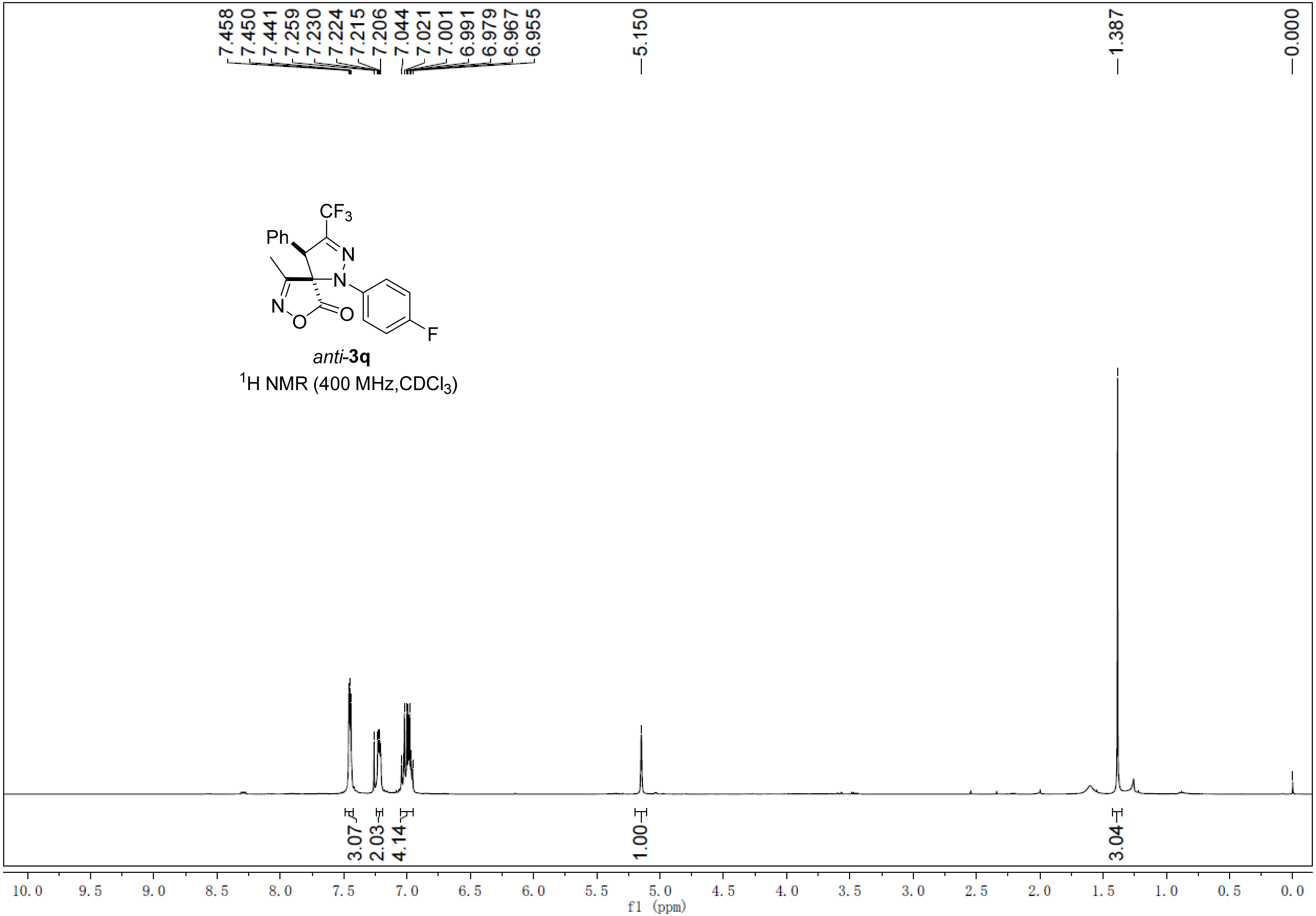

7.458
7.450
7.441
7.259
7.230
7.224
7.215
7.206
7.044
7.021
7.001
6.991
6.979
6.967
6.955
5.150
1.387
0.000
CF3
Ph
N
N
N
O
O
F
anti-3q
1H NMR (400 MHz,CDCl3)
3.07
2.03
4.14
1.00
3.04
10.0
9.5
9.0
8.5
8.0
7.5
7.0
6.5
6.0
5.5
5.0
4.5
4.0
3.5
3.0
2.5
2.0
1.5
1.0
0.5
0.0
f1 (ppm)

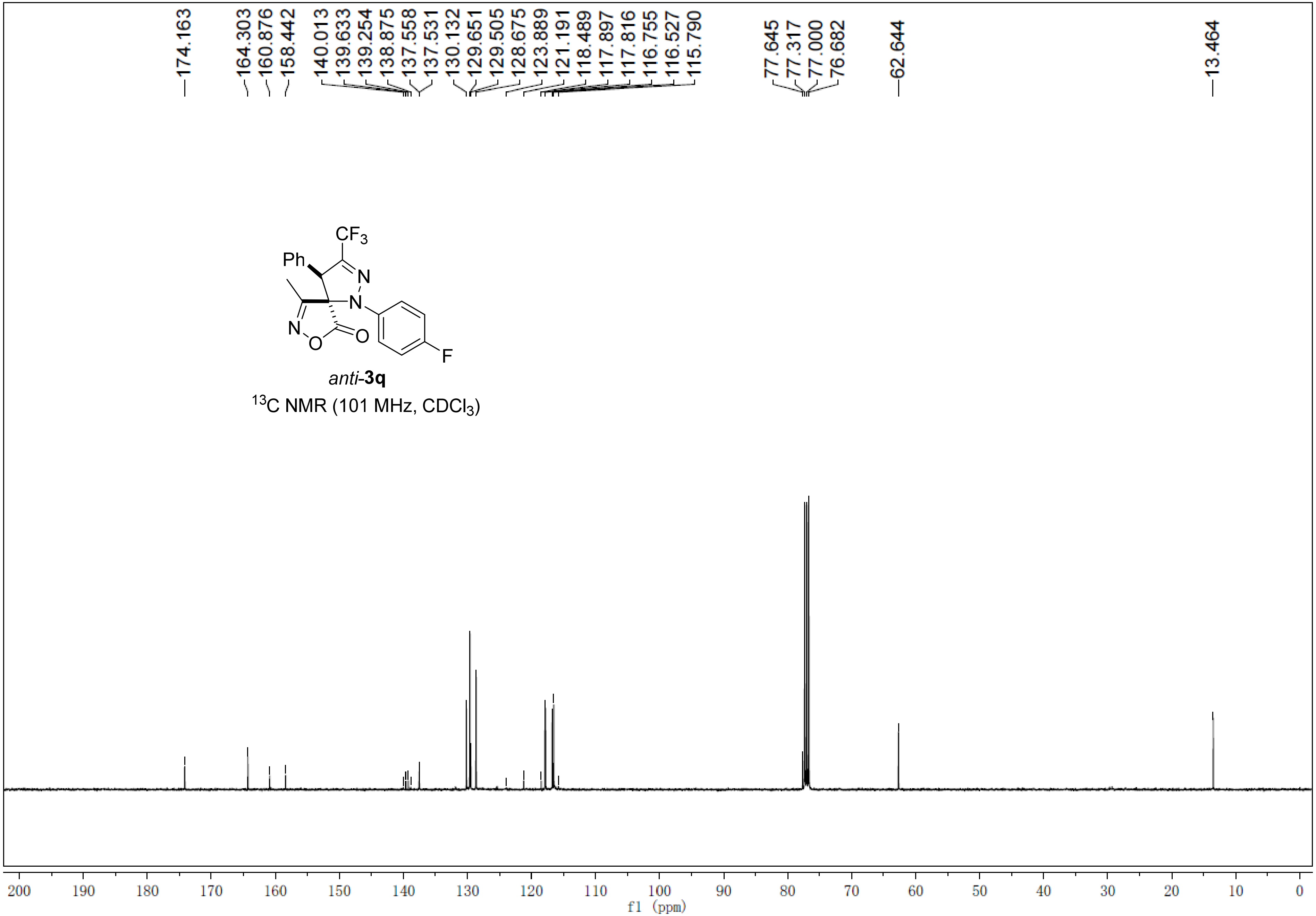

anti-**3q**

^{13}C NMR (101 MHz, $CDCl_3$)

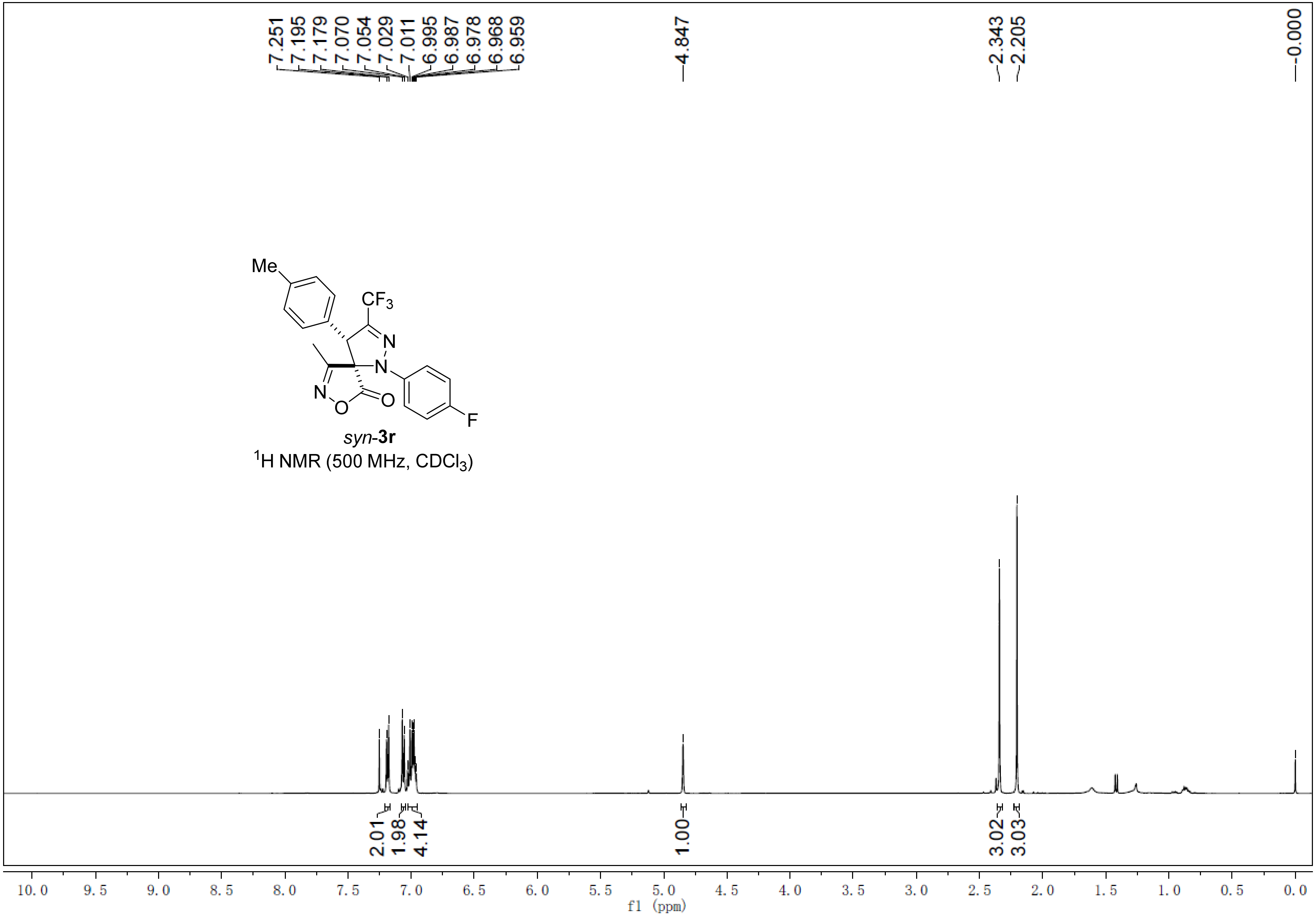
7.251
7.195
7.179
7.070
7.054
7.029
7.011
6.995
6.987
6.978
6.968
6.959
4.847
2.343
2.205
-0.000
Me
CF_3
N
N
N
O
O
F
syn-3r
^{1}H NMR (500 MHz, $CDCl_3$)
2.01
1.98
4.14
1.00
3.02
3.03
10.0
9.5
9.0
8.5
8.0
7.5
7.0
6.5
6.0
5.5
5.0
4.5
4.0
3.5
3.0
2.5
2.0
1.5
1.0
0.5
0.0
f1 (ppm)

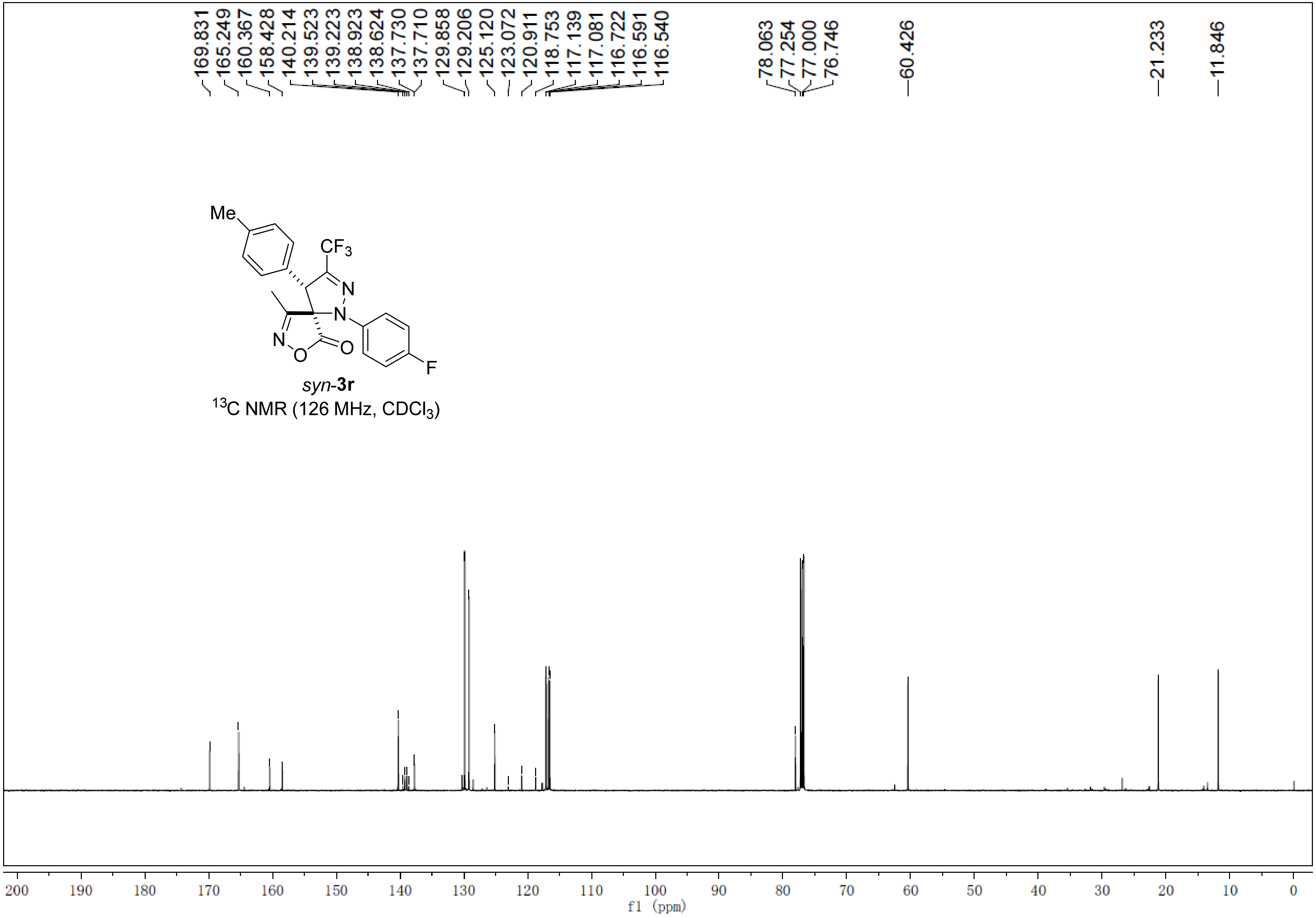

syn-**3r**

^{13}C NMR (126 MHz, $CDCl_3$)

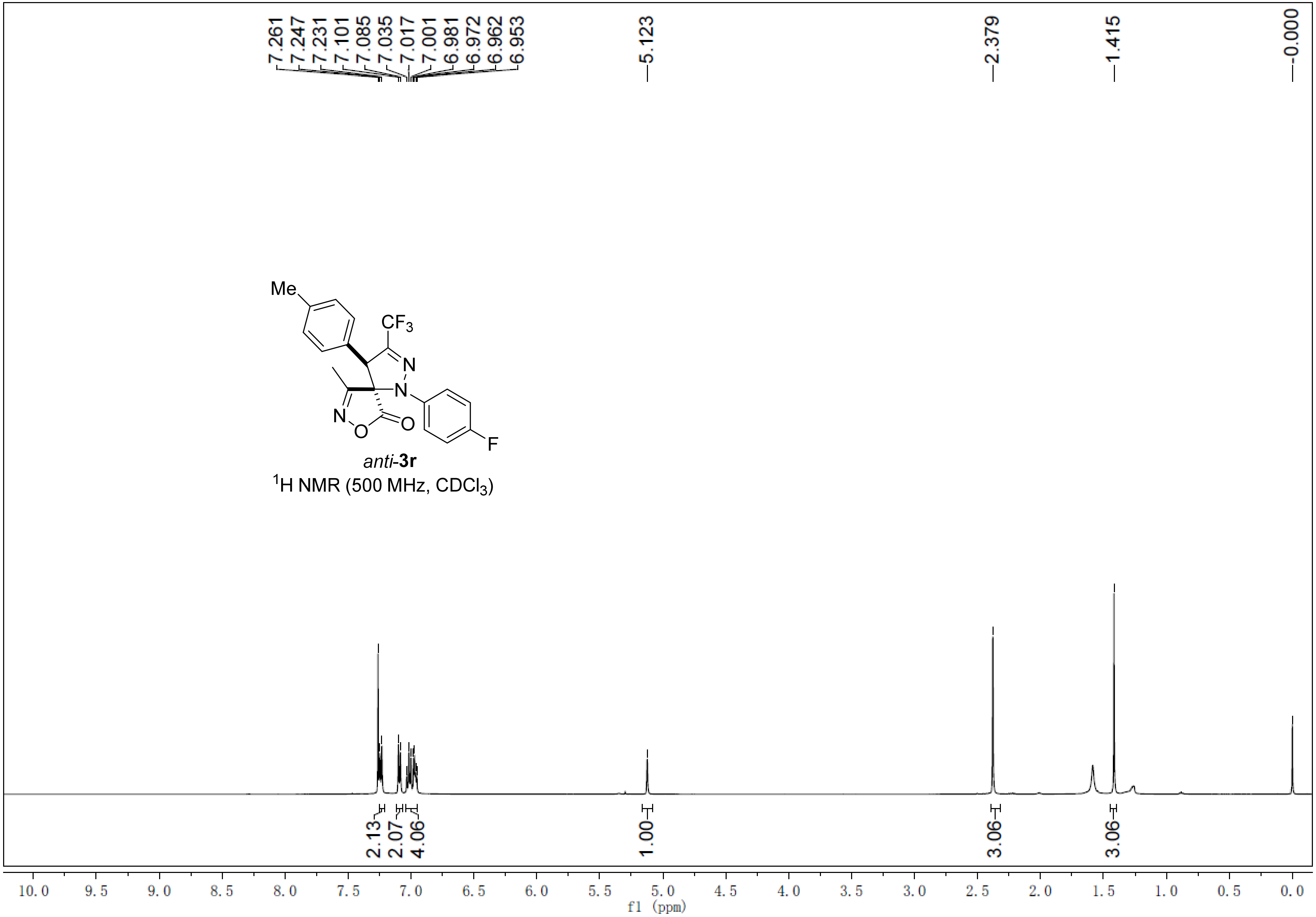
7.261
7.247
7.231
7.101
7.085
7.035
7.017
7.001
6.981
6.972
6.962
6.953
5.123
2.379
1.415
-0.000
Me
CF3
N
N
N
O
O
F
anti-3r
1H NMR (500 MHz, CDCl3)
2.13
2.07
4.06
1.00
3.06
3.06
10.0
9.5
9.0
8.5
8.0
7.5
7.0
6.5
6.0
5.5
5.0
4.5
4.0
3.5
3.0
2.5
2.0
1.5
1.0
0.5
0.0
f1 (ppm)

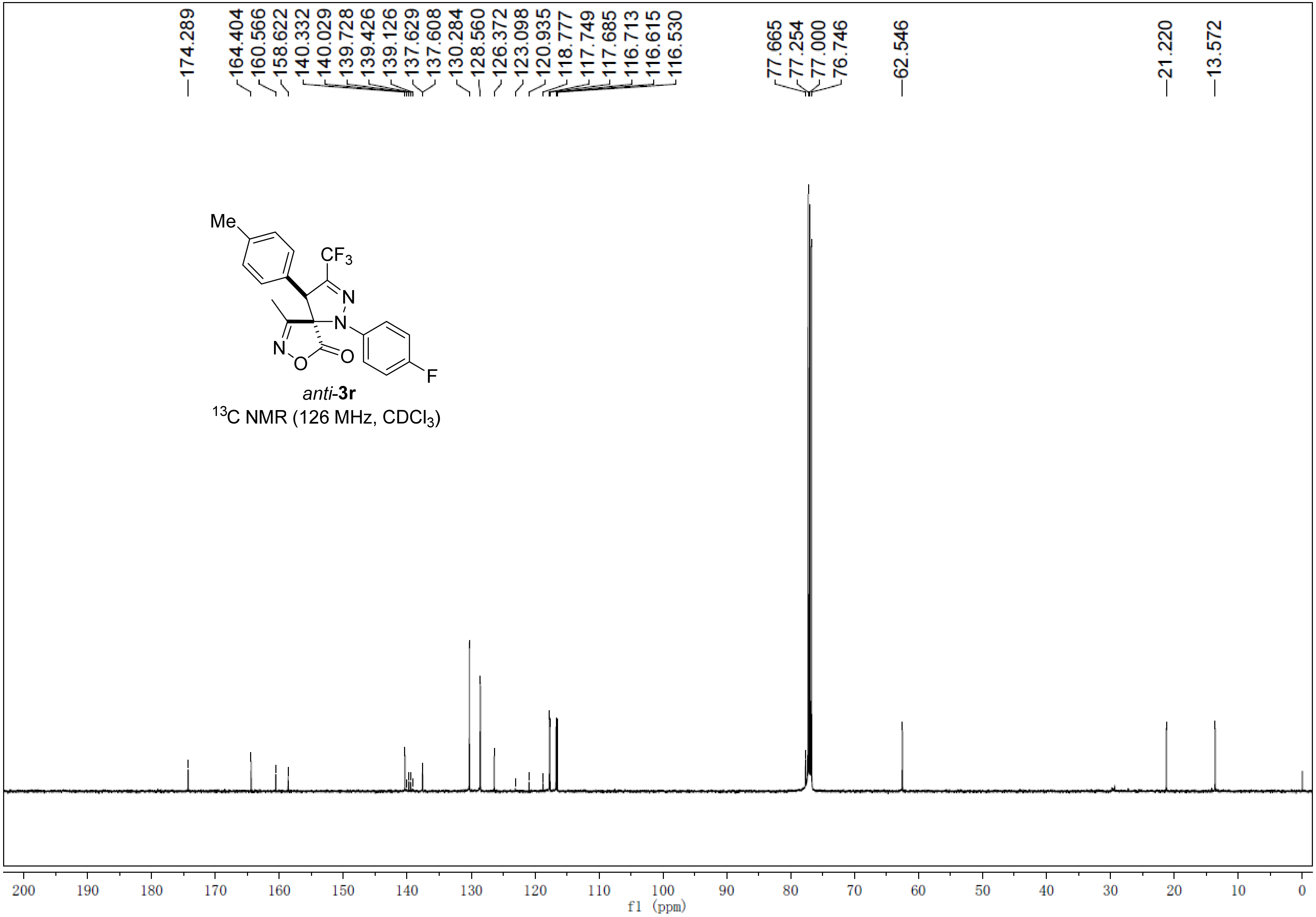
174.289
164.404
160.566
158.622
140.332
140.029
139.728
139.426
139.126
137.629
137.608
130.284
128.560
126.372
123.098
120.935
118.777
117.749
117.685
116.713
116.615
116.530
77.665
77.254
77.000
76.746
62.546
21.220
13.572
Me
CF_3
N
N
N
O
O
F
anti-**3r**
^{13}C NMR (126 MHz, $CDCl_3$)
200 190 180 170 160 150 140 130 120 110 100 90 80 70 60 50 40 30 20 10 0
f1 (ppm)

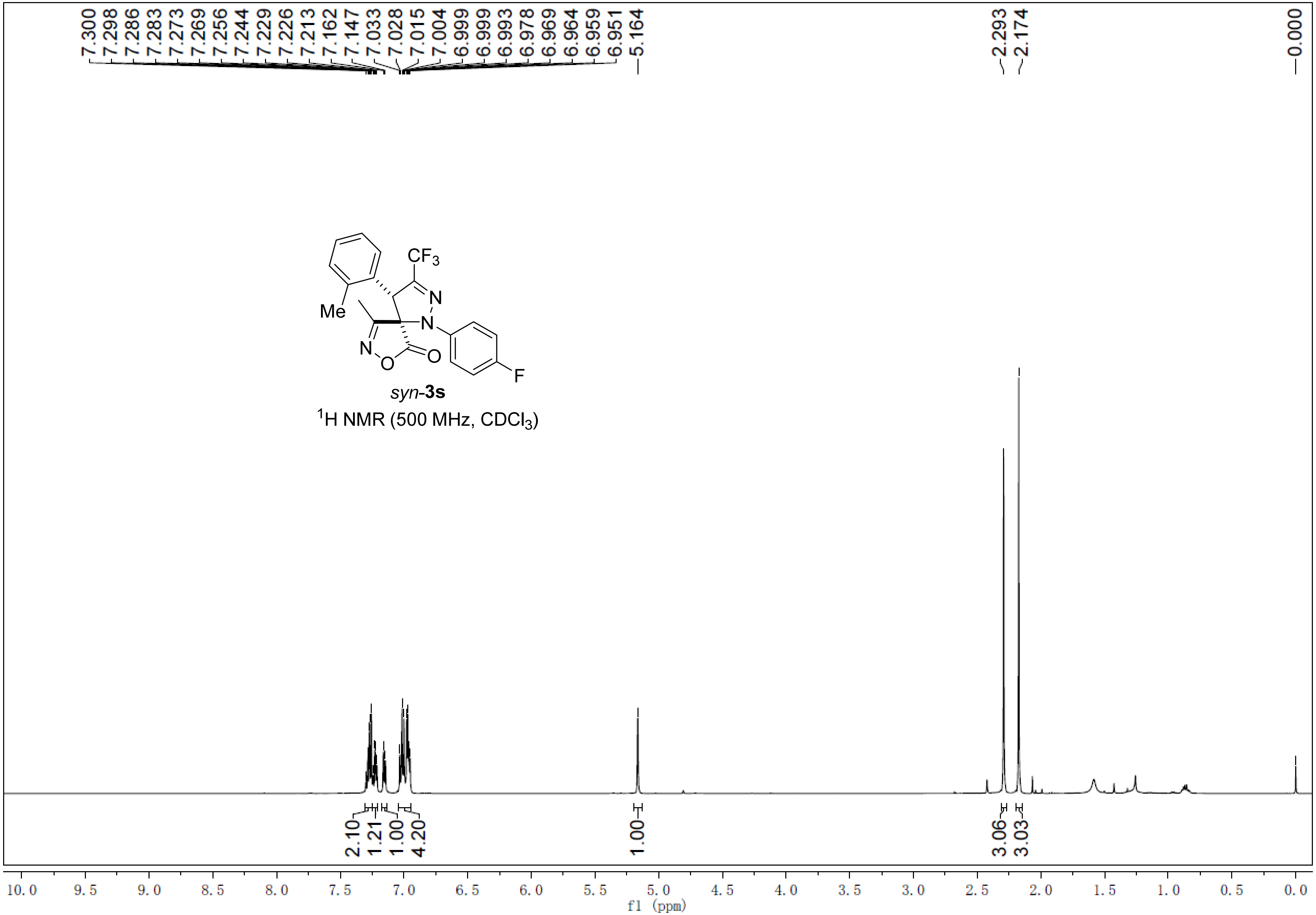
7.300 7.298 7.286 7.283 7.273 7.269 7.256 7.244 7.229 7.226 7.213 7.162 7.147 7.033 7.028 7.015 7.004 6.999 6.999 6.993 6.978 6.969 6.964 6.959 6.951 5.164
2.293 2.174
0.000
CF3
Me
N
N
N
O
O
F
syn-3s
1H NMR (500 MHz, CDCl3)
2.10 1.21 1.00 4.20
1.00
3.06 3.03
10.0 9.5 9.0 8.5 8.0 7.5 7.0 6.5 6.0 5.5 5.0 4.5 4.0 3.5 3.0 2.5 2.0 1.5 1.0 0.5 0.0
f1 (ppm)

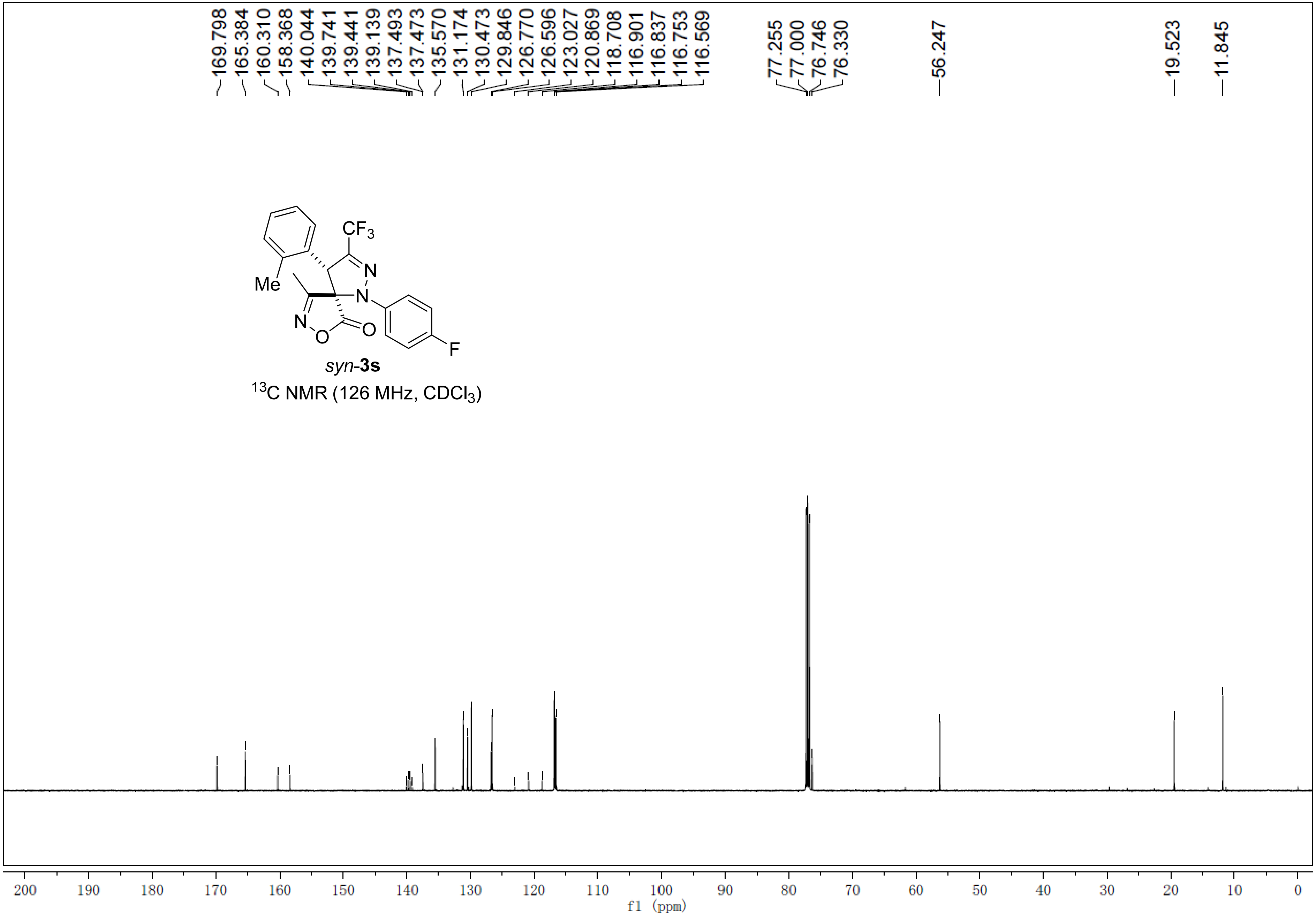
169.798
165.384
160.310
158.368
140.044
139.741
139.441
139.139
137.493
137.473
135.570
131.174
130.473
129.846
126.770
126.596
123.027
120.869
118.708
116.901
116.837
116.753
116.569
77.255
77.000
76.746
76.330
56.247
19.523
11.845
CF3
N
Me
N
N
O
O
F
syn-3s
13C NMR (126 MHz, CDCl3)
200 190 180 170 160 150 140 130 120 110 100 90 80 70 60 50 40 30 20 10 0
f1 (ppm)

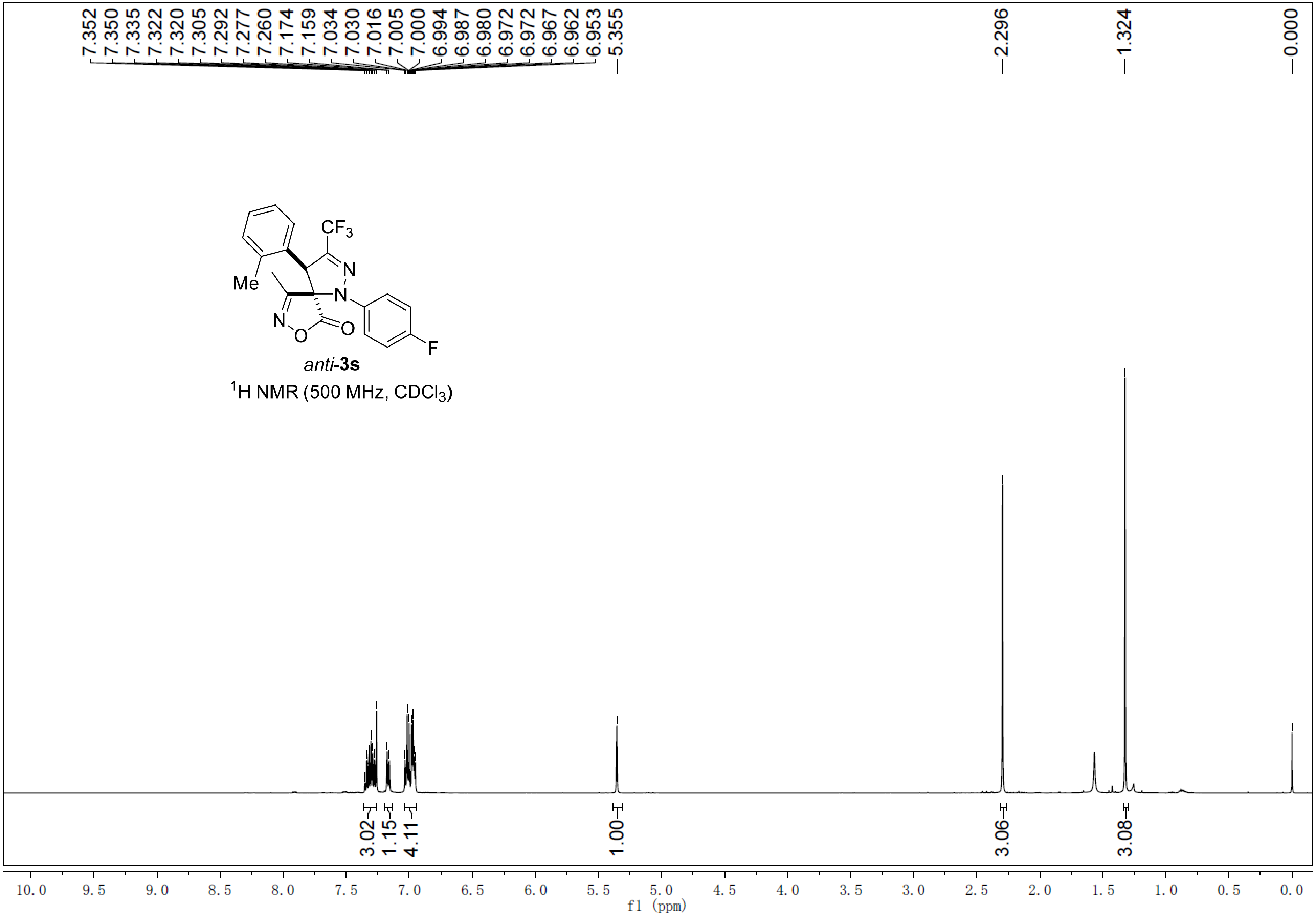
7.352
7.350
7.335
7.322
7.320
7.305
7.292
7.277
7.260
7.174
7.159
7.034
7.030
7.016
7.005
7.000
6.994
6.987
6.980
6.972
6.972
6.967
6.962
6.953
5.355
2.296
1.324
0.000
CF3
N
N
Me
N
O
O
F
anti-3s
1H NMR (500 MHz, CDCl3)
3.02
1.15
4.11
1.00
3.06
3.08
10.0
9.5
9.0
8.5
8.0
7.5
7.0
6.5
6.0
5.5
5.0
4.5
4.0
3.5
3.0
2.5
2.0
1.5
1.0
0.5
0.0
f1 (ppm)

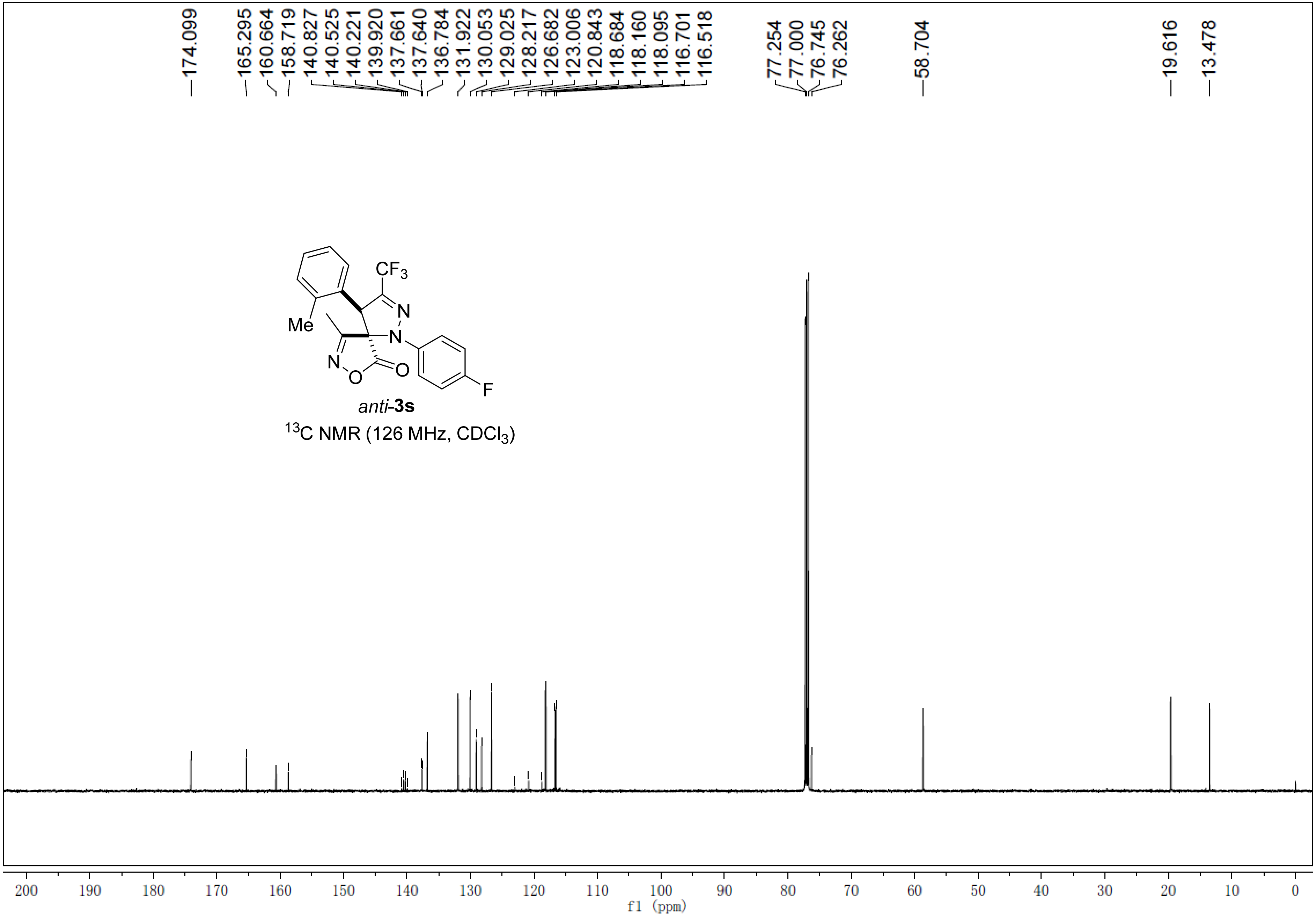
174.099
165.295
160.664
158.719
140.827
140.525
140.221
139.920
137.661
137.640
136.784
131.922
130.053
129.025
128.217
126.682
123.006
120.843
118.684
118.160
118.095
116.701
116.518
77.254
77.000
76.745
76.262
58.704
19.616
13.478
CF3
N
N
Me
N
O
O
F
anti-3s
13C NMR (126 MHz, CDCl3)
200
190
180
170
160
150
140
130
120
110
100
90
80
70
60
50
40
30
20
10
0
f1 (ppm)
